# Facile synthesis of 1*H*-imidazo[1,2-*b*]pyrazoles via a sequential one-pot synthetic approach

**DOI:** 10.3762/bjoc.10.243

**Published:** 2014-10-08

**Authors:** András Demjén, Márió Gyuris, János Wölfling, László G Puskás, Iván Kanizsai

**Affiliations:** 1 AVIDIN Ltd., Alsó Kikötő sor 11, Szeged, H-6726, Hungary; 2 Department of Organic Chemistry, University of Szeged, Dóm tér 8, H-6720, Szeged, Hungary; 3 AVICOR Ltd., Alsó Kikötő sor 11, Szeged, H-6726, Hungary

**Keywords:** Groebke–Blackburn–Bienaymé reaction, 1*H*-imidazo[1,2-*b*]pyrazole, isocyanide, multicomponent reaction, *N*-heterocycles

## Abstract

5-Aminopyrazole-4-carbonitrile and ethyl 5-aminopyrazole-4-carboxylate, as potential trifunctional building blocks are introduced in a facile, chemo- and regioselective multicomponent assembly of imidazo[1,2-*b*]pyrazoles via the Groebke–Blackburn–Bienaymé reaction (GBB reaction). Besides the synthetic elaboration of a green-compatible isocyanide-based access in three-component mode, we describe an operationally simple, one-pot two-step GBB protocol for the rapid construction of a 46 membered imidazo[1,2-*b*]pyrazole library with yields up to 83%.

## Introduction

**Scheme 1:**
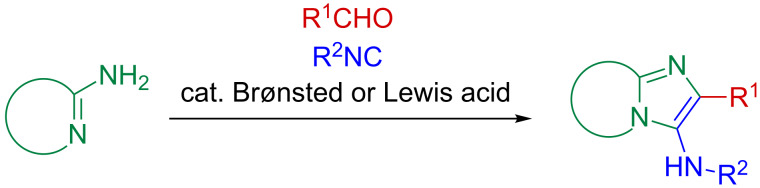
The conventional GBB-3CR.

For the relatively rapid design and construction of a diverse, large pharmacophore library, the basic concepts of diversity-oriented synthesis and isocyanide-based multicomponent reactions, such as the Ugi four-component reaction (U-4CR), can be adopted. The sequential combination of four species (amines, aldehydes, isocyanides and carboxylic acids) in a single-pot synthetic operation permits access to bisamide peptidomimetics through a highly electrophilic nitrilium intermediate [1–4]. Modification of the conventional U-4CR protocol in three-component fashion by the incorporation of bifunctional 2-amino-substituted heterocycles provides an alternative route via an intramolecular *N*-trapping procedure, leading to various *N*,*N*-heterobicyclic systems [5–12]. A number of bifunctional 2-aminoazoles, including thiazole [13–14] and 1,3,4-thiadiazole [15–16] derivatives, or 2-aminoazine-based heterocycles, such as pyridines [17–19], pyrimidines [20–24] and pyrazines [25–27], have recently been utilized as Groebke–Blackburn–Bienaymé three-component reaction (GBB-3CR) inputs (Scheme 1).

The transformations of either the 5-aminopyrazoles [28–29], or their 4-substituted ethoxycarbonyl [7,30–32] and carbonitrile [28,33–36] analogues via the GBB-3CR have not been appreciably examined so far. In the relevant literature [7,28–29,31–33], the products have predominantly been described as 5*H*-imidazo[1,2-*b*]pyrazoles with an *endo* double bond (and not as 1*H*-imidazo[1,2-*b*]pyrazoles), but without 2D NMR-based support. However, the GBB-3CR of functionalized pyrazoles might lead to the formation of two regioisomers [24] and four different tautomeric forms (5*H*- or 1*H*-imidazo[1,2-*b*]pyrazole with an endo- or exocyclic double bond) of each regioisomer. As presented [7,28–36], the “*endo”* 1*H*- and 5*H*-imidazo[1,2-*b*]pyrazoles were synthesized by the treatment of the corresponding amino substituted pyrazoles with aldehydes and isocyanides in the presence (5–30 mol %) of Lewis or Brønsted acid at ambient temperature or under heating (50–140 °C). The main disadvantages of this protocol involve long reaction times (3–18 hours) and requisite purification protocols (column chromatography and/or recrystallisation) besides limited diversity arising from the pyrazole starting material. As far as we are aware, a one-pot two-step process involving the in situ formation of the desired amino-substituted *N*-heterocycles such as C4 functionalized 5-aminopyrazoles, followed by GBB-3CR has not been described to date.

On the other hand, the imidazo[1,2-*b*]pyrazole core is definitely an attractive synthetic target, in view of its noteworthy pharmacological potential, which is strongly affected by the ring substitution pattern and the level of ring saturation. Among others, anti-inflammatory [37–38], antiviral [28,39] and antidiabetic [40] effects should be mentioned, besides the non-negligible cancer cell growth-inhibitory features of the corresponding compounds [30,34,41–42].

With respect to the current requirements of sustainable chemistry, our main aim was to design a streamlined and rapid green synthetic access route to a 1*H*-imidazo[1,2-*b*]pyrazole library in sequential one-pot protocol utilizing four components such as hydrazine hydrate, ethoxymethylene substituted malononitrile or ethyl cyanoacetate derivatives, isocyanides and aldehydes.

## Results and Discussion

In the initial stage, a model GBB-3CR was performed between 5-aminopyrazole-4-carbonitrile (**1a**), *p*-tolualdehyde (**2a**) and *tert*-butyl isocyanide (**3a**) in order to elucidate the structure of the product and investigate the regioselectivity. The synthesis of 5-aminopyrazole-4-carbonitrile (**1a**) was based on a literature method [43–44].

**Scheme 2:**
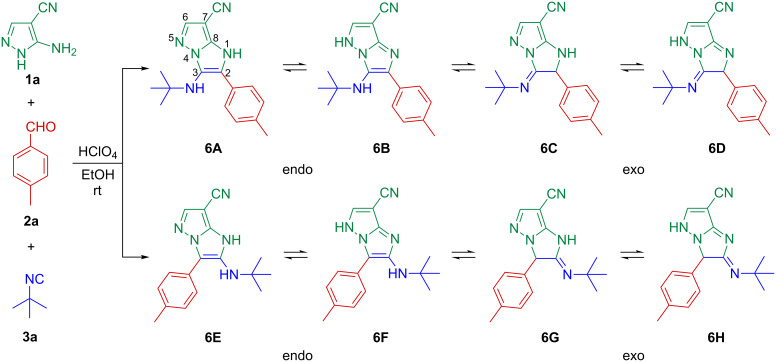
Plausible products **6A**–**H**.

A single product was observed in a yield of 59% during a reaction time of 15 min when a catalytic amount of HClO_4_ (20 mol %) was used as GBB-3CR promoter [7] in EtOH. 1D- and 2D NMR techniques (^1^H,^13^C-HSQC, ^1^H,^13^C-HMBC, ^1^H,^1^H-COSY and ^1^H,^1^H-NOESY) confirmed the exclusive presence of a 1*H*-imidazo[1,2-*b*]pyrazole core with an endocyclic double bond (**6A**), i.e., without the other possible regioisomeric and tautomeric forms **6B**–**H** (Scheme 2, see Supporting Information File 1 for detailed data and spectra).

For optimization, the 3CR synthesis of **6** was investigated under different catalytic conditions (Table 1). No reaction occurred in the absence of either a Brønsted or a Lewis acid catalyst (Table 1, entry 1). However, the use of Lewis acids, such as indium(III) salts or TMSCl, improved the reaction rate, with yields up to 67% (Table 1, entries 2–4). The GBB-3CR catalysed by Brønsted acids, including PTSA or HClO_4_, led to similar yields as on Lewis acid catalysis, though better results were obtained by using a catalytic amount of TFA in EtOH (Table 1, entries 5–7). From the aspect of an operationally simple green protocol, a mixture of water and EtOH as reaction medium yielded optimum results in terms of isolated yield, reaction time and mode of isolation (Table 1, entries 7–15). The one-pot 3CR of 5-aminopyrazole-4-carbonitrile (**1a**), *p*-tolualdehyde (**2a**) and *tert*-butyl isocyanide (**3a**) catalysed by TFA (20 mol %) in water/EtOH 1:1 furnished **6** isolated by simple filtration in a yield of 79% during 15 min.

**Table 1:** Solvent and catalyst screen of the GBB-3CR^a^.

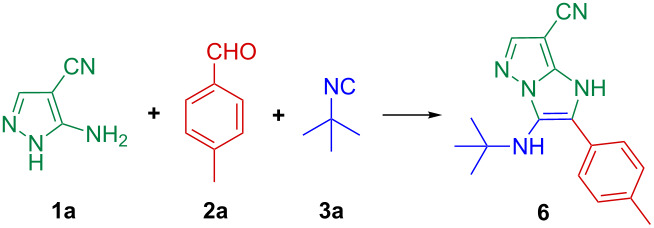					

Entry	Catalyst	Cat. load (mol %)	Solvent	Reaction time	Yield (%)

**1**	–	–	EtOH	> 72 h	0
**2**	In(OTf)_3_	20	EtOH	15 min	61^b^
**3**	InCl_3_	20	EtOH	15 min	67^b^
**4**	TMSCl	20	EtOH	15 min	64^b^
**5**	TsOH∙H_2_O	20	EtOH	15 min	52^b^
**6**	HClO_4_	20	EtOH	15 min	59^b^
**7**	TFA	20	EtOH	15 min	74^b^
**8**	TFA	20	CH_2_Cl_2_	15 min	35^c^
**9**	TFA	20	CH_2_Cl_2_	20 h	59^b^
**10**	TFA	20	CH_2_Cl_2_/MeOH 1:1	15 min	68^b^
**11**	TFA	20	MeCN	15 min	68^b^
**12**	TFA	20	THF	15 min	74^b^
**13**	TFA	20	MeOH	15 min	71^b^
**14**	TFA	20	H_2_O	15 min	63^c^
**15**	TFA	20	EtOH/H_2_O 1:1	15 min	79^b^
**16**	TFA	1	EtOH/H_2_O 1:1	36 h	46^b^
**17**	TFA	2	EtOH/H_2_O 1:1	20 h	62^b^
**18**	TFA	5	EtOH/H_2_O 1:1	1 h	75^b^
**19**	TFA	10	EtOH/H_2_O 1:1	25 min	76^b^

^a^Reaction conditions: **1a** (0.50 mmol), **2a** (0.55 mmol), **3a** (0.55 mmol), solvent (1 mL), room temperature. ^b^Isolated yield after simple filtration. ^c^Isolated yield after flash chromatography.

**Scheme 3:**
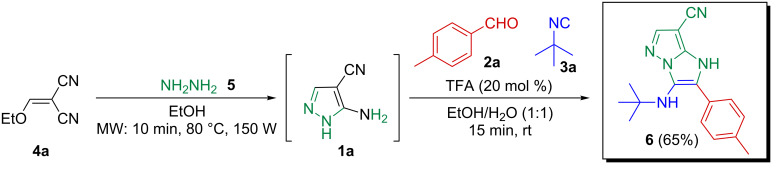
Synthesis of **6** via the sequential one-pot method.

These results led us to envisage a sequential one-pot access to 1*H*-imidazo[1,2-*b*]pyrazole species through the in situ microwave-assisted formation of **1a** followed by a GBB-3CR. A comparative study for the optimum synthesis of **6** revealed that the cyclocondensation of ethoxymethylene malononitrile (**4a**) with hydrazine (**5**) under microwave irradiation (80 °C, 150 W, 10 min, EtOH) proceeded with complete conversion (Scheme 3). It should be mentioned that the presence of water in this step resulted in a complex reaction mixture, moreover, the role of the reagent addition sequence was found to be crucial. The GBB reaction proceeded smoothly with acceptable efficacy during 15 min (overall yield of **6**: 65%), with the addition of water, aldehyde **2a**, a catalytic amount of TFA (20 mol %) and isocyanide **3a** to the solution of the preformed 5-aminopyrazole-4-carbonitrile (**1a**) at room temperature.

The well-established sequential one-pot protocol was then adopted to synthetize a series of 1*H*-imidazo[1,2-*b*]pyrazoles from selected aldehydes **2a–j** and isocyanide building blocks **3a–d** (Table 2). Following the microwave-assisted rapid formation of **1a**, the one-pot GBB reactions were completed during 10–60 min in yields of 23–83%. Unfortunately, limited substitution effect correlations could be established by employing aromatic aldehydes **2a–h**. The introduction of electron-donating substituents such as 4-Me or 2,4,6-tri-OMe (derived from aldehydes **2a** and **2h**) resulted in similar conversions as for **2b**, whereas the presence of two electron-withdrawing substituents as in **2e–g** resulted in decreased yields. α-Methylcinnamaldehyde (**2i**) as an uncommon isocyanide-based MCR component was successfully subjected to the GBB reaction, leading to the formation of the corresponding bicycles **38–41** in yields of 26–67%. All the reactions except those based on pivalaldehyde (**2j**) provided access to 1*H*-imidazo[1,2-*b*]pyrazoles through simple filtration. Of the aliphatic isocyanides **3a–d** applied, methyl isocyanoacetate (**3c**) often gave the lowest isolated yields, probably in consequence of self-trapping [45].

**Table 2:** Sequential one-pot GBB library generation^a^.

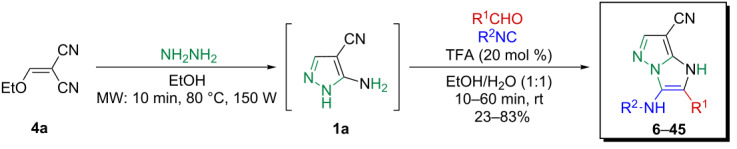					

Entry	R^1^CHO	R^2^NC		Product	Yield (%)

1	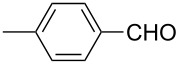 **2a**	*t*-BuNC	**3a**	**6**	79^b,c^ (65)^c^
2		*t*-octyl-NC	**3b**	**7**	66^c^
3		MeOOCCH_2_NC	**3c**	**8**	58^c^
4		CyNC	**3d**	**9**	75^c^
5	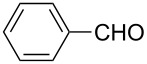 **2b**	*t*-BuNC	**3a**	**10**	68^c^
6		*t*-octyl-NC	**3b**	**11**	70^c^
7		MeOOCCH_2_NC	**3c**	**12**	70^c^
8		CyNC	**3d**	**13**	69^c^
9	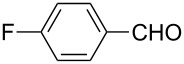 **2c**	*t*-BuNC	**3a**	**14**	67^c^
10		*t*-octyl-NC	**3b**	**15**	71^c^
11		MeOOCCH_2_NC	**3c**	**16**	41^c^
12		CyNC	**3d**	**17**	74^c^
13	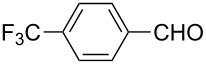 **2d**	*t*-BuNC	**3a**	**18**	63^c^
14		*t*-octyl-NC	**3b**	**19**	59^c^
15		MeOOCCH_2_NC	**3c**	**20**	35^c^
16		CyNC	**3d**	**21**	66^c^
17	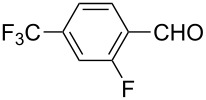 **2e**	*t*-BuNC	**3a**	**22**	59^c^
18		*t*-octyl-NC	**3b**	**23**	39^c^
19		MeOOCCH_2_NC	**3c**	**24**	23^c^
20		CyNC	**3d**	**25**	46^c^
21	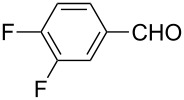 **2f**	*t*-BuNC	**3a**	**26**	59^c^
22		*t*-octyl-NC	**3b**	**27**	53^c^
23		MeOOCCH_2_NC	**3c**	**28**	28^c^
24		CyNC	**3d**	**29**	24^c^
25	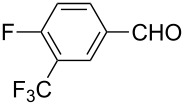 **2g**	*t*-BuNC	**3a**	**30**	53^c^
26		*t*-octyl-NC	**3b**	**31**	41^c^
27		MeOOCCH_2_NC	**3c**	**32**	33^c^
28		CyNC	**3d**	**33**	46^c^
29	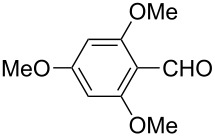 **2h**	*t*-BuNC	**3a**	**34**	70^c^
30		*t*-octyl-NC	**3b**	**35**	83^c^
31		MeOOCCH_2_NC	**3c**	**36**	48^c^
32		CyNC	**3d**	**37**	48^c^
33	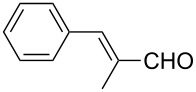 **2i**	*t*-BuNC	**3a**	**38**	61^c^
34		*t*-octyl-NC	**3b**	**39**	67^c^
35		MeOOCCH_2_NC	**3c**	**40**	26^c^
36		CyNC	**3d**	**41**	63^c^
37	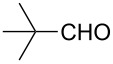 **2j**	*t*-BuNC	**3a**	**42**	50^d^
38		*t*-octyl-NC	**3b**	**43**	45^d^
39		MeOOCCH_2_NC	**3c**	**44**	47^d^
40		CyNC	**3d**	**45**	40^d^

^a^Reaction conditions: **4a** (0.50 mmol), **5** (0.55 mmol), ethanol (0.5 mL), MW (10 min, 80 °C, 150 W), then water (0.5 mL), **2a**–**j** (0.55 mmol), TFA (0.10 mmol), **3a**–**d** (0.55 mmol), room temperature, 10–60 min. ^b^Isolated yield from the GBB-3CR. ^c^Isolated yield after simple filtration. ^d^Isolated yield after flash chromatography.

To create diversely substituted 1*H*-imidazo[1,2-*b*]pyrazoles, the sequential one-pot GBB method has been extended by means of ethyl 2-cyano-3-ethoxyacrylate (**4b**), (1-ethoxyethylidene) malononitrile (**4c**) and ethyl (*E*)-2-cyano-3-ethoxycrotonate (**4d**). Application of these starting materials in the optimized protocol with a slight modification (elevated temperature was necessary for the microwave assisted preformation of pyrazole intermediates **1b**–**d**) afforded highly functionalized 1*H*-imidazo[1,2-*b*]pyrazole analogues **46**–**51** in yields of 54–79% (Table 3).

**Table 3:** Synthesis of highly substituted 1*H*-imidazo[1,2-*b*]pyrazoles^a^.

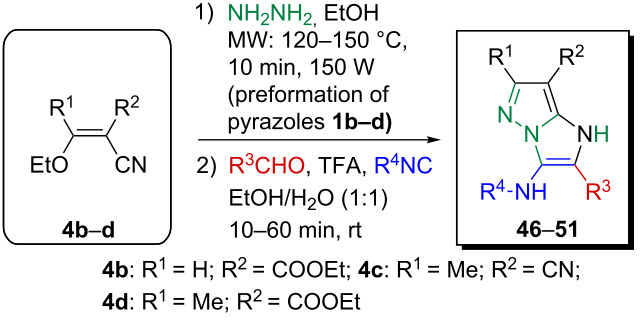						

Entry	R^1^	R^2^	R^3^CHO	R^4^NC	Product	Yield^b^ (%)

1	H	COOEt	**2a**	**3a**	**46**	54
2	H	COOEt	**2b**	**3b**	**47**	56
3	Me	CN	**2a**	**3a**	**48**	79
4	Me	CN	**2c**	**3c**	**49**	57
5	Me	COOEt	**2a**	**3a**	**50**	74
6	Me	COOEt	**2i**	**3b**	**51**	59

^a^Reaction conditions: **4b**–**d** (0.50 mmol), **5** (0.55 mmol), ethanol (0.5 mL), MW (10 min; **4b**: 150 °C, **4c,d**: 120 °C; 150 W), then water (0.5 mL), **2a–c,i** (0.55 mmol), TFA (0.10 mmol), **3a**–**c** (0.55 mmol), room temperature, 10–60 min. ^b^Isolated yield after simple filtration.

## Conclusion

We have described here the development of a de novo and facile one-pot, two-step GBB method. The established protocol allowed the rapid synthesis of a 46-membered 1*H*-imidazo[1,2-*b*]pyrazole library with isolated yields up to 83%. Following the microwave-aided formation of functionalized 5-aminopyrazoles, the GBB-3CR transformations occurred during 10–60 min under mild conditions. This protocol offers operationally simple, green access to highly substituted 1*H*-imidazo[1,2-*b*]pyrazoles with easily variable substitution pattern and does not require complex purification techniques.

## Supporting Information

File 1 Experimental and characterisation data.
