# Supplementary material for: Facile synthesis of 1H-imidazo[1,2-b]pyrazoles via a sequential one-pot synthetic approach
Source: Beilstein J Org Chem. 2014 Oct 8;10:2338–44. doi: 10.3762/bjoc.10.243 (PMC4222397; doi:10.3762/bjoc.10.243)

Supporting Information

for

Facile synthesis of 1*H*-imidazo[1,2-*b*]pyrazoles via a sequential one-pot synthetic approach

András Demjén[1,3], Márió Gyuris[1], János Wölfling[3], László G. Puskás[1,2] and Iván Kanizsai[1*§]

Address: [1]AVIDIN Ltd., Alsó Kikötő sor 11, Szeged, H-6726, Hungary, [2]AVICOR Ltd., Alsó Kikötő sor 11, Szeged, H-6726, Hungary and [3]Department of Organic Chemistry, University of Szeged, Dóm tér 8, H-6720, Szeged, Hungary

Email: Iván Kanizsai* - i.kanizsai@avidinbiotech.com

*Corresponding author

[§]Tel.: +36-62/202107; Fax: +36-62/202108

Experimental and characterisation data

Index

1. Preparation and characterization of compounds S2

2. ^{1}H-^{13}C HSQC, ^{1}H-^{13}C HMBC, ^{1}H-^{1}H COSY and ^{1}H-^{1}H NOESY NMR spectra of compound **6** S21

3. ^{1}H and ^{13}C NMR spectra for all compounds S27

1. Preparation and characterization of compounds

^{1}H NMR and ^{13}C NMR spectra were recorded at 500.13 and 125.76 MHz, in DMSO-d_6 at 298.0 K by a Bruker Avance 500 spectrometer, using tetramethylsilane as an internal standard. Chemical shifts (δ) and coupling constants (J) are given in ppm and Hz, respectively. Melting points were determined on a Stuart SMP10 apparatus and are uncorrected. Mass spectra were obtained by means of a Waters Acquity SQD (Waters MS Technologies, Manchester, UK) mass spectrometer in ESI mode. Samples were injected to the MS using flow injection method, water:MeCN 1:1, 0.1% TFA was used as solvent. The reactions were monitored by TLC on silica gel plates (Kieselgel 60 F_{254}). Microwave-assisted experiments were conducted using a CEM Discover System in closed vessels under magnetic stirring. All solvents and reagents were commercially available and used without further purification unless otherwise noted.

General procedure for the synthesis of 1*H*-imidazo[1,2-*b*]pyrazoles 6–51

Hydrazine monohydrate (26.7 µl, 0.55 mmol) was added to a solution of **(**ethoxymethylene)malonic acid derivatives **4a-d** (0.50 mmol) in EtOH (0.5 ml), and the solution was subjected to microwave irradiation (**4a:** 80°C, **4b:** 150°C, **4c,d:** 120°C; 150W; 10 min). Afterwards, water (0.5 ml), aldehydes **2a–j** (0.55 mmol), TFA (7.7 µl, 0.10 mmol) and isocyanides **3a–d** (0.55 mmol) were sequentially added to the solution, and the reaction was stirred for 10–60 min at room temperature. The precipitated product was filtered, washed with hexane or diethyl ether (1–3 ml) and dried in vacuo, yielding the corresponding final products **6–41** and **46–51**. In case of **42–45**, the crude reaction mixtures were purified by flash chromatography with hexane-EtOAc as eluent.

3-(*tert*-Butylamino)-2-(*p*-tolyl)-1*H*-imidazo[1,2-*b*]pyrazole-7-carbonitrile (6)

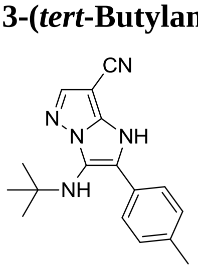

6

Pale yellow crystals; 95 mg (65%); m.p.: 234–236°C; TLC: R_f = 0.66 (hexane/EtOAc, v/v = 2:1); ^{1}H NMR (500 MHz, DMSO-d_6) δ (ppm): 1.04 (s, 9H), 2.31 (s, 3H), 4.41 (s, 1H), 7.24 (d, J = 7.4 Hz, 2H), 7.85 (d, J = 7.5 Hz, 2H), 8.00 (s, 1H), 12.28 (s, 1H); ^{13}C NMR (126 MHz,

DMSO-d_6) δ (ppm): 20.8, 30.1, 54.7, 65.3, 115.3, 122.3, 125.0, 126.2, 127.2, 129.0, 137.0, 138.1, 145.1; MS (ESI): 294.1 $[M+H]^+$.

2-(*p*-Tolyl)-3-((2,4,4-trimethylpentan-2-yl)amino)-1*H*-imidazo[1,2-*b*]pyrazole-7-carbonitrile (7)

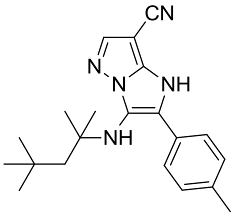

7

White crystals; 115 mg (66%); m.p.: 203–205°C; TLC: R_f = 0.75 (hexane/EtOAc, v/v = 2:1); ^{1}H NMR (500 MHz, DMSO-d_6) δ (ppm): 0.97 (s, 9H), 0.99 (s, 6H), 1.55 (s, 2H), 2.32 (s, 3H), 4.17 (s, 1H), 7.24 (d, *J* = 7.7 Hz, 2H), 7.79 (d, *J* = 7.9 Hz, 2H), 8.00 (s, 1H), 12.27 (s, 1H); ^{13}C NMR (126 MHz, DMSO-d_6) δ (ppm): 20.9, 29.0, 31.3, 31.6, 55.5, 58.8, 65.2, 115.4, 122.1, 125.2, 126.7, 127.3, 129.0, 137.1, 138.1, 145.1; MS (ESI): 350.2 $[M+H]^+$.

Methyl 2-((7-cyano-2-(*p*-tolyl)-1*H*-imidazo[1,2-*b*]pyrazole-3-yl)amino)acetate (8)

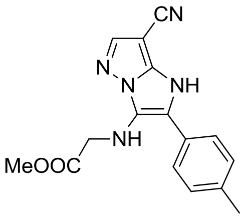

8

Pale yellow crystals; 90 mg (58%); m.p.: 189–192°C; TLC: R_f = 0.36 (hexane/EtOAc, v/v = 2:1); ^{1}H NMR (500 MHz, DMSO-d_6) δ (ppm): 2.32 (s, 3H), 3.55 (s, 3H), 4.16 (d, *J* = 4.7 Hz, 2H), 5.65 (t, *J* = 4.9 Hz, 1H), 7.26 (d, *J* = 7.8 Hz, 2H), 7.69 (d, *J* = 7.9 Hz, 2H), 8.02 (s, 1H); 12.15 (s, 1H); ^{13}C NMR (126 MHz, DMSO-d_6) δ (ppm): 20.8, 46.1, 51.6, 65.3, 115.3, 116.4, 124.5, 125.7, 126.9, 129.3, 136.4, 138.0, 145.9, 171.7; MS (ESI): 310.1 $[M+H]^+$.

3-(Cyclohexylamino)-2-(*p*-tolyl)-1*H*-imidazo[1,2-*b*]pyrazole-7-carbonitrile (9)

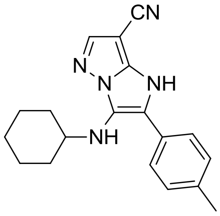

9

Pale yellow crystals; 120 mg (75%); m.p.: 230–232°C; TLC: R_f = 0.66 (hexane/EtOAc, v/v = 2:1); ^{1}H NMR (500 MHz, DMSO-d_6) δ (ppm): 1.05–1.23 (m, 5H), 1.42–1.50 (m, 1H), 1.56–1.64 (m, 2H), 1.69–1.76 (m, 2H), 2.31 (s, 3H), 3.24–3.31 (m, 1H), 4.67 (d, *J* = 4.6 Hz, 1H), 7.25 (d, *J* = 7.4 Hz, 2H), 7.77 (d, *J* = 7.6 Hz, 2H), 8.01 (s, 1H), 12.20 (s, 1H); ^{13}C NMR (126 MHz, DMSO-d_6) δ (ppm): 20.8, 24.2, 25.5, 33.1, 54.4, 65.4, 115.3, 121.0, 123.8, 125.4, 127.0, 129.2, 136.6, 138.1, 145.6; MS (ESI): 320.1 $[M+H]^+$.

3-(*tert*-Butylamino)-2-phenyl-1*H*-imidazo[1,2-*b*]pyrazole-7-carbonitrile (10)

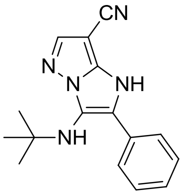

10

Pale yellow crystals; 95 mg (68%); m.p.: 209–211°C; TLC: R_f = 0.64 (hexane/EtOAc, v/v = 2:1); ^{1}H NMR (500 MHz, DMSO-d_6) δ (ppm): 1.05 (s, 9H), 4.45 (s, 1H), 7.31 (t, *J* = 7.0 Hz, 1H), 7.43 (t, *J* = 7.2 Hz, 2H), 7.96 (d, *J* = 7.6 Hz, 2H), 8.01 (s, 1H), 12.35 (s, 1H); ^{13}C NMR (126 MHz, DMSO-d_6) δ (ppm): 30.1, 54.8, 65.4, 115.3, 122.7, 124.8, 126.3, 127.6, 128.5, 130.1, 138.3, 145.3; MS (ESI): 280.1 $[M+H]^+$.

2-Phenyl-3-((2,4,4-trimethylpentan-2-yl)amino)-1*H*-imidazo[1,2-*b*]pyrazole-7-carbonitrile (11)

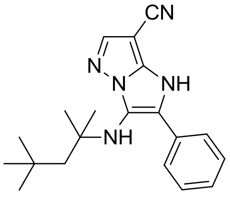

11

White crystals; 117 mg (70%); m.p.: 212–214°C; TLC: R_f = 0.71 (hexane/EtOAc, v/v = 2:1); ^{1}H NMR (500 MHz, DMSO-d_6) δ (ppm): 0.96 (s, 9H), 0.99 (s, 6H), 1.54 (s, 2H), 4.22 (s, 1H), 7.32 (t, *J* = 7.1 Hz, 1H), 7.43 (t, *J* = 7.4 Hz, 2H), 7.89 (d, *J* = 7.6 Hz, 2H), 8.01 (s, 1H), 12.34 (s, 1H); ^{13}C NMR (126 MHz, DMSO-d_6) δ (ppm): 29.0, 31.3, 31.6, 55.4, 58.8, 65.3, 115.3, 122.5, 125.1, 126.8, 127.7, 128.4, 130.1, 138.2, 145.2; MS (ESI): 336.2 $[M+H]^+$.

Methyl 2-((7-cyano-2-phenyl-1*H*-imidazo[1,2-*b*]pyrazol-3-yl)amino)acetate (12)

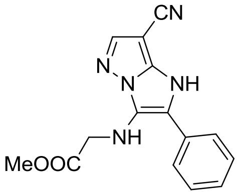

12

White crystals; 103 mg (70%); m.p.: 200–201°C; TLC: R_f = 0.31 (hexane/EtOAc, v/v = 2:1); ^{1}H NMR (500 MHz, DMSO-d_6) δ (ppm): 3.55 (s, 3H), 4.18 (d, *J* = 6.0 Hz, 2H), 5.76 (t, *J* = 5.8 Hz, 1H), 7.29 (t, *J* = 7.1 Hz, 1H), 7.45 (t, *J* = 7.4 Hz, 2H), 7.79 (d, *J* = 7.5 Hz, 2H), 8.03 (s, 1H), 12.19 (s, 1H); ^{13}C NMR (126 MHz, DMSO-d_6) δ (ppm): 46.0, 51.6, 65.3, 115.2, 116.0, 125.0, 125.7, 126.9, 128.8, 129.8, 138.2, 146.0, 171.8; MS (ESI): 296.1 $[M+H]^+$.

3-(Cyclohexylamino)-2-phenyl-1*H*-imidazo[1,2-*b*]pyrazole-7-carbonitrile (13)

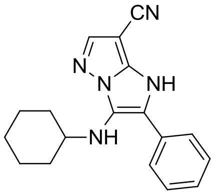

13

Pale yellow crystals; 106 mg (69%); m.p.: 206–208°C; TLC: R_f = 0.62 (hexane/EtOAc, v/v = 2:1); ^{1}H NMR (500 MHz, DMSO-d_6) δ (ppm): 1.04–1.25 (m, 6H), 1.42–1.51 (m, 1H), 1.57–1.65 (m, 2H), 1.70–1.78 (m, 2H), 4.76 (d, *J* = 4.8 Hz, 1H), 7.29 (t, *J* = 7.0 Hz, 1H), 7.44 (t, *J* = 7.4 Hz, 2H), 7.88 (d, *J* = 7.5 Hz, 2H), 8.03 (s, 1H), 12.26 (s, 1H); ^{13}C NMR (126 MHz, DMSO-d_6) δ (ppm): 24.3, 25.5, 33.2, 54.4, 65.4, 115.3, 120.5, 124.4, 125.5, 127.1, 128.7, 129.9, 138.2, 145.7; MS (ESI): 306.1 $[M+H]^+$.

3-(*tert*-Butylamino)-2-(4-fluorophenyl)-1*H*-imidazo[1,2-*b*]pyrazole-7-carbonitrile (14)

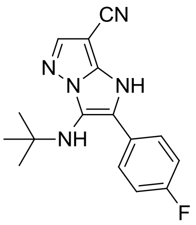

14

White crystals; 100 mg (67%); m.p.: 231–233°C; TLC: R_f = 0.62 (hexane/EtOAc, v/v = 2:1); ^{1}H NMR (500 MHz, DMSO-d_6) δ (ppm): 1.04 (s, 9H), 4.48 (s, 1H), 7.30 (t, *J* = 8.6 Hz, 2H), 7.97–8.04 (m, 3H), 12.39 (s, 1H); ^{13}C NMR (126 MHz, DMSO-d_6) δ (ppm): 30.1, 54.7, 65.4,

115.3, 115.5 (d, J = 21.6 Hz), 122.6, 124.1, 126.6, 128.5 (d, J = 7.8 Hz), 138.3, 145.3, 161.4 (d, J = 245.6 Hz); MS (ESI): 298.1 $[M+H]^+$.

2-(4-Fluorophenyl)-3-((2,4,4-trimethylpentan-2-yl)amino)-1*H*-imidazo[1,2-*b*]pyrazole-7-carbonitrile (15)

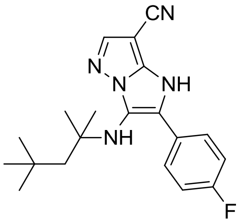

15

White crystals; 126 mg (71%); m.p.: 204–205°C; TLC: R_f = 0.70 (hexane/EtOAc, v/v = 2:1); ^{1}H NMR (500 MHz, DMSO-d_6) δ (ppm): 0.96 (s, 9H), 0.99 (s, 6H), 1.53 (s, 2H), 4.25 (s, 1H), 7.30 (t, J = 8.5 Hz, 2H), 7.93 (dd, J = 7.4, 5.7 Hz, 2H), 8.02 (s, 1H), 12.37 (s, 1H); ^{13}C NMR (126 MHz, DMSO-d_6) δ (ppm): 29.0, 31.3, 31.6, 55.4, 58.8, 65.3, 115.3, 115.4 (d, J = 21.5 Hz), 122.4, 124.4, 126.6, 129.1 (d, J = 7.8 Hz), 138.2, 145.2, 161.6 (d, J = 245.2 Hz); MS (ESI): 354.2 $[M+H]^+$.

Methyl 2-((7-cyano-2-(4-fluorophenyl)-1*H*-imidazo[1,2-*b*]pyrazol-3-yl)amino)acetate (16)

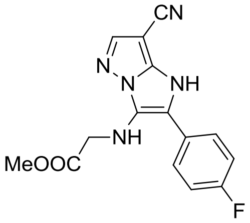

16

White crystals; 64 mg (41%); m.p.: 210–212°C; TLC: R_f = 0.35 (hexane/EtOAc, v/v = 2:1); ^{1}H NMR (500 MHz, DMSO-d_6) δ (ppm): 3.56 (s, 3H), 4.17 (d, J = 5.9 Hz, 2H), 5.76 (t, J = 5.8 Hz, 1H), 7.31 (t, J = 8.4 Hz, 2H), 7.86 (dd, J = 7.1, 5.1 Hz, 2H), 8.04 (s, 1H), 12.23 (s, 1H); ^{13}C NMR (126 MHz, DMSO-d_6) δ (ppm): 46.0, 51.6, 65.3, 115.2, 115.7 (d, J = 21.9 Hz), 124.7, 126.3, 128.0 (d, J = 7.7 Hz), 138.2, 146.0, 161.1 (d, J = 244.6 Hz), 171.8; MS (ESI): 314.1 $[M+H]^+$.

3-(Cyclohexylamino)-2-(4-fluorophenyl)-1*H*-imidazo[1,2-*b*]pyrazole-7-carbonitrile (17)

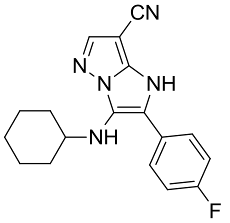

17

White crystals; 120 mg (74%); m.p.: 222–223°C; TLC: R_f = 0.64 (hexane/EtOAc, v/v = 2:1); ^{1}H NMR (500 MHz, DMSO-d_6) δ (ppm): 1.06–1.24 (m, 5H), 1.44–1.51 (m, 1H), 1.57–1.65 (m, 2H), 1.70–1.77 (m, 2H), 4.78 (d, *J* = 4.0 Hz, 1H), 7.31 (t, *J* = 8.1 Hz, 2H), 7.88–7.96 (m, 2H), 8.03 (s, 1H), 12.30 (s, 1H); ^{13}C NMR (126 MHz, DMSO-d_6) δ (ppm): 24.2, 25.5, 33.1, 54.3, 65.4, 115.3, 115.6 (d, *J* = 21.6 Hz), 120.2, 124.1, 126.3, 127.7 (d, *J* = 7.7 Hz), 138.2, 145.7, 161.2 (d, *J* = 245.1 Hz); MS (ESI): 324.1 $[M+H]^+$.

3-(*tert*-Butylamino)-2-(4-(trifluoromethyl)phenyl)-1*H*-imidazo[1,2-*b*]pyrazole-7-carbonitrile (18)

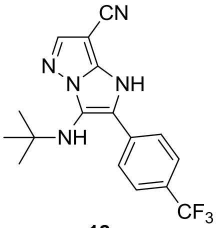

18

White crystals; 109 mg (63%); m.p.: 247–248°C; TLC: R_f = 0.66 (hexane/EtOAc, v/v = 2:1); ^{1}H NMR (500 MHz, DMSO-d_6) δ (ppm): 1.07 (s, 9H), 4.63 (s, 1H), 7.80 (d, *J* = 7.8 Hz, 2H), 8.06 (s, 1H), 8.21 (d, *J* = 7.8 Hz, 2H), 12.57 (s, 1H); ^{13}C NMR (126 MHz, DMSO-d_6) δ (ppm): 30.1, 55.1, 65.5, 115.1, 123.4, 124.1, 125.4 (d, *J* = 2.3 Hz), 126.5, 127.5 (q, *J* = 31.7 Hz), 134.2, 138.8, 145.8; MS (ESI): 348.0 $[M+H]^+$.

2-(4-(Trifluoromethyl)phenyl)-3-((2,4,4-trimethylpentan-2-yl)amino)-1*H*-imidazo[1,2-*b*]pyrazole-7-carbonitrile (19)

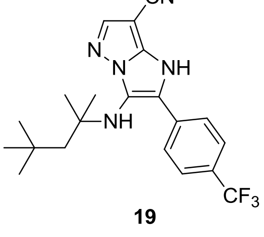

19

White crystals; 118 mg (59%); m.p.: 214–216°C; TLC: R_f = 0.75 (hexane/EtOAc, v/v = 2:1); ^{1}H NMR (500 MHz, DMSO-d_6) δ (ppm): 0.97 (s, 9H), 1.02 (s, 6H), 1.57 (s, 2H), 4.44 (s, 1H), 7.80 (d, *J* = 8.1 Hz, 2H), 8.06 (s, 1H), 8.14 (d, *J* = 8.0 Hz, 2H), 12.55 (s, 1H); ^{13}C NMR (126 MHz, DMSO-d_6) δ (ppm): 29.0, 31.3, 31.6, 55.4, 59.1, 65.4, 115.1, 123.6, 123.8, 125.3 (d, *J* = 2.1 Hz), 127.1, 127.6 (q, *J* = 31.5 Hz), 134.3, 138.8, 145.7; MS (ESI): 404.1 $[M+H]^+$.

Methyl 2-((7-cyano-2-(4-(trifluoromethyl)phenyl)-1*H*-imidazo[1,2-*b*]pyrazole-3-yl)-amino)acetate (20)

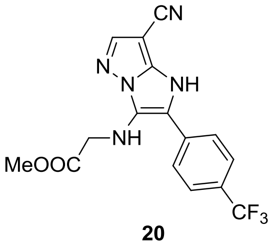

20

White crystals; 64 mg (35%); m.p.: 191–193°C; TLC: R_f = 0.42 (hexane/EtOAc, v/v = 2:1); ^{1}H NMR (500 MHz, DMSO-d_6) δ (ppm): 3.56 (s, 3H), 4.26 (d, *J* = 5.7 Hz, 2H), 6.10 (t, *J* = 5.8 Hz, 1H), 7.79 (d, *J* = 8.1 Hz, 2H), 7.99 (d, *J* = 8.0 Hz, 2H), 8.07 (s, 1H), 12.37 (s, 1H); ^{13}C NMR (126 MHz, DMSO-d_6) δ (ppm): 45.7, 51.6, 65.3, 114.0, 115.0, 124.3 (q, *J* = 271.5 Hz), 125.6, 125.7, 126.6 (q, *J* = 32.2 Hz), 126.6, 134.0, 138.9, 146.6, 171.7; MS (ESI): 364.0 $[M+H]^+$.

3-(Cyclohexylamino)-2-(4-(trifluoromethyl)phenyl)-1*H*-imidazo[1,2-*b*]pyrazole-7-carbonitrile (21)

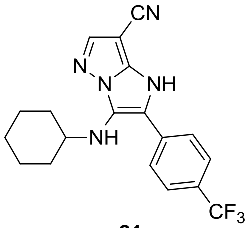

21

White crystals; 123 mg (66%); m.p.: 242–243°C; TLC: R_f = 0.66 (hexane/EtOAc, v/v = 2:1); ^{1}H NMR (500 MHz, DMSO-d_6) δ (ppm): 1.06–1.29 (m, 5H), 1.45–1.52 (m, 1H), 1.58–1.68 (m, 2H), 1.72–1.81 (m, 2H), 3.36–3.48 (m, 1H), 5.05 (d, *J* = 4.0 Hz, 1H), 7.80 (d, *J* = 6.6 Hz, 2H), 8.02–8.14 (m, 3H), 12.45 (s, 1H); ^{13}C NMR (126 MHz, DMSO-d_6) δ (ppm): 24.3, 25.4, 33.2, 54.3, 65.5, 115.1, 118.5, 125.5, 125.6, 126.0, 126.8 (q, *J* = 32.2 Hz), 134.0, 138.9, 146.3; MS (ESI): 374.0 $[M+H]^+$.

3-(*tert*-Butylamino)-2-(2-fluoro-4-(trifluoromethyl)phenyl)-1*H*-imidazo[1,2-*b*]pyrazole-7-carbonitrile (22)

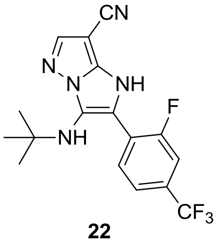

22

White crystals; 108 mg (59%); m.p.: 209–211°C; TLC: R_f = 0.65 (hexane/EtOAc, v/v = 2:1); ^{1}H NMR (500 MHz, DMSO-d_6) δ (ppm): 0.98 (s, 9H), 4.47 (s, 1H), 7.70 (d, *J* = 7.8 Hz, 1H), 7.85 (d, *J* = 10.3 Hz, 1H), 8.05–8.10 (m, 2H), 12.52 (s, 1H); ^{13}C NMR (126 MHz, DMSO-d_6) δ (ppm): 29.8, 54.6, 65.4, 113.7 (d, *J* = 24.9 Hz), 115.0, 117.7, 121.4, 122.1 (d, *J* = 13.7 Hz), 123.3 (q, *J* = 273 Hz), 124.9, 130.4 (qd, *J* = 32.6, 7.6 Hz), 132.3, 138.9, 145.9, 158.6 (d, *J* = 250.6 Hz); MS (ESI): 366.1 $[M+H]^+$.

2-(2-Fluoro-4-(trifluoromethyl)phenyl)-3-((2,4,4-trimethylpentan-2-yl)amino)-1*H*-imidazo[1,2-*b*]pyrazole-7-carbonitrile (23)

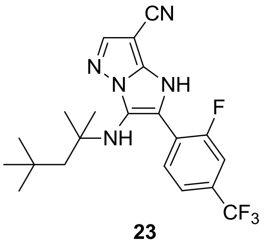

23

White crystals; 82 mg (39%); m.p.: 179–181°C; TLC: R_f = 0.75 (hexane/EtOAc, v/v = 2:1); ^{1}H NMR (500 MHz, DMSO-d_6) δ (ppm): 0.90 (s, 9H), 0.96 (s, 6H), 1.42 (s, 2H), 4.20 (s, 1H), 7.71 (d, *J* = 7.0 Hz, 1H), 7.87 (d, *J* = 9.5 Hz, 1H), 7.98–8.05 (m, 1H), 8.08 (s, 1H), 12.54 (s, 1H); ^{13}C NMR (126 MHz, DMSO-d_6) δ (ppm): 28.9, 31.2, 31.5, 55.1, 58.5, 65.3, 113.6 (d, *J* = 23.0 Hz), 115.0, 117.8, 121.4, 122.2 (d, *J* = 13.3 Hz), 124.7, 130.1–130.8 (m), 132.7, 138.9, 145.8, 158.8 (d, *J* = 250.5 Hz); MS (ESI): 422.1 $[M+H]^+$.

Methyl 2-((7-cyano-2-(2-fluoro-4-(trifluoromethyl)phenyl)-1*H*-imidazo[1,2-*b*]pyrazol-3-yl)amino)acetate (24)

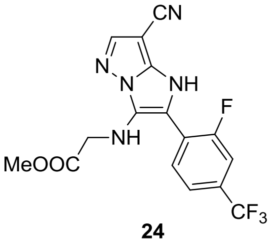

24

White crystals; 44 mg (23%); m.p.: 181–182°C; TLC: R_f = 0.47 (hexane/EtOAc, v/v = 2:1); ^{1}H NMR (500 MHz, DMSO-d_6) δ (ppm): 3.53 (s, 3H), 4.20 (d, *J* = 6.1 Hz, 2H), 5.98 (t, *J* = 5.9 Hz, 1H), 7.69 (d, *J* = 7.9 Hz, 1H), 7.82 (d, *J* = 10.4 Hz, 1H), 7.94 (t, *J* = 7.6 Hz, 1H), 8.09 (s, 1H), 12.26 (s, 1H); ^{13}C NMR (126 MHz, DMSO-d_6) δ (ppm): 45.4, 51.6, 65.2, 106.5, 113.6 (d, *J* = 23.3 Hz), 115.0, 121.6, 122.0 (d, *J* = 13.1 Hz), 127.7, 128.9–129.8 (m), 131.2, 139.1, 146.7, 158.2 (d, *J* = 249.9 Hz), 171.5; MS (ESI): 382.1 $[M+H]^+$.

3-(Cyclohexylamino)-2-(2-fluoro-4-(trifluoromethyl)phenyl)-1*H*-imidazo[1,2-*b*]pyrazole-7-carbonitrile (25)

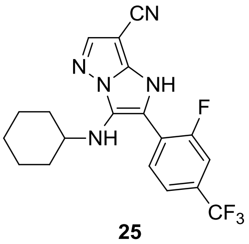

25

White crystals; 90 mg (46%); m.p.: 222–224°C; TLC: R_f = 0.70 (hexane/EtOAc, v/v = 2:1); ^{1}H NMR (500 MHz, DMSO-d_6) δ (ppm): 1.03–1.21 (m, 5H), 1.46–1.53 (m, 1H), 1.57–1.65 (m, 2H), 1.72–1.79 (m, 2H), 3.53–3.62 (m, 1H), 5.04 (d, *J* = 7.3 Hz, 1H), 7.68 (d, *J* = 7.6 Hz, 1H), 7.80 (d, *J* = 10.4 Hz, 1H), 7.88 (t, *J* = 7.3 Hz, 1H), 8.08 (s, 1H), 12.23 (s, 1H); ^{13}C NMR (126 MHz, DMSO-d_6) δ (ppm): 24.5, 25.3, 33.2, 53.3, 65.2, 109.4, 113.7 (d, *J* = 24.5 Hz), 115.1, 121.5, 122.1 (d, *J* = 13.2 Hz), 127.3, 128.9–129.8 (m), 131.2, 139.2, 146.4, 158.2 (d, *J* = 250.8 Hz); MS (ESI): 392.1 $[M+H]^+$.

3-(*tert*-Butylamino)-2-(3,4-difluorophenyl)-1*H*-imidazo[1,2-*b*]pyrazole-7-carbonitrile (26)

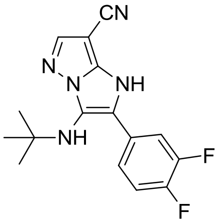

26

Pale yellow crystals; 93 mg (59%); m.p.: 241–243°C; TLC: R_f = 0.62 (hexane/EtOAc, v/v = 2:1); ^{1}H NMR (500 MHz, DMSO-d_6) δ (ppm): 1.07 (s, 9H), 4.61 (s, 1H), 7.50–7.59 (m, 1H), 7.81 (s, 1H), 8.01–8.13 (m, 2H), 12.48 (s; 1H); ^{13}C NMR (126 MHz, DMSO-d_6) δ (ppm): 30.0, 54.6, 65.4, 115.1, 115.3, 117.8 (d, J = 17.0 Hz), 123.0, 123.1, 123.2, 127.6, 138.5, 145.5, 148.6 (dd, J = 247.5, 11.5 Hz), 149.3 (dd, J = 244.5, 12.4 Hz); MS (ESI): 316.1 $[M+H]^+$.

2-(3,4-Difluorophenyl)-3-((2,4,4-trimethylpentan-2-yl)amino)-1*H*-imidazo[1,2-*b*]pyrazole-7-carbonitrile (27)

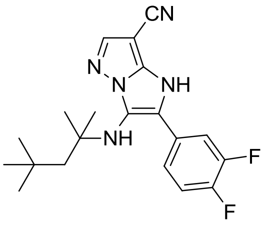

27

White crystals; 99 mg (53%); m.p.: 187–189°C; TLC: R_f = 0.72 (hexane/EtOAc, v/v = 2:1); ^{1}H NMR (500 MHz, DMSO-d_6) δ (ppm): 0.98 (s, 9H), 1.01 (s, 6H), 1.56 (s, 2H), 4.39 (s, 1H), 7.47–7.59 (m, 1H), 7.73 (s, 1H), 7.98–8.07 (m, 2H), 12.46 (s, 1H); ^{13}C NMR (126 MHz, DMSO-d_6) δ (ppm): 29.0, 31.3, 31.6, 55.5, 58.9, 65.3, 115.2, 115.8 (d, J = 19.2 Hz), 117.8 (d, J = 17.1 Hz), 123.0, 123.3, 123.7, 127.7, 138.4, 145.5, 148.7 (dd, J =246.4, 12.9 Hz), 149.3 (dd, J = 243.8, 11.4 Hz); MS (ESI): 372.2 $[M+H]^+$.

Methyl 2-((7-cyano-2-(3,4-difluorophenyl)-1*H*-imidazo[1,2-*b*]pyrazol-3-yl)amino)acetate (28)

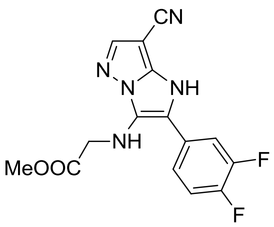

28

White crystals; 46 mg (28%); m.p.: 206–209°C; TLC: R_f = 0.38 (hexane/EtOAc, v/v = 2:1); ^{1}H NMR (500 MHz, DMSO-d_6) δ (ppm): 3.57 (s, 3H), 4.21 (d, *J* = 4.8 Hz, 2H), 5.88–5.95 (m, 1H), 7.54 (dd, *J* = 18.1, 8.9 Hz, 1H), 7.58–7.64 (m, 1H), 7.92–8.00 (m, 1H), 8.05 (s, 1H), 12.29 (s, 1H); ^{13}C NMR (126 MHz, DMSO-d_6) δ (ppm): 45.8, 51.6, 65.4, 114.5, 114.8 (d, *J* = 19.3 Hz), 115.1, 117.9 (d, *J* = 17.3 Hz), 122.5, 125.4, 127.3, 138.4, 146.3, 148.2 (dd, *J* = 246.6, 12.7 Hz), 149.6 (dd, *J* = 244.6, 12.6 Hz), 171.9; MS (ESI): 332.1 $[M+H]^+$.

3-(Cyclohexylamino)-2-(3,4-difluorophenyl)-1*H*-imidazo[1,2-*b*]pyrazole-7-carbonitrile (29)

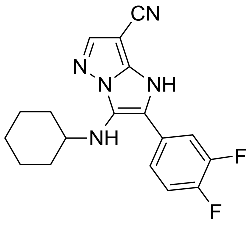

29

White crystals; 41 mg (24%); m.p.: 221–223°C; TLC: R_f = 0.64 (hexane/EtOAc, v/v = 2:1); ^{1}H NMR (500 MHz, DMSO-d_6) δ (ppm): 1.06–1.26 (m, 5H), 1.44–1.53 (m, 1H), 1.56–1.69 (m, 2H), 1.70–1.82 (m, 2H), 3.37 (s, 1H), 4.94 (d, *J* = 3.4 Hz), 7.51–7.60 (m, 1H), 7.69–7.76 (m, 1H), 7.92–8.01 (m, 1H), 8.06 (s, 1H), 12.36 (s, 1H); ^{13}C NMR (126 MHz, DMSO-d_6) δ (ppm): 24.2, 25.4, 33.1, 54.2, 65.4, 114.3 (d, *J* = 19.6 Hz), 115.1, 118.0 (d, *J* = 17.2 Hz), 118.8, 122.2 124.8, 127.5, 138.5, 146.0, 148.3 (dd, *J* = 247.0, 12.8 Hz), 149.5 (dd, *J* = 244.6, 12.5 Hz); MS (ESI): 342.1 $[M+H]^+$.

3-(*tert*-Butylamino)-2-(4-fluoro-3-(trifluoromethyl)phenyl)-1*H*-imidazo[1,2-*b*]pyrazole-7-carbonitrile (30)

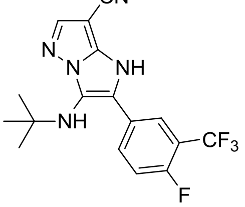

30

White crystals; 97 mg (53%); m.p.: 249–252°C; TLC: R_f = 0.59 (hexane/EtOAc, v/v = 2:1); ^{1}H NMR (500 MHz, DMSO-d_6) δ (ppm): 1.07 (s, 9H), 4.63 (s, 1H), 7.60 (t, *J* = 9.0 Hz, 1H), 8.04 (s, 1H), 8.33 (s, 1H), 8.51 (d, *J* = 4.4 Hz, 1H), 12.61 (s, 1H); ^{13}C NMR (126 MHz, DMSO-d_6) δ (ppm): 30.0, 54.6, 65.5, 115.1, 116.8 (qd, *J* = 32.3, 11.9 Hz), 117.6 (d, *J* = 20.8 Hz), 122.6 (q, *J* = 271.6 Hz), 122.7, 123.41, 124.6, 127.3, 132.2 (d, *J* = 7.9 Hz), 138.7, 145.6, 157.7 (d, *J* = 255.0 Hz); MS (ESI): 366.1 $[M+H]^+$.

2-(4-Fluoro-3-(trifluoromethyl)phenyl)-3-((2,4,4-trimethylpentan-2-yl)amino)-1*H*-imidazo[1,2-*b*]pyrazole-7-carbonitrile (31)

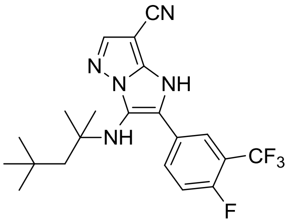

31

White crystals; 86 mg (41%); m.p.: 216–217°C; TLC: R_f = 0.69 (hexane/EtOAc, v/v = 2:1); ^{1}H NMR (500 MHz, DMSO-d_6) δ (ppm): 0.95 (s, 9H), 1.03 (s, 6H), 1.57 (s, 2H), 4.42 (s, 1H), 7.60–7.69 (m, 1H), 8.05 (s, 1H), 8.24 (s, 1H), 8.43 (s, 1H), 12.59 (s, 1H); ^{13}C NMR (126 MHz, DMSO-d_6) δ (ppm): 29.0, 31.3, 31.5, 55.6, 58.9, 65.4, 115.09, 116.3–117.3 (m), 117.6 (d, *J* = 20.5 Hz), 122.6 (q, *J* = 270.3 Hz), 122.9, 123.2, 125.3, 127.3, 132.8 (d, *J* = 7.4 Hz), 138.6, 145.5, 157.9 (d, *J* = 256.4 Hz); MS (ESI): 422.2 $[M+H]^+$.

Methyl 2-((7-cyano-2-(4-fluoro-3-(trifluoromethyl)phenyl)-1*H*-imidazo[1,2-*b*]pyrazol-3-yl)amino)acetate (32)

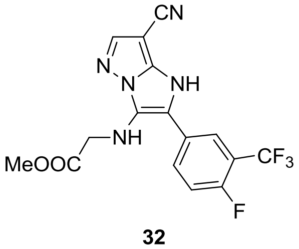

32

Greyish-white crystals; 63 mg (33%); m.p.: 211–213°C; TLC: R_f = 0.37 (hexane/EtOAc, v/v = 2:1); ^{1}H NMR (500 MHz, DMSO-d_6) δ (ppm): 3.57 (s, 3H), 4.22 (d, *J* = 3.0 Hz, 2H), 6.00 (s, 1H), 7.62 (t, *J* = 9.3 Hz, 1H), 8.07 (s, 1H), 8.19 (s, 1H), 8.27 (d, *J* = 4.9 Hz, 1H), 12.42 (s, 1H); ^{13}C NMR (126 MHz, DMSO-d_6) δ (ppm): 45.8, 51.6, 65.4, 114.6, 115.0, 117.1 (dd, *J* = 31.7, 12.3 Hz), 117.7 (d, *J* = 20.6 Hz), 122.6 (q, *J* = 272.3 Hz), 124.4 (d, *J* = 2.6 Hz), 125.5, 127.0, 132.0 (d, *J* = 8.0 Hz), 138.6, 146.3, 157.5 (d, *J* = 254.3 Hz), 171.9; MS (ESI): 382.0 $[M+H]^+$.

3-(Cyclohexylamino)-2-(4-fluoro-3-(trifluoromethyl)phenyl)-1*H*-imidazo[1,2-*b*]pyrazole-7-carbonitrile (33)

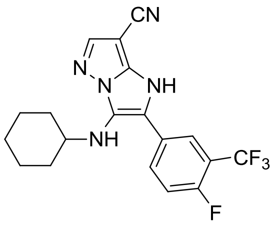

33

White crystals; 90 mg (46%); m.p.: 197–200°C; TLC: R_f = 0.63 (hexane/EtOAc, v/v = 2:1); ^{1}H NMR (500 MHz, DMSO-d_6) δ (ppm): 1.02–1.29 (m, 6H), 1.41–1.52 (m, 1H), 1.56–1.66 (m, 2H), 1.70–1.80 (m, 2H), 4.96 (s, 1H), 7.60 (t, *J* = 8.8 Hz, 1H), 8.06 (s, 1H), 8.21–8.29 (m, 1H), 8.37 (d, *J* = 4.5 Hz, 1H), 12.51 (s, 1H); ^{13}C NMR (126 MHz, DMSO-d_6) δ (ppm): 24.1, 25.4, 33.06, 54.3, 65.5, 115.1, 117.0 (qd, *J* = 31.9, 11.7 Hz), 117.7 (d, *J* = 20.9 Hz), 118.8, 122.6 (q, *J* = 272.1 Hz), 123.8 (d, *J* = 3.2 Hz), 125.0, 127.1, 131.4 (d, *J* = 7.8 Hz), 138.6, 146.0, 157.5 (d, *J* = 254.6 Hz); MS (ESI): 392.0 $[M+H]^+$.

3-(*tert*-Butylamino)-2-(2,4,6-trimethoxyphenyl)-1*H*-imidazo[1,2-*b*]pyrazole-7-carbonitrile (34)

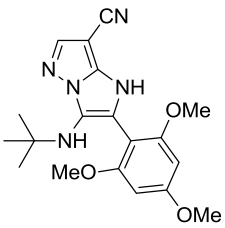

34

Pale yellow crystals; 130 mg (70%); m.p.: 203–205°C; TLC: R_f = 0.14 (hexane/EtOAc, v/v = 2:1); ^{1}H NMR (500 MHz, DMSO-d_6) δ (ppm): 0.93 (s, 9H), 3.78 (s, 6H), 3.84 (s, 3H), 6.35 (s, 2H), 7.93 (s, 1H), 11.83 (s, 1H); ^{13}C NMR (126 MHz, DMSO-d_6) δ (ppm): 29.5, 54.0, 55.5, 56.0, 64.7, 91.1, 99.3, 115.6, 117.2, 124.0, 138.1, 144.5, 159.1, 162.2; MS (ESI): 370.1 $[M+H]^+$.

2-(2,4,6-Trimethoxyphenyl)-3-((2,4,4-trimethylpentan-2-yl)amino)-1*H*-imidazo[1,2-*b*]pyrazole-7-carbonitrile (35)

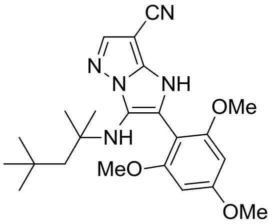

35

Pale yellow crystals; 177 mg (83%); m.p.: 203–205°C; TLC: R_f = 0.28 (hexane/EtOAc, v/v = 2:1); ^{1}H NMR (500 MHz, DMSO-d_6) δ (ppm): 0.90 (s, 9H), 0.92 (s, 6H), 1.33 (s, 2H), 3.17 (s, 1H), 3.76 (s, 6H), 3.83 (s, 3H), 6.35 (s, 2H), 7.93 (s, 1H), 11.83 (s, 1H); ^{13}C NMR (126 MHz, DMSO-d_6) δ (ppm): 28.8, 31.2, 31.5, 55.1, 55.5, 56.0, 57.8, 64.7, 91.0, 99.4, 115.6, 117.2, 123.9, 138.2, 144.5, 159.0, 162.3; MS (ESI): 426.2 $[M+H]^+$.

Methyl 2-((7-cyano-2-(2,4,6-trimethoxyphenyl)-1*H*-imidazo[1,2-*b*]pyrazol-3-yl)amino)-acetate (36)

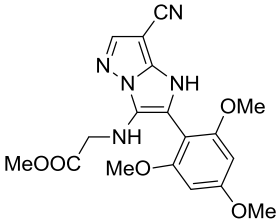

36

Pale yellow crystals; 93 mg (48%); m.p.: 170–173°C; TLC: R_f = 0.08 (hexane/EtOAc, v/v = 2:1); ^{1}H NMR (500 MHz, DMSO-d_6) δ (ppm): 3.46 (s, 3H), 3.74 (s, 6H), 3.79–3.91 (m, 5H), 4.91 (s, 1H), 6.31 (s, 2H), 7.97 (s, 1H), 11.54 (s, 1H); ^{13}C NMR (126 MHz, DMSO-d_6) δ (ppm): 45.8, 51.4, 55.5, 55.8, 64.7, 90.8, 98.7, 106.5, 115.6, 125.9, 137.3, 145.2, 159.6, 162.1, 171.3; MS (ESI): 386.1 $[M+H]^+$.

3-(Cyclohexylamino)-2-(2,4,6-trimethoxyphenyl)-1*H*-imidazo[1,2-*b*]pyrazole-7-carbonitrile (37)

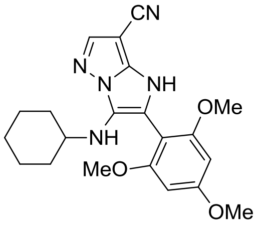

37

Pale yellow crystals; 95 mg (48%); m.p.: 153–155°C; TLC: R_f = 0.21 (hexane/EtOAc, v/v = 2:1); ^{1}H NMR (500 MHz, DMSO-d_6) δ (ppm): 0.94–1.10 (m, 5H), 1.40–1.46 (m, 1H), 1.50–1.57 (m, 2H), 1.64–1.71 (m, 2H), 3.06–3.14 (m, 1H), 3.73 (s, 6H), 3.83 (s, 3H), 3.88 (d, J = 9.9 Hz, 1H), 6.31 (s, 2H), 7.94 (s, 1H), 11.56 (s, 1H); ^{13}C NMR (126 MHz, DMSO-d_6) δ (ppm): 24.5, 25.4, 33.1, 53.4, 55.5, 55.8, 64.7, 90.9, 99.0, 109.6, 115.7, 125.7, 137.6, 144.9, 159.6, 162.1; MS (ESI): 396.2 $[M+H]^+$.

(*E*)-3-(*tert*-Butylamino)-2-(1-phenylprop-1-en-2-yl)-1*H*-imidazo[1,2-*b*]pyrazole-7-carbonitrile (38)

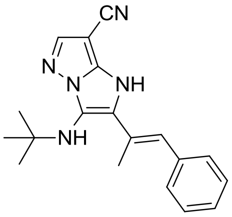

38

White crystals; 97 mg (61%); m.p.: 183–185°C; TLC: R_f = 0.67 (hexane/EtOAc, v/v = 2:1); ^{1}H NMR (500 MHz, DMSO-d_6) δ (ppm): 1.12 (s, 9H), 2.28 (s, 3H), 4.38 (s, 1H), 7.07 (s, 1H), 7.24–7.29 (m, 1H), 7.35–7.43 (m, 4H), 7.99 (s, 1H), 12.08 (s, 1H); ^{13}C NMR (126 MHz, DMSO-d_6) δ (ppm): 16.3, 30.0, 54.6, 65.1, 115.4, 122.8, 126.8, 126.9, 127.6, 128.4, 128.7, 128.9, 136.9, 137.8, 145.1; MS (ESI): 320.1 $[M+H]^+$.

(*E*)-2-(1-Phenylprop-1-en-2-yl)-3-((2,4,4-trimethylpentan-2-yl)amino)-1*H*-imidazo[1,2-*b*]pyrazole-7-carbonitrile (39)

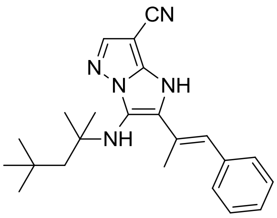

39

White crystals; 126 mg (67%); m.p.: 156–158°C; TLC: R_f = 0.74 (hexane/EtOAc, v/v = 2:1); ^{1}H NMR (500 MHz, DMSO-d_6) δ (ppm): 0.99 (s, 9H), 1.11 (s, 6H), 1.60 (s, 2H), 2.26 (s, 3H), 4.15 (s, 1H), 7.03 (s, 1H), 7.27 (t, J = 6.9 Hz, 1H), 7.35–7.42 (m, 4H), 7.99 (s, 1H), 12.07 (s, 1H); ^{13}C NMR (126 MHz, DMSO-d_6) δ (ppm): 16.7, 28.9, 31.3, 31.7, 55.3, 58.6, 65.0, 115.4, 122.5, 126.7, 126.9, 127.8, 128.4, 128.9, 129.3, 136.9, 137.7, 145.1; MS (ESI): 376.2 $[M+H]^+$.

(*E*)-Methyl 2-((7-cyano-2-(1-phenylprop-1-en-2-yl)-1*H*-imidazo[1,2-*b*]pyrazol-3-yl)-amino)acetate (40)

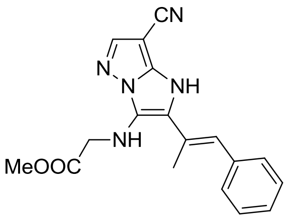

40

Pale yellow crystals; 44 mg (26%); m.p.: 145–146°C; TLC: R_f = 0.44 (hexane/EtOAc, v/v = 2:1); ^{1}H NMR (500 MHz, DMSO-d_6) δ (ppm): 2.27 (s, 3H), 3.58 (s, 3H), 4.20 (d, *J* = 6.3 Hz, 2H), 5.67 (t, *J* = 6.1 Hz, 1H), 6.86 (s, 1H), 7.25 (t, *J* = 6.3 Hz, 1H), 7.34–7.42 (m, 4H), 8.01 (s, 1H), 11.91 (s, 1H); ^{13}C NMR (126 MHz, DMSO-d_6) δ (ppm): 16.2, 46.0, 51.6, 65.0, 115.3, 118.4, 125.5, 126.0, 126.5, 126.6, 128.3, 129.0, 137.1, 137.9, 146.0, 171.7; MS (ESI): 336.1 $[M+H]^+$.

(*E*)-3-(Cyclohexylamino)-2-(1-phenylprop-1-en-2-yl)-1*H*-imidazo[1,2-*b*]pyrazole-7-carbonitrile (41)

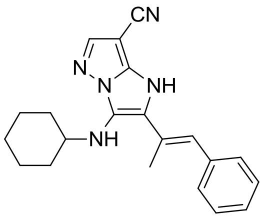

41

White crystals; 109 mg (63%); m.p.: 152–153°C; TLC: R_f = 0.71 (hexane/EtOAc, v/v = 2:1); ^{1}H NMR (500 MHz, DMSO-d_6) δ (ppm): 1.04–1.30 (m, 6H), 1.45–1.55 (m, 1H), 1.60–1.70 (m, 2H), 1.73–1.82 (m, 2H), 2.28 (s, 3H), 4.66 (d, *J* = 6.0 Hz, 1H), 6.96 (s, 1H), 7.25 (t, *J* = 6.3 Hz, 1H), 7.33–7.42 (m, 4H), 8.01 (s, 1H), 11.95 (s, 1H); ^{13}C NMR (126 MHz, DMSO-d_6) δ (ppm): 16.1, 24.4, 25.5, 33.2, 54.5, 65.1, 115.4, 122.7, 124.8, 126.5, 126.6, 126.7, 128.4, 129.0, 137.1, 137.9, 145.6; MS (ESI): 346.1 $[M+H]^+$.

2-(*tert*-Butyl)-3-(*tert*-butylamino)-1*H*-imidazo[1,2-*b*]pyrazole-7-carbonitrile (42)

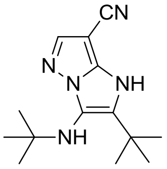

42

White crystals; 65 mg (50%); m.p.: 212–214°C; TLC: R_f = 0.65 (hexane/EtOAc, v/v = 2:1); ^{1}H NMR (500 MHz, DMSO-d_6) δ (ppm): 1.18 (s, 9H), 1.36 (s, 9H), 3.80 (s, 1H), 7.90 (s, 1H),

11.56 (s, 1H); ^{13}C NMR (126 MHz, DMSO-d_6) δ (ppm): 30.0, 30.7, 32.1, 52.4, 64.9, 115.7, 120.9, 133.4, 137.2, 144.3; MS (ESI): 260.2 $[M+H]^+$.

2-(*tert*-Butyl)-3-((2,4,4-trimethylpentan-2-yl)amino)-1*H*-imidazo[1,2-*b*]pyrazole-7-carbonitrile (43)

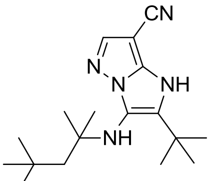

43

White crystals; 71 mg (45%); m.p.: 168–170°C; TLC: R_f = 0.78 (hexane/EtOAc, v/v = 2:1); ^{1}H NMR (500 MHz, DMSO-d_6) δ (ppm): 1.00 (s, 9H), 1.20 (s, 6H), 1.36 (s, 9H), 1.67 (s, 2H), 3.63 (s, 1H), 7.90 (s, 1H), 11.54 (s, 1H); ^{13}C NMR (126 MHz, DMSO-d_6) δ (ppm): 29.5, 30.1, 31.4, 31.8, 32.1, 56.1, 56.4, 64.8, 115.7, 120.7, 133.3, 137.2, 144.2; MS (ESI): 316.2 $[M+H]^+$.

Methyl 2-((2-(*tert*-butyl)-7-cyano-1*H*-imidazo[1,2-*b*]pyrazol-3-yl)amino)acetate (44)

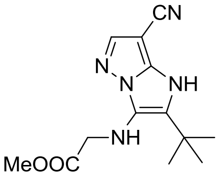

44

White crystals; 65 mg (47%); m.p.: 169–171°C; TLC: R_f = 0.42 (hexane/EtOAc, v/v = 2:1); ^{1}H NMR (500 MHz, DMSO-d_6) δ (ppm): 1.36 (s, 9H), 3.59 (s, 3H), 3.98 (d, J = 5.7 Hz, 2H), 4.83 (t, J = 5.4 Hz, 1H), 7.93 (s, 1H), 11.56 (s, 1H); ^{13}C NMR (126 MHz, DMSO-d_6) δ (ppm): 29.6, 31.9, 47.1, 51.4, 65.1, 115.5, 122.2, 128.0, 136.8, 145.0, 171.6; MS (ESI): 276.1 $[M+H]^+$.

2-(*tert*-Butyl)-3-(cyclohexylamino)-1*H*-imidazo[1,2-*b*]pyrazole-7-carbonitrile (45)

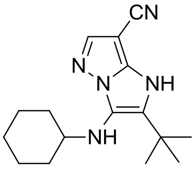

45

White crystals; 57 mg (40%); m.p.: 189–191°C; TLC: R_f = 0.73 (hexane/EtOAc, v/v = 2:1); ^{1}H NMR (500 MHz, DMSO-d_6) δ (ppm): 1.07–1.20 (m, 5H), 1.34 (s, 9H), 1.48–1.56 (m, 1H), 1.61–1.79 (m, 4H), 4.04 (s, 1H), 7.90 (s, 1H), 11.52 (s, 1H); ^{13}C NMR (126 MHz, DMSO-d_6)

δ (ppm): 24.5, 25.6, 29.8, 31.9, 33.3, 54.5, 64.9, 115.7, 121.9, 130.6, 136.9, 144.6; MS (ESI): 286.2 $[M+H]^+$.

Ethyl 3-(*tert*-butylamino)-2-(*p*-tolyl)-1*H*-imidazo[1,2-*b*]pyrazole-7-carboxylate (46)

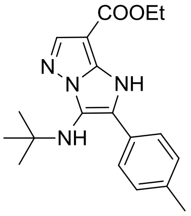

46

Pale yellow crystals; 92 mg (54%); m.p.: 132–134°C; TLC: 0.52 (hexane/EtOAc, v/v = 2:1); ^{1}H NMR (500 MHz, DMSO-d_6) δ (ppm): 1.02 (s, 9H), 1.27 (t, J = 6.8 Hz, 3H), 2.32 (s, 3H), 4.21 (q, J = 6.7 Hz, 2H), 4.27 (s, 1H), 7.22 (d, J = 7.5 Hz, 2H), 7.83–7.88 (m, 3H), 11.80 (s, 1H); ^{13}C NMR (126 MHz, DMSO-d_6) δ (ppm): 14.7, 20.9, 30.1, 54.6, 58.7, 90.1, 122.0, 124.8, 127.2, 127.5, 128.7, 136.7, 137.6, 143.3, 162.3; MS (ESI): 341.0 $[M+H]^+$.

Ethyl 2-phenyl-3-((2,4,4-trimethylpentan-2-yl)amino)-1*H*-imidazo[1,2-*b*]pyrazole-7-carboxylate (47)

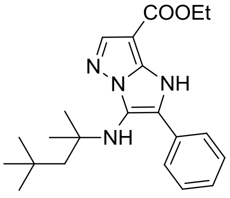

47

White crystals; 107 mg (56%); m.p.: 162–164°C; TLC: 0.63 (hexane/EtOAc, v/v = 2:1); ^{1}H NMR (500 MHz, DMSO-d_6) δ (ppm): 0.95 (s, 15H), 1.28 (s, 3H), 1.50 (s, 2H), 4.10 (s, 1H), 4.16–4.26 (m, 2H), 7.32 (s, 1H), 7.42 (s, 2H), 7.87 (s, 3H), 11.87 (s, 1H); ^{13}C NMR (126 MHz, DMSO-d_6) δ (ppm): 14.7, 29.0, 31.3, 31.6, 55.4, 58.7, 61.7, 90.0, 122.1, 124.9, 127.5, 127.9, 128.1, 130.4, 137.7, 143.4, 162.2; MS (ESI): 383.3 $[M+H]^+$.

3-(*tert*-Butylamino)-6-methyl-2-(*p*-tolyl)-1*H*-imidazo[1,2-*b*]pyrazole-7-carbonitrile (48)

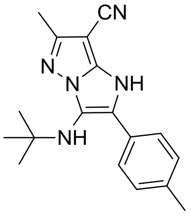

48

White crystals; 122 mg (79%); m.p.: 231–233°C; TLC: 0.52 (hexane/EtOAc, v/v = 2:1); ^{1}H NMR (500 MHz, DMSO-d_6) δ (ppm): 1.04 (s, 9H), 2.31 (s, 3H), 2.32 (s, 3H), 4.35 (s, 1H), 7.23 (d, *J* = 7.3 Hz, 2H), 7.83 (d, *J* = 7.4 Hz, 2H), 12.14 (s, 1H); ^{13}C NMR (126 MHz, DMSO-d_6) δ (ppm): 13.5, 20.8, 30.1, 54.8, 65.2, 115.6, 122.1, 123.7, 126.0, 127.4, 129.0, 136.8, 138.5, 154.4; MS (ESI): 307.9 $[M+H]^+$.

Methyl 2-((7-cyano-2-(4-fluorophenyl)-6-methyl-1*H*-imidazo[1,2-*b*]pyrazol-3-yl)amino)-acetate (49)

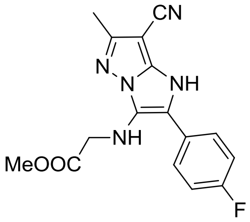

49

White crystals; 93 mg (57%); m.p.: 219–221°C; TLC: 0.21 (hexane/EtOAc, v/v = 2:1); ^{1}H NMR (500 MHz, DMSO-d_6) δ (ppm): 2.32 (s, 3H), 3.56 (s, 3H), 4.15 (d, *J* = 6.0 Hz, 2H), 5.69 (t, *J* = 5.7 Hz, 1H), 7.29 (t, *J* = 8.5 Hz, 2H), 7.83 (dd, *J* = 6.9 Hz, *J* = 5.5 Hz, 2H), 12.09 (s, 1H); ^{13}C NMR (126 MHz, DMSO-d_6) δ (ppm): 13.4, 46.0, 51.6, 61.7, 65.3, 114.6, 115.5, 115.6 (d, *J* = 21.4 Hz), 124.5, 126.4, 127.7 (d, *J* = 7.1 Hz), 138.6, 155.4, 161.0 (d, *J* = 244.5 Hz), 171.8; MS (ESI): 327.9 $[M+H]^+$.

Ethyl 3-(*tert*-butylamino)-6-methyl-2-(*p*-tolyl)-1*H*-imidazo[1,2-*b*]pyrazole-7-carboxylate (50)

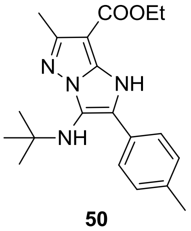

50

White crystals;131 mg (74%); m.p.: 108–110°C; TLC: 0.54 (hexane/EtOAc, v/v = 2:1); ^{1}H NMR (500 MHz, DMSO-d_6) δ (ppm): 1.01 (s, 9H), 1.28 (s, 3H), 2.31 (s, 3H), 2.40 (s, 3H), 4.21 (s, 2H), 4.35 (s, 1H), 7.22 (s, 2H), 7.82 (s, 2H), 11.45 (s, 1H); ^{13}C NMR (126 MHz, DMSO-d_6) δ (ppm): 14.8, 20.8, 30.1, 54.7, 58.4, 88.1, 121.7, 123.6, 127.1, 127.6, 128.7, 136.5, 138.7, 153.0, 162.7; MS (ESI): 355.0 $[M+H]^+$.

(E)-Ethyl 6-methyl-2-(1-phenylprop-1-en-2-yl)-3-((2,4,4-trimethylpentan-2-yl)amino)-1*H*-imidazo[1,2-*b*]pyrazole-7-carboxylate (51)

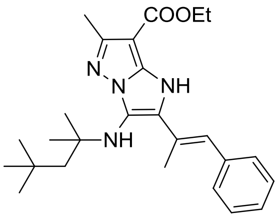

51

White crystals; 169 mg (59%); m.p.:118–119°C; TLC: 0.67 (hexane/EtOAc, v/v = 2:1); ^{1}H NMR (500 MHz, DMSO-d_6) δ (ppm): 0.98 (s, 9H), 1.10 (s, 6H), 1.27 (s, 3H), 1.58 (s, 2H), 2.26 (s, 3H), 2.39 (s, 3H), 3.97 (s, 1H), 4.20 (s, 2H), 6.92 (s, 1H), 7.26 (s, 1H), 7.38 (s, 4H), 11.28 (s, 1H); ^{13}C NMR (126 MHz, DMSO-d_6) δ (ppm): 14.8, 17.3, 28.9, 31.3, 31.6, 55.3, 58.4, 58.6, 87.8, 121.7, 126.5, 126.7, 127.0, 128.3, 128.9, 129.7, 137.2, 138.4, 152.9, 162.7; MS (ESI): 437.3 $[M+H]^+$.

2. ^{1}H,^{13}C-HSQC, ^{1}H,^{13}C-HMBC, ^{1}H,^{1}H-COSY and ^{1}H,^{1}H-NOESY NMR spectra of compound 6

2.1. Spectra of compound **6** obtained by GBB-3CR (applying 20 mol% $HClO_4$ in EtOH)

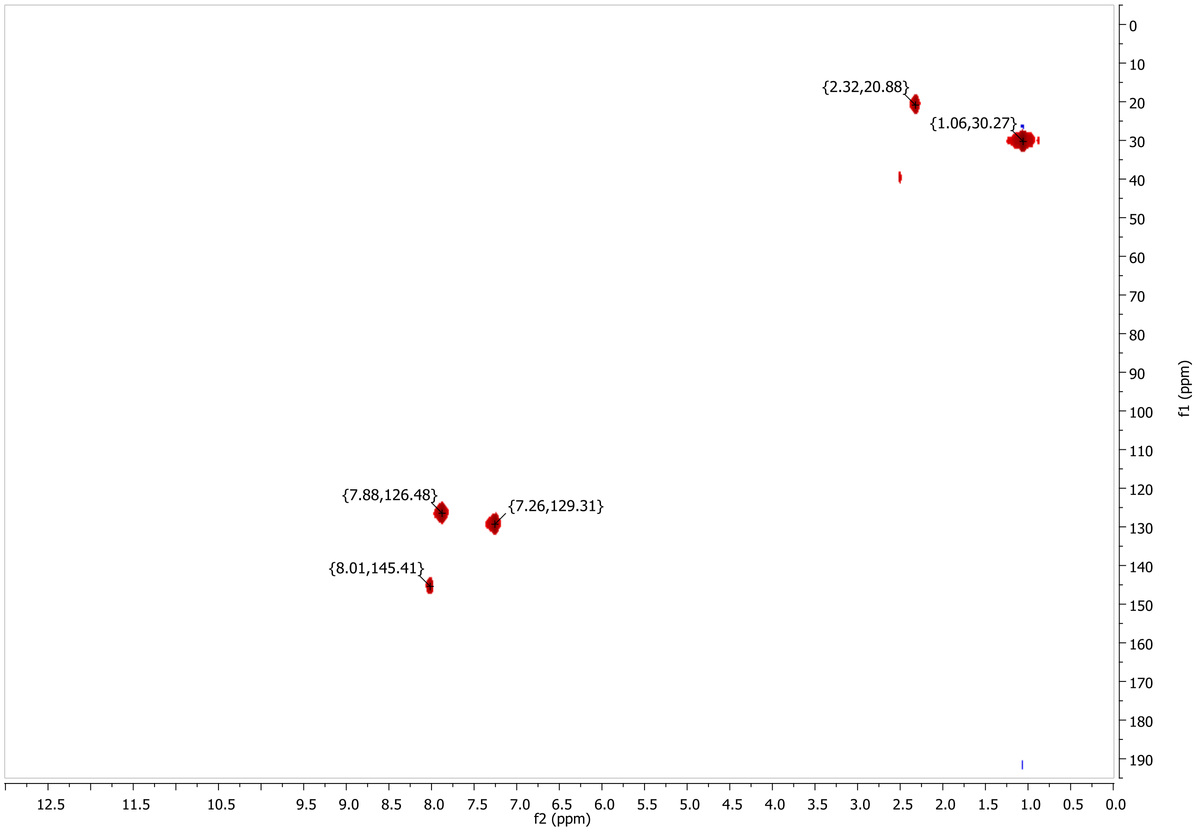

Figure S1. The HSQC spectrum of compound **6** in DMSO-d_6.

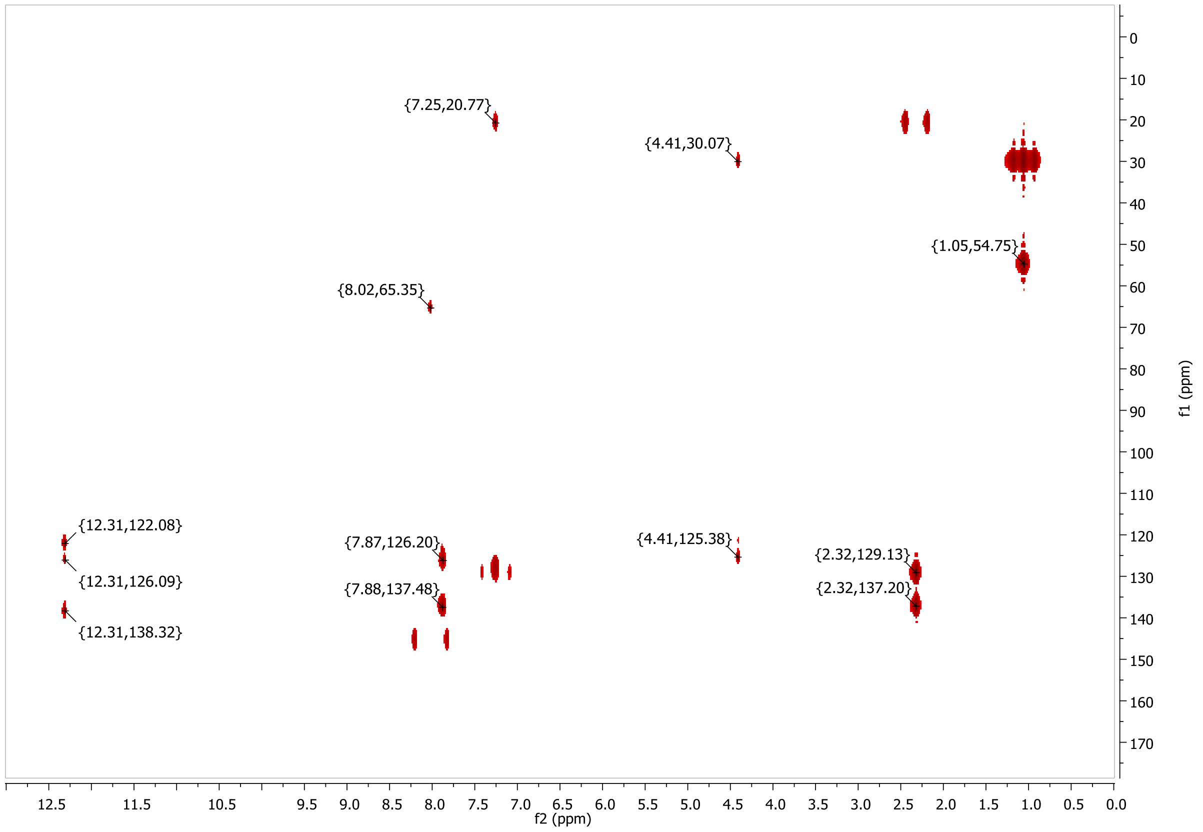

Figure S2. The HMBC spectrum of compound **6** in DMSO-d_6.

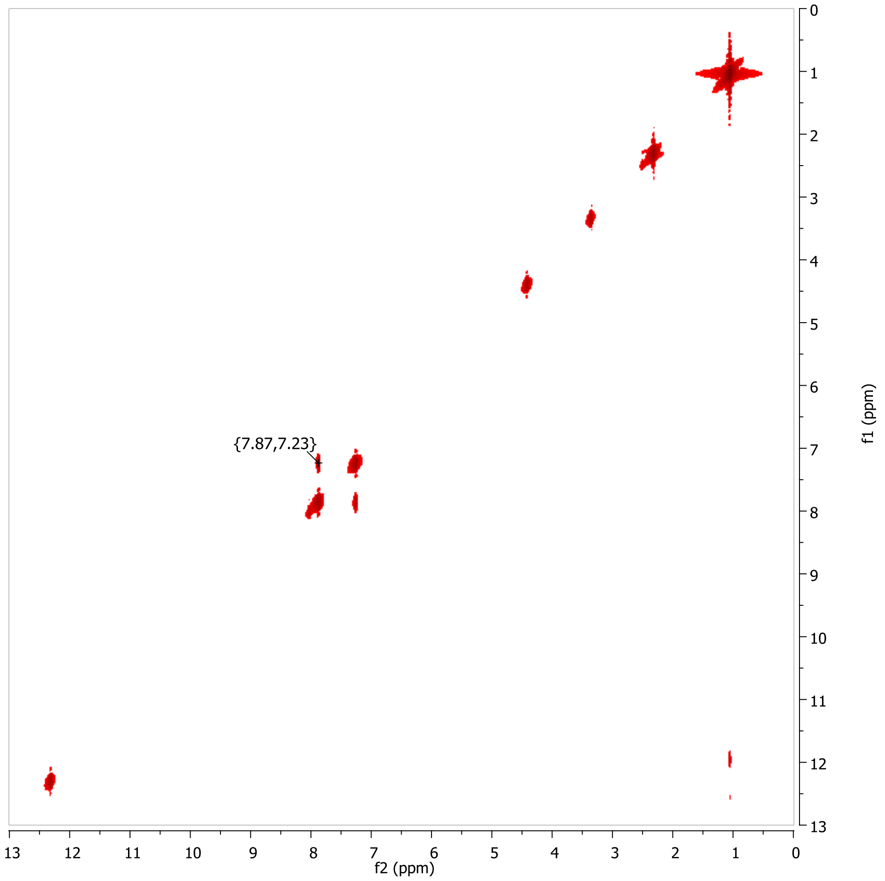

Figure S3. The COSY spectrum of compound **6** in DMSO-d_6.

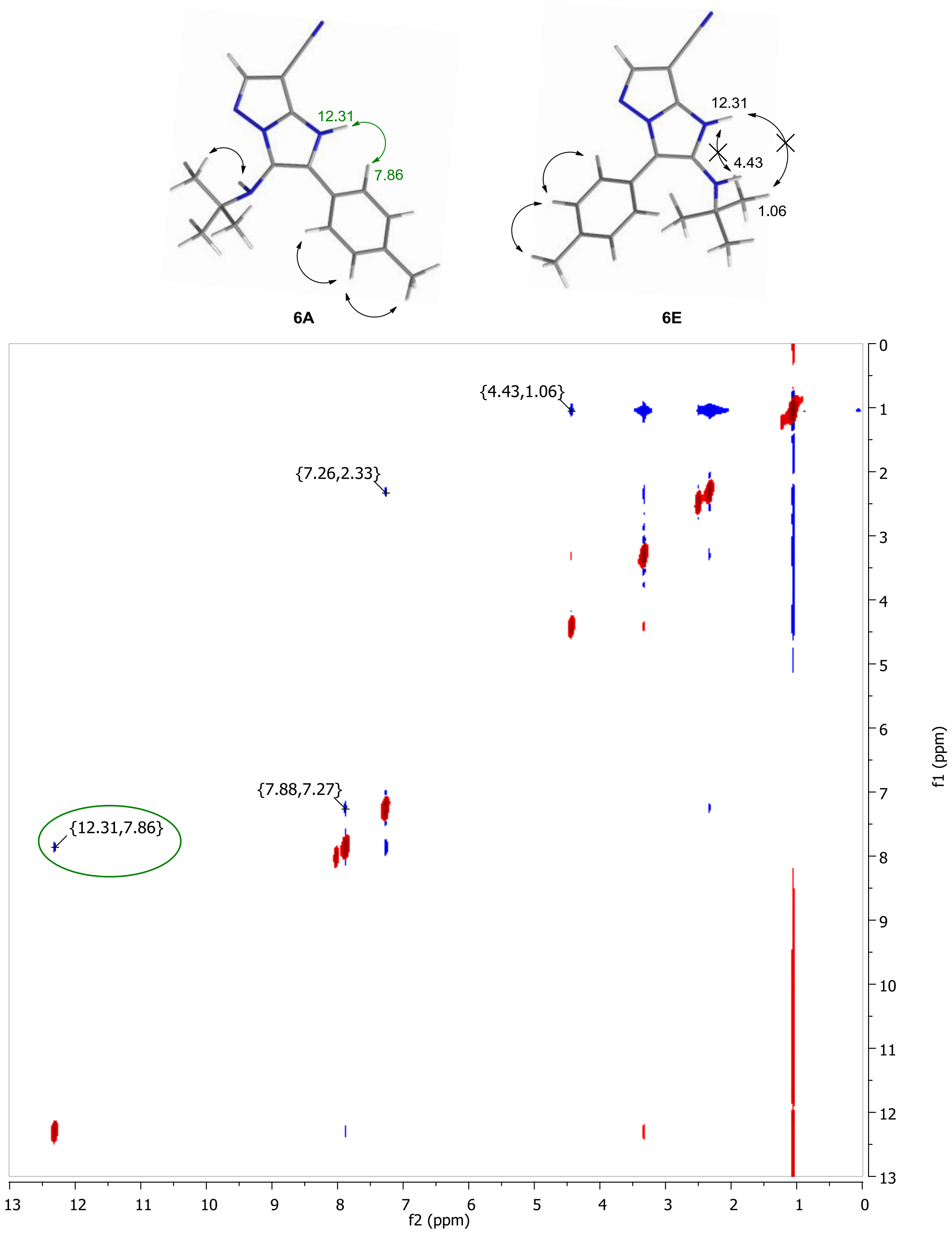

Figure S4. The NOESY spectrum of compound **6** in DMSO-d_6.

2.2. Spectra of compound **6** obtained by the optimized sequential one-pot method (applying 20 mol% TFA in EtOH/water mixture)

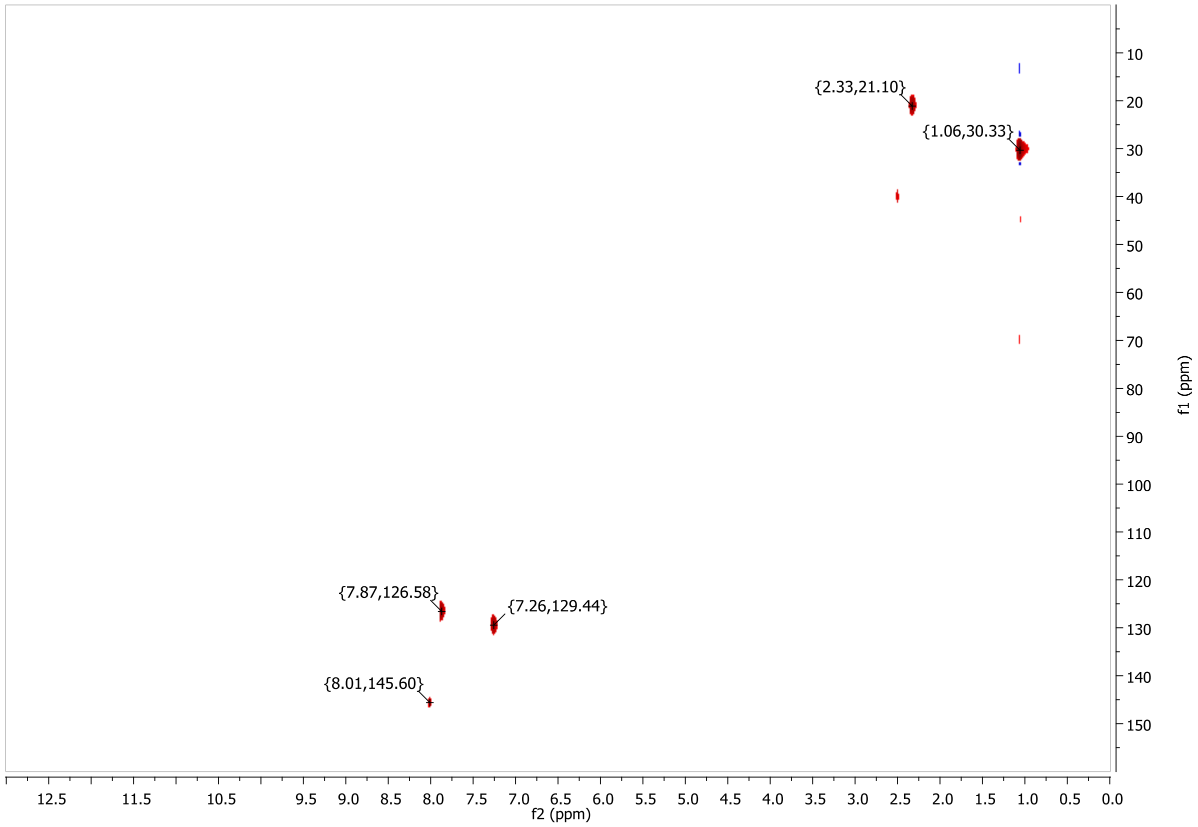

Figure S5. The HSQC spectrum of compound **6** in DMSO-d_6.

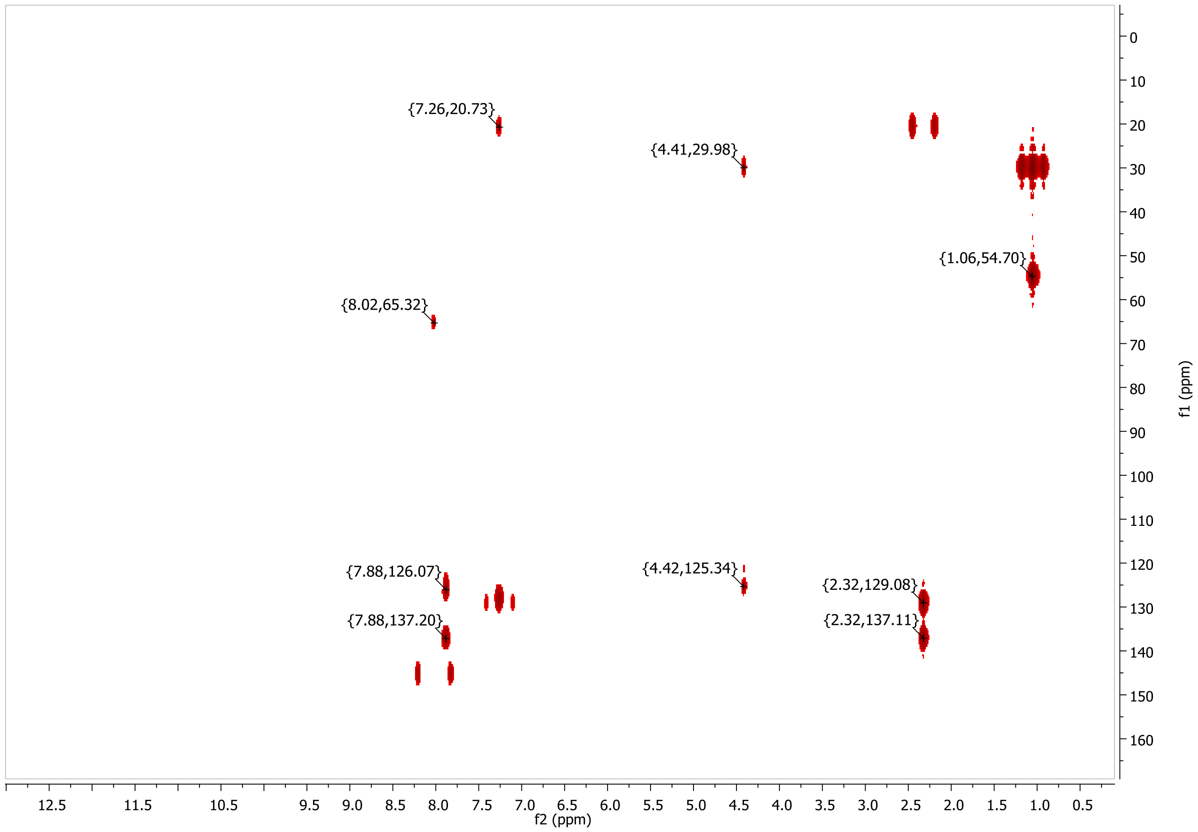

Figure S6. The HMBC spectrum of compound **6** in DMSO-d_6.

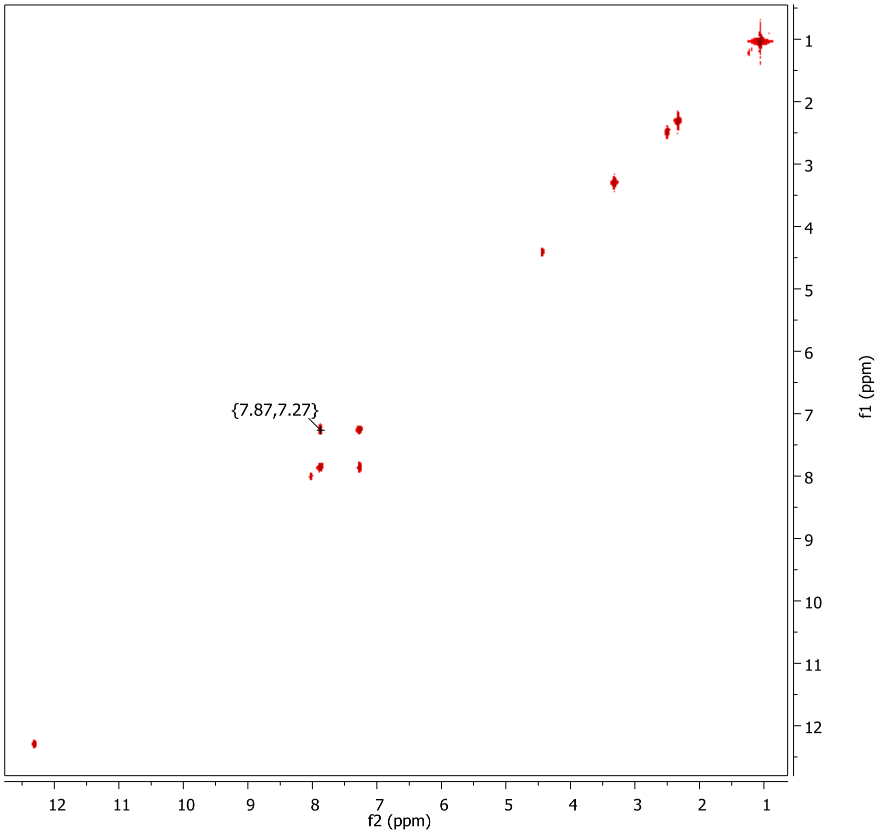

Figure S7. The COSY spectrum of compound **6** in DMSO-d_6.

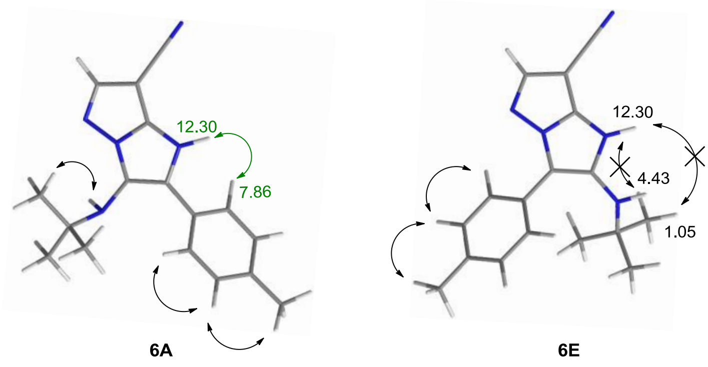

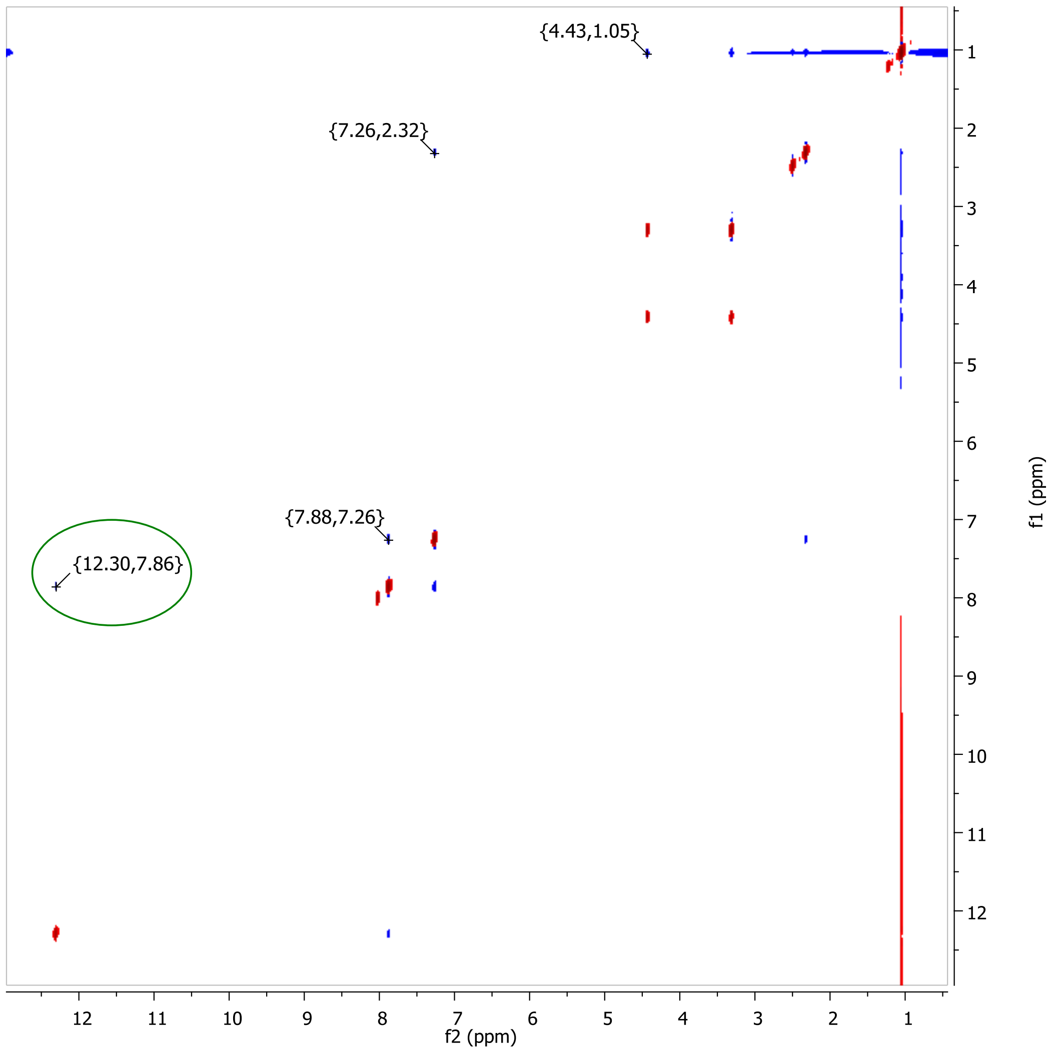

Figure S8. The NOESY spectrum of compound **6** in DMSO-d_6.

3. ^{1}H and ^{13}C NMR spectra for all compounds

3-(*tert*-Butylamino)-2-(*p*-tolyl)-1*H*-imidazo[1,2-*b*]pyrazole-7-carbonitrile (6)

^{1}H NMR (DMSO-d_6, 500 MHz)

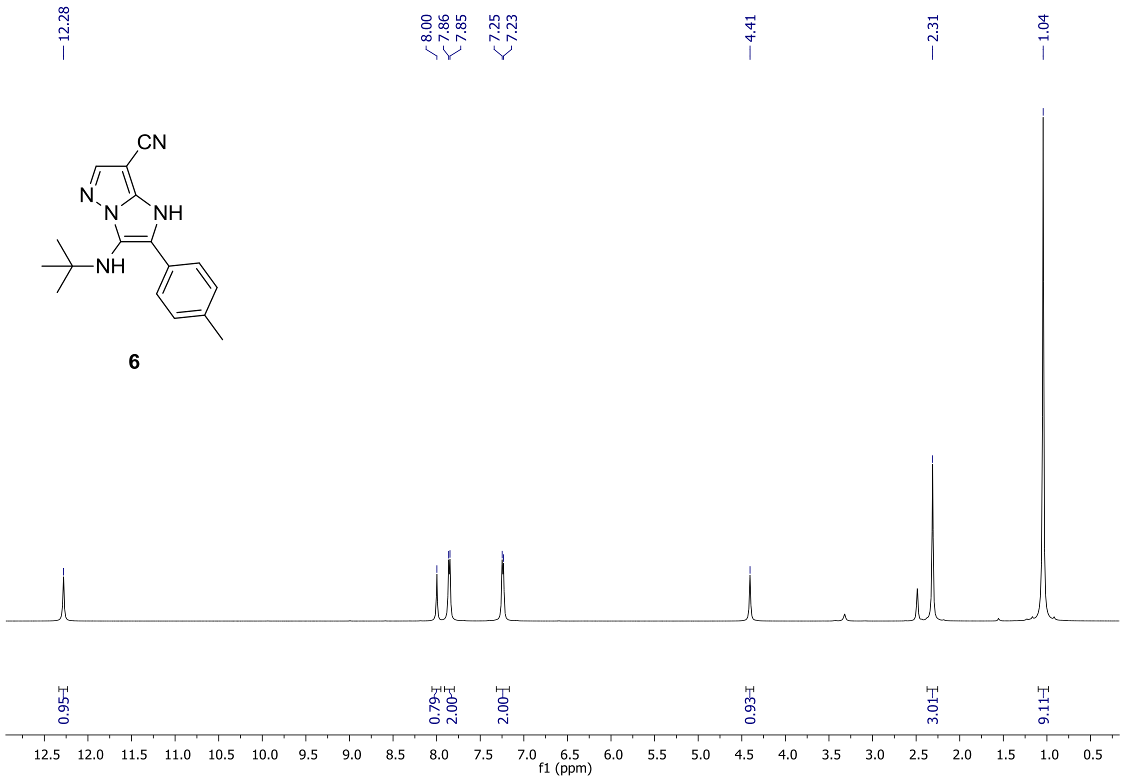

^{13}C NMR (DMSO-d_6, 125 MHz)

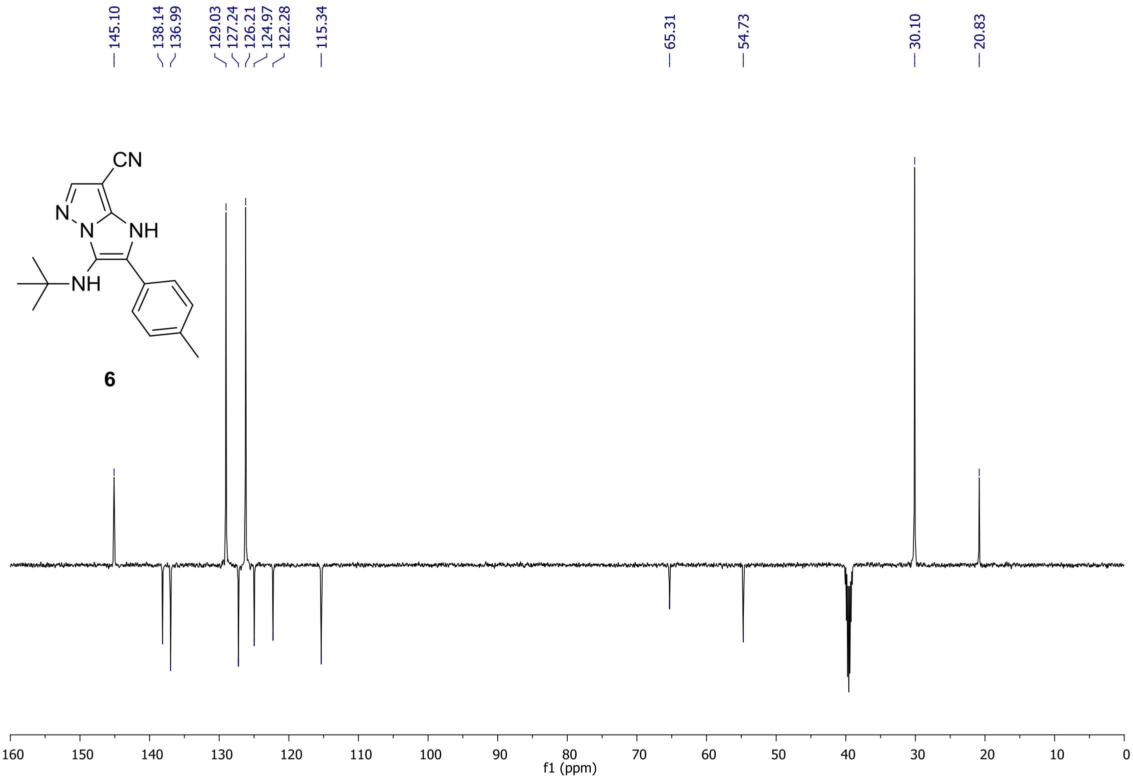

2-(*p*-Tolyl)-3-((2,4,4-trimethylpentan-2-yl)amino)-1*H*-imidazo[1,2-*b*]pyrazole-7-carbonitrile (7)

^{1}H NMR (DMSO-d_6, 500 MHz)

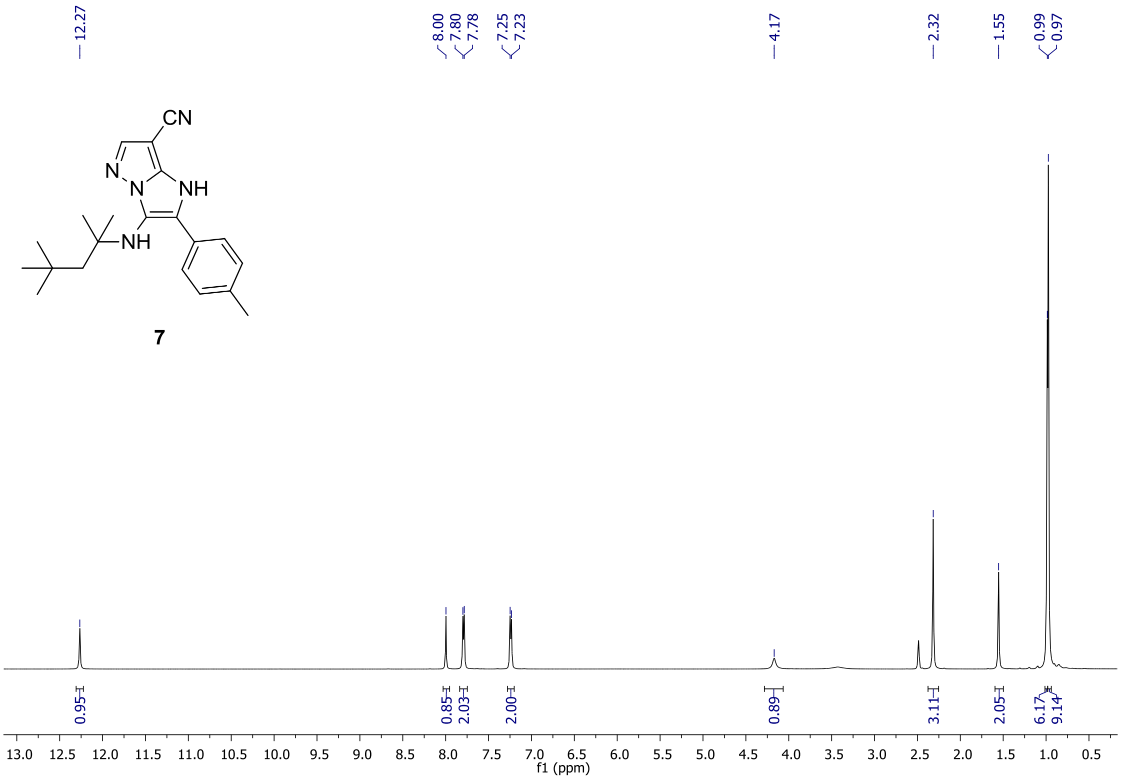

^{13}C NMR (DMSO-d_6, 125 MHz)

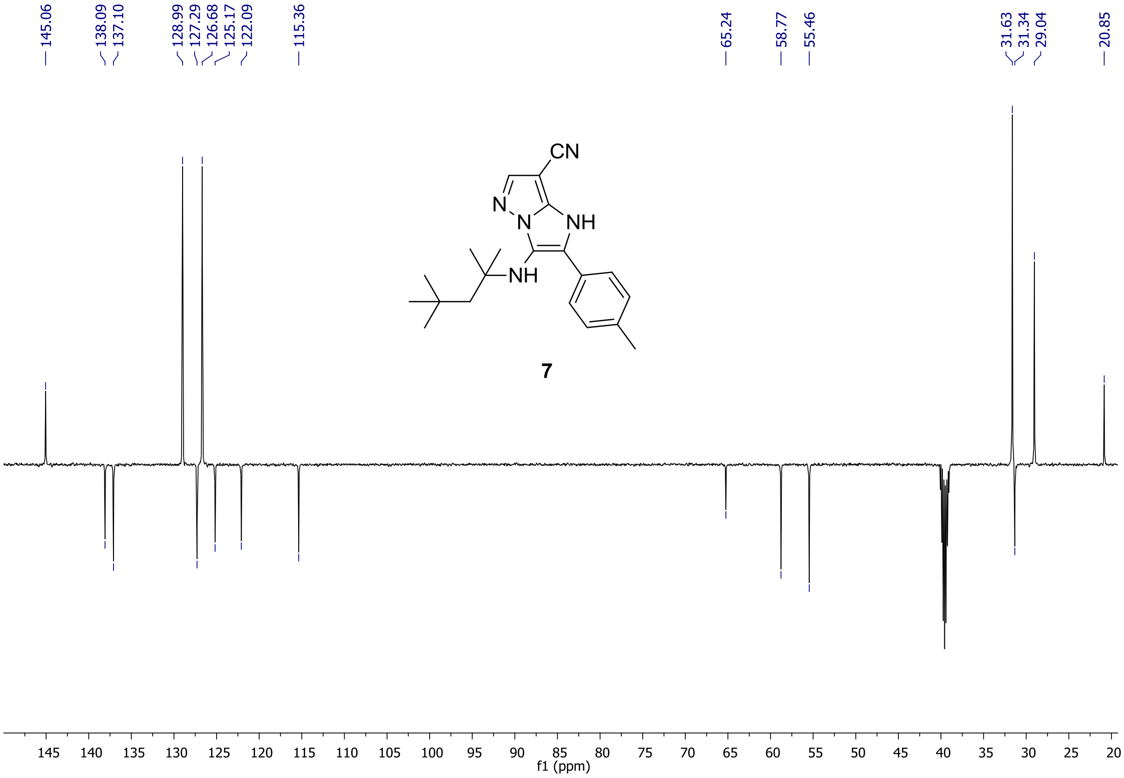

Methyl 2-((7-cyano-2-(*p*-tolyl)-1*H*-imidazo[1,2-*b*]pyrazole-3-yl)amino)acetate (8)

^{1}H NMR (DMSO-d_6, 500 MHz)

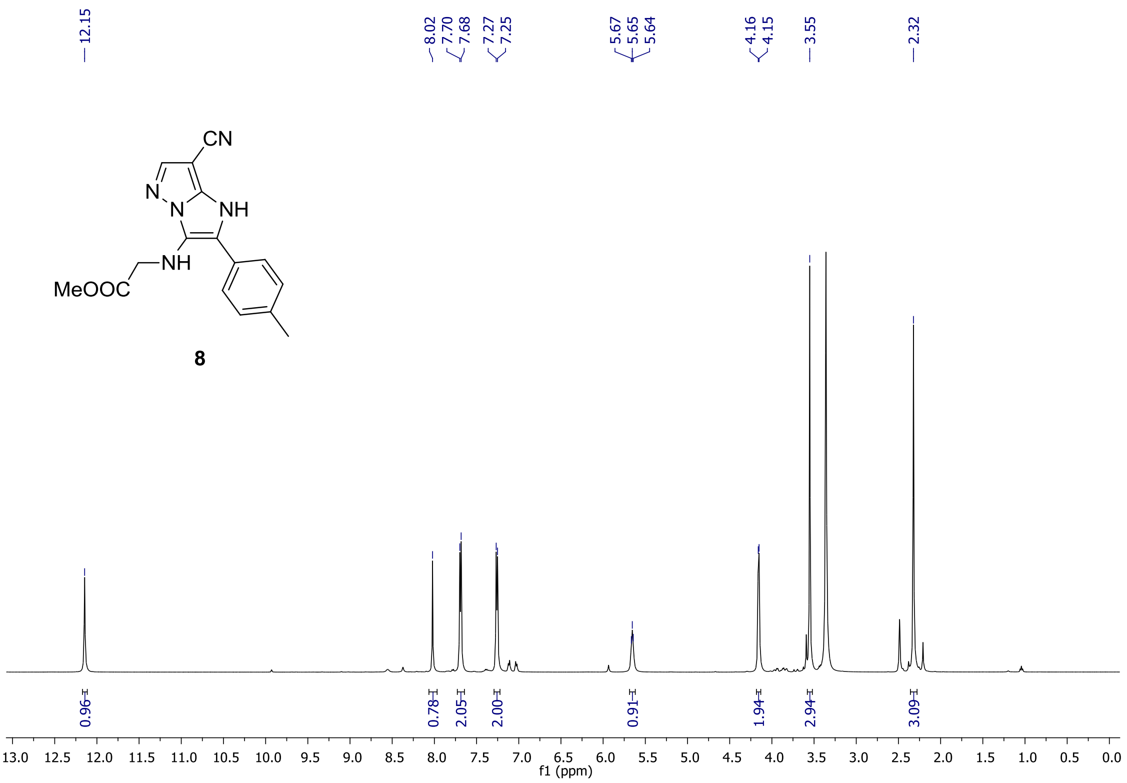

^{13}C NMR (DMSO-d_6, 125 MHz)

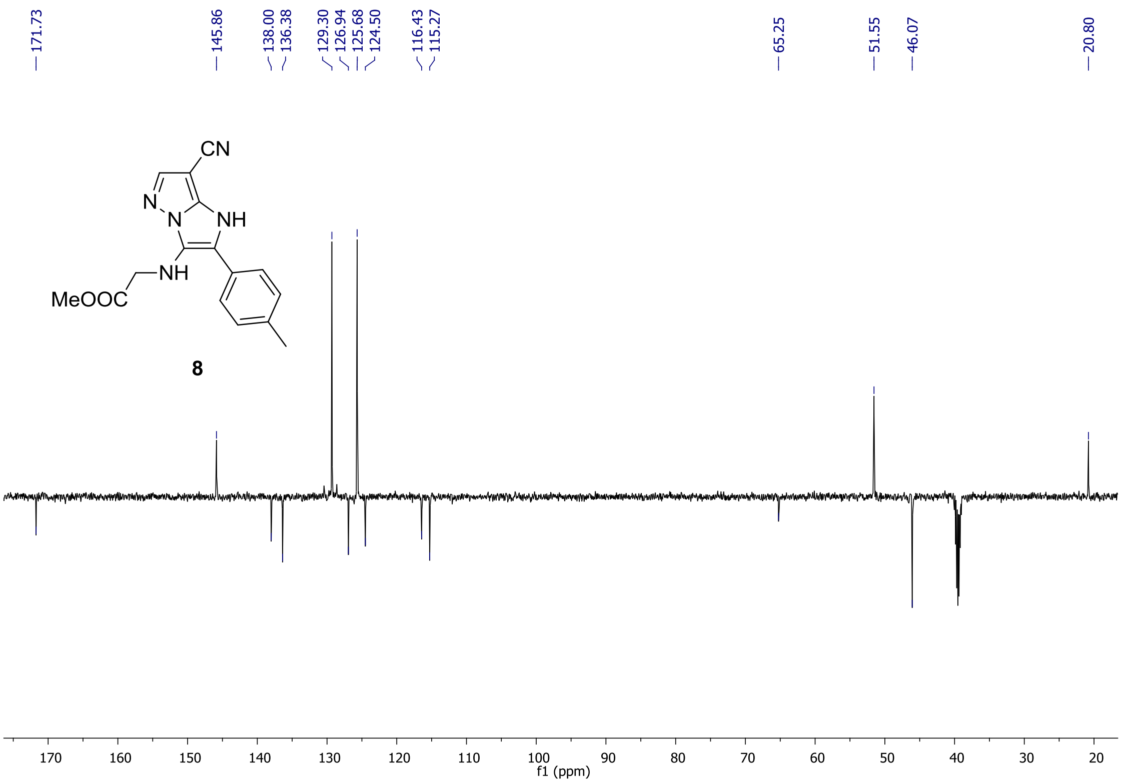

3-(Cyclohexylamino)-2-(*p*-tolyl)-1*H*-imidazo[1,2-*b*]pyrazole-7-carbonitrile (9)

^{1}H NMR (DMSO-d_6, 500 MHz)

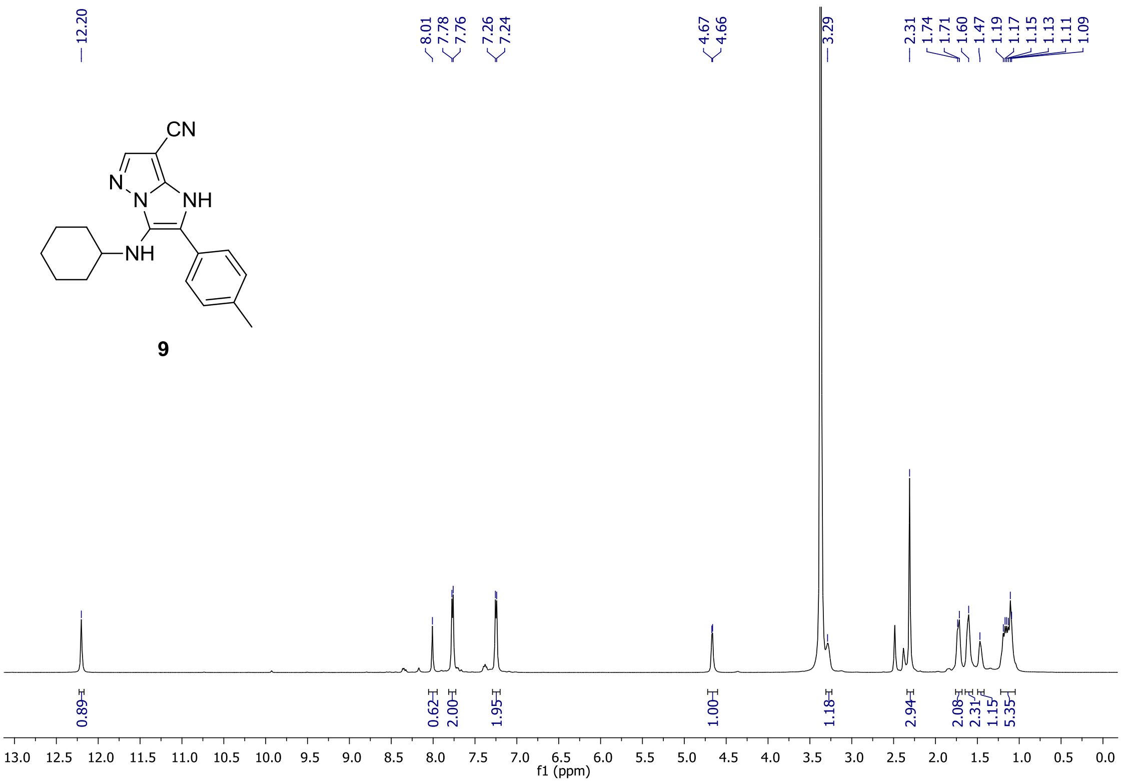

^{13}C NMR (DMSO-d_6, 125 MHz)

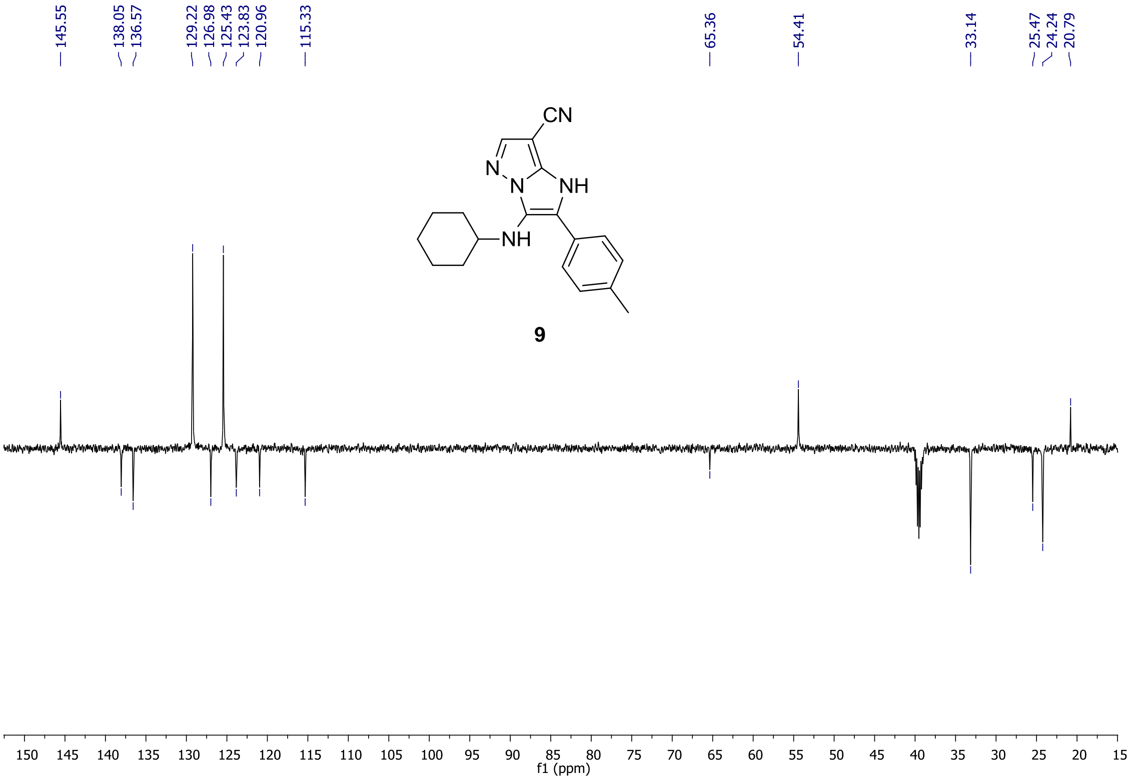

3-(*tert*-Butylamino)-2-phenyl-1*H*-imidazo[1,2-*b*]pyrazole-7-carbonitrile (10)

^{1}H NMR (DMSO-d_6, 500 MHz)

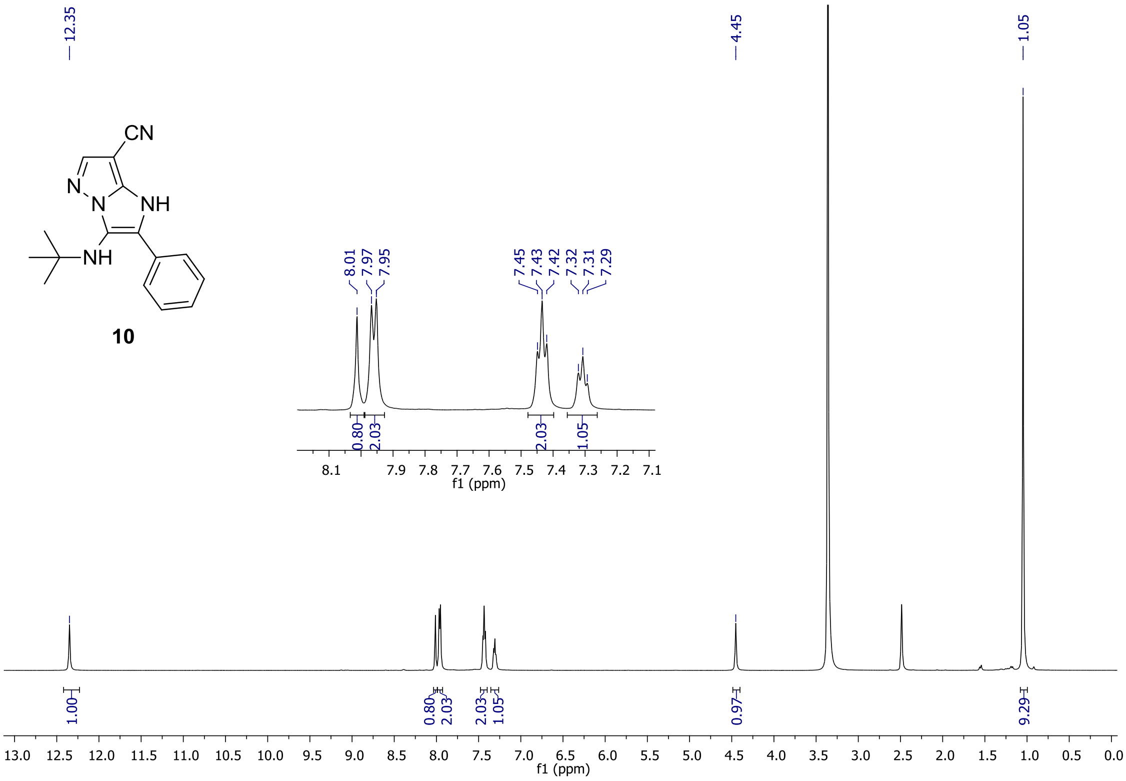

^{13}C NMR (DMSO-d_6, 125 MHz)

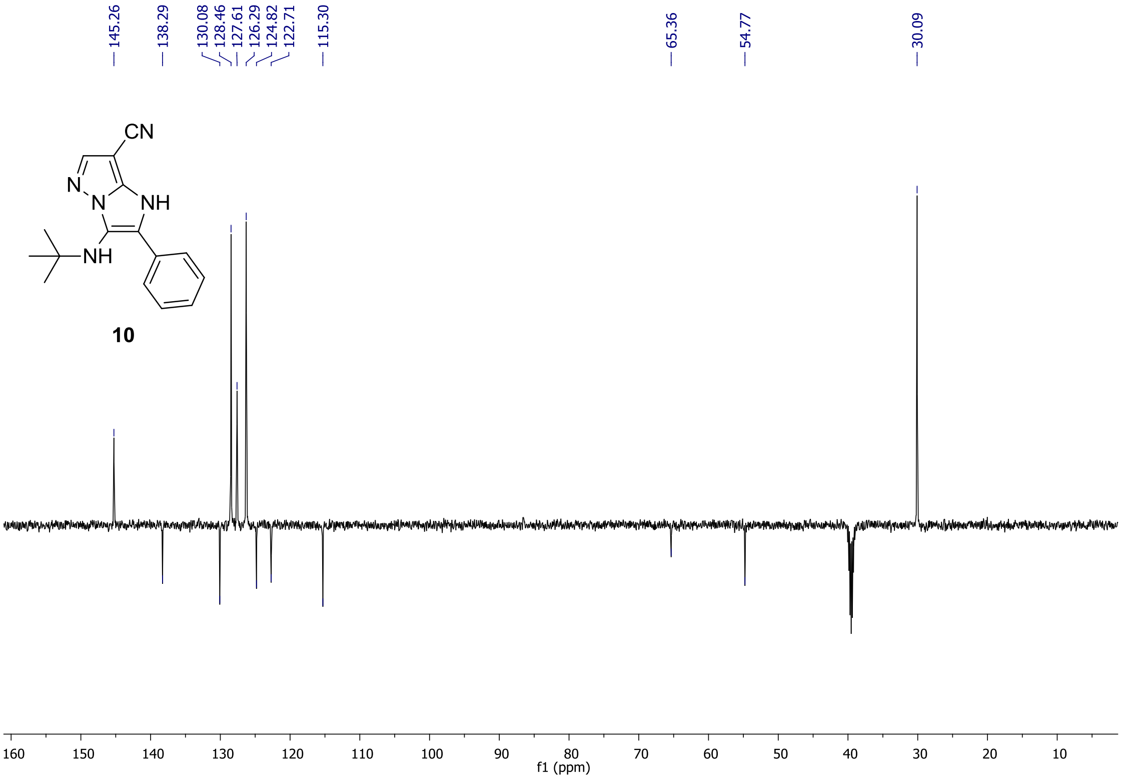

2-Phenyl-3-((2,4,4-trimethylpentan-2-yl)amino)-1*H*-imidazo[1,2-*b*]pyrazole-7-carbonitrile (11)

^{1}H NMR (DMSO-d_6, 500 MHz)

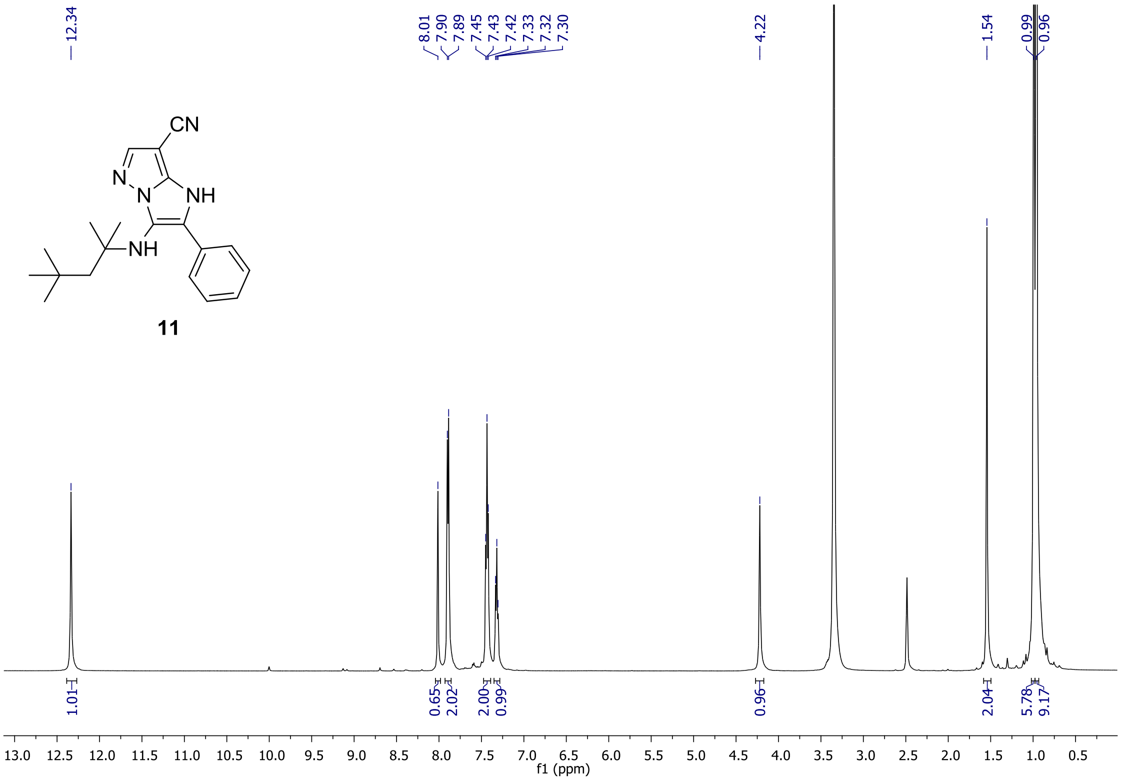

^{13}C NMR (DMSO-d_6, 125 MHz)

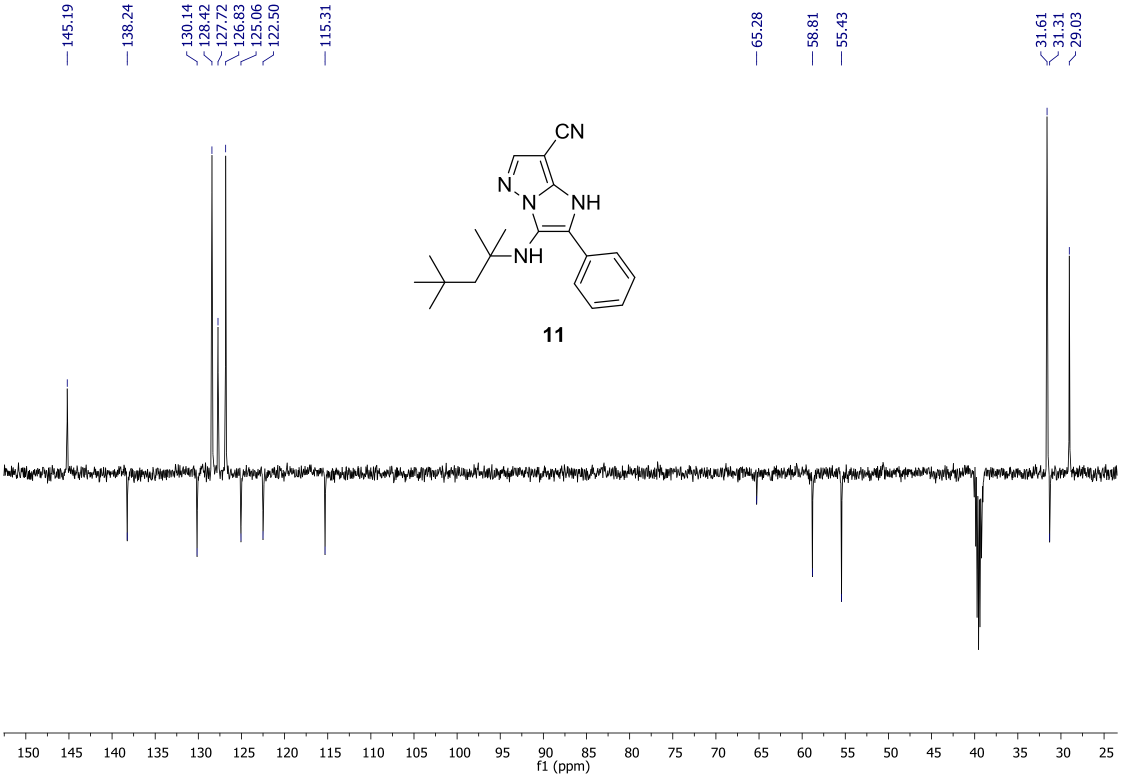

Methyl 2-((7-cyano-2-phenyl-1*H*-imidazo[1,2-*b*]pyrazole-3-yl)amino)acetate (12)

^{1}H NMR (DMSO-d_6, 500 MHz)

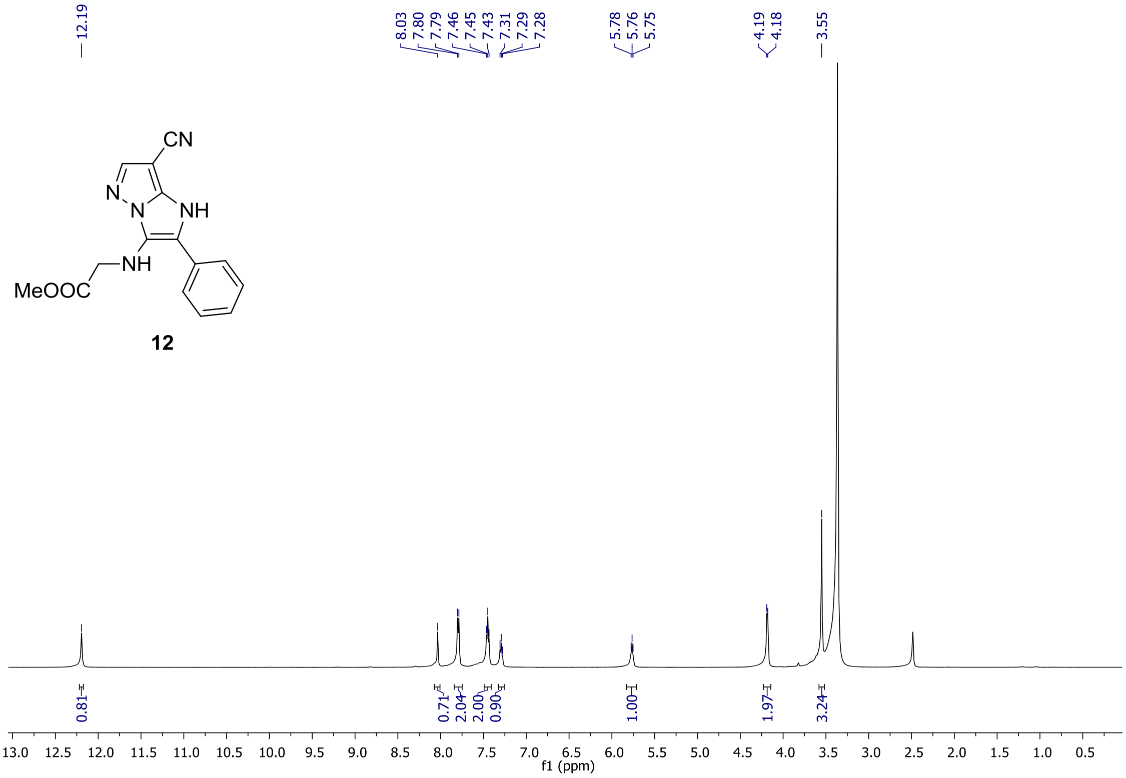

^{13}C NMR (DMSO-d_6, 125 MHz)

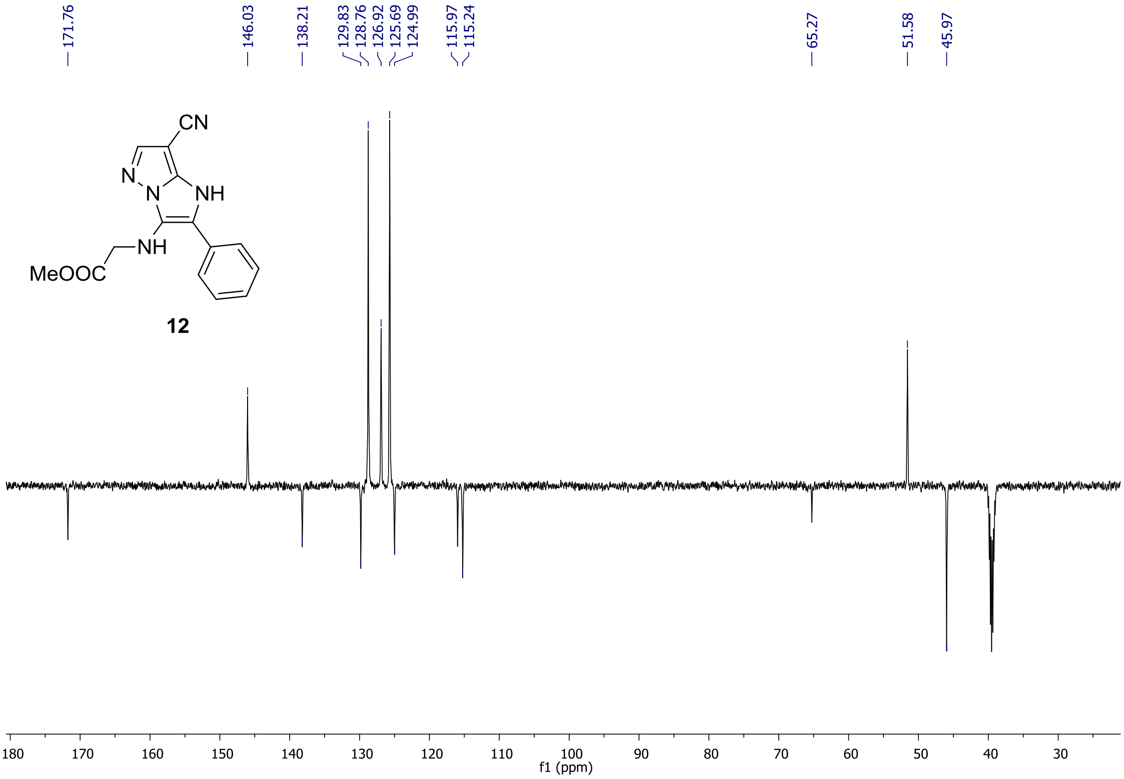

3-(Cyclohexylamino)-2-phenyl-1*H*-imidazo[1,2-*b*]pyrazole-7-carbonitrile (13)

^{1}H NMR (DMSO-d_6, 500 MHz)

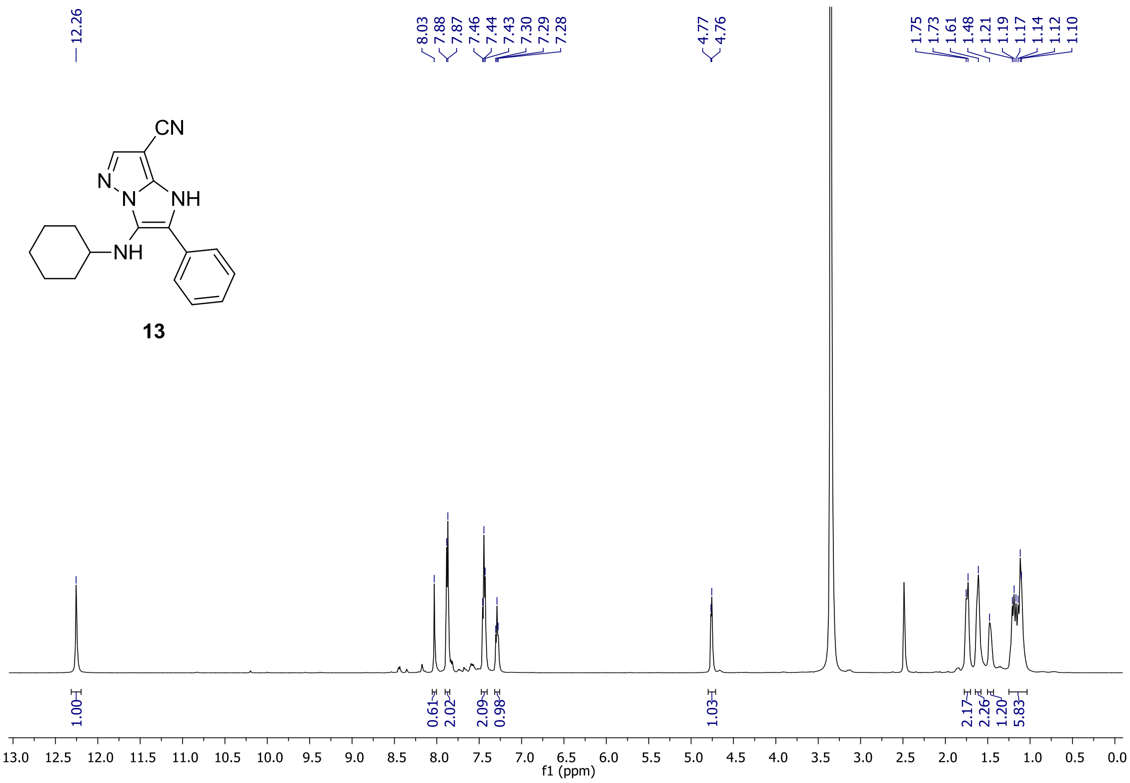

^{13}C NMR (DMSO-d_6, 125 MHz)

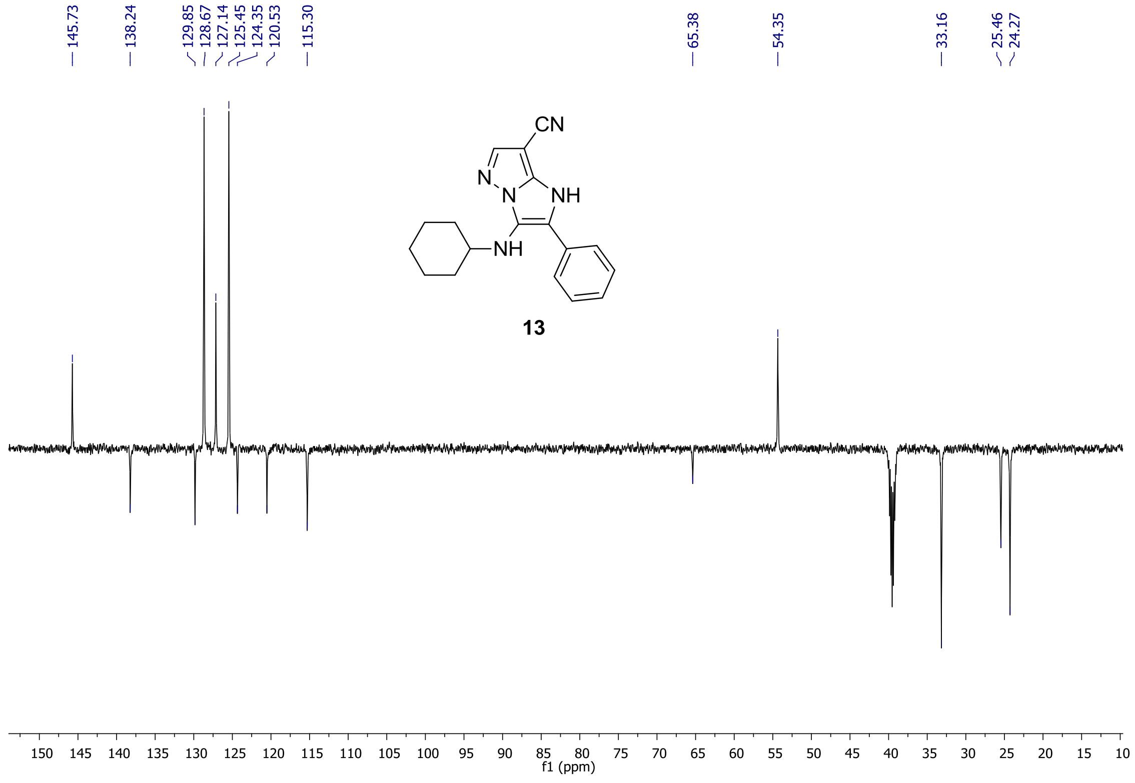

3-(*tert*-Butylamino)-2-(4-fluorophenyl)-1*H*-imidazo[1,2-*b*]pyrazole-7-carbonitrile (14)

^{1}H NMR (DMSO-d_6, 500 MHz)

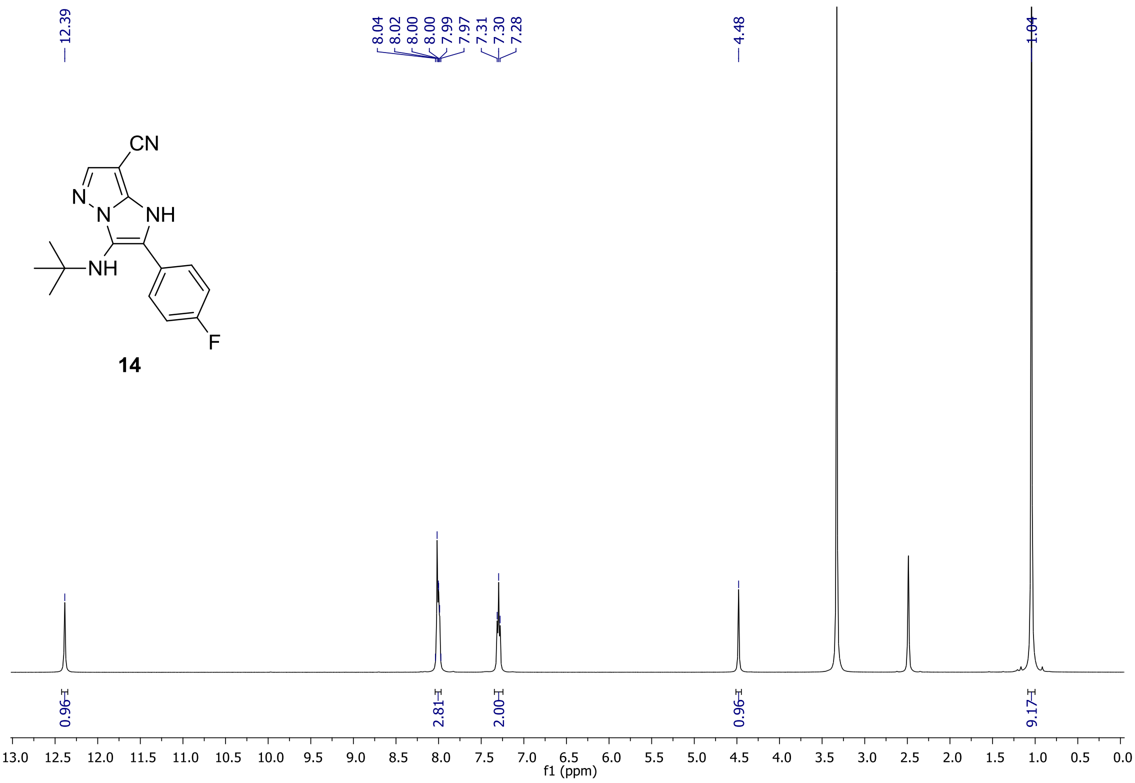

^{13}C NMR (DMSO-d_6, 125 MHz)

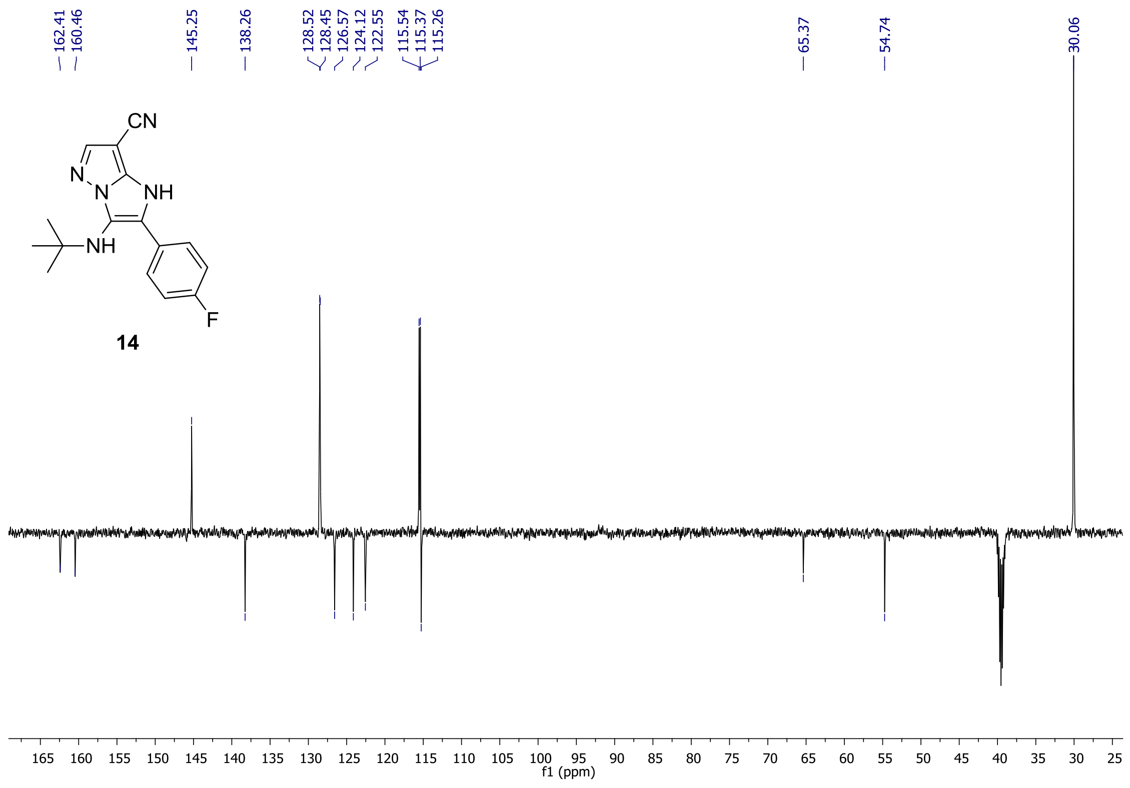

2-(4-Fluorophenyl)-3-((2,4,4-trimethylpentan-2-yl)amino)-1*H*-imidazo[1,2-*b*]pyrazole-7-carbonitrile (15)

^{1}H NMR (DMSO-d_6, 500 MHz)

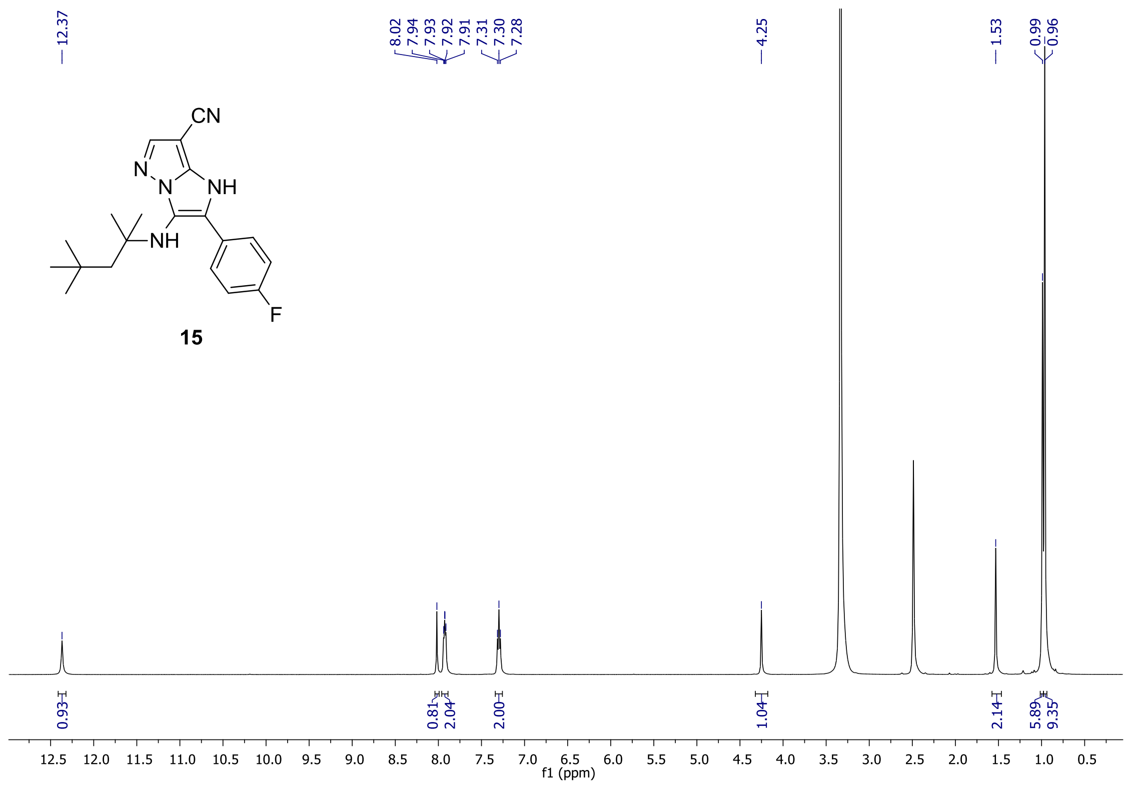

^{13}C NMR (DMSO-d_6, 125 MHz)

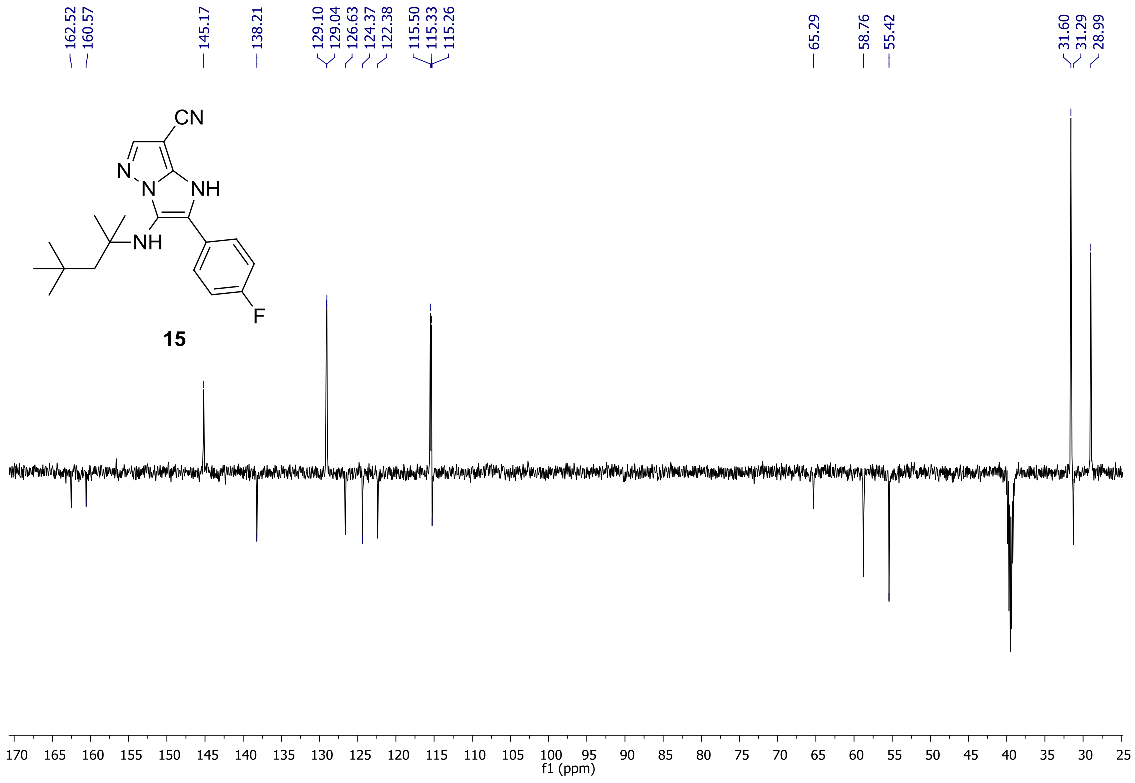

Methyl 2-((7-cyano-2-(4-fluorophenyl)-1*H*-imidazo[1,2-*b*]pyrazole-3-yl)amino)acetate (16)

^{1}H NMR (DMSO-d_6, 500 MHz)

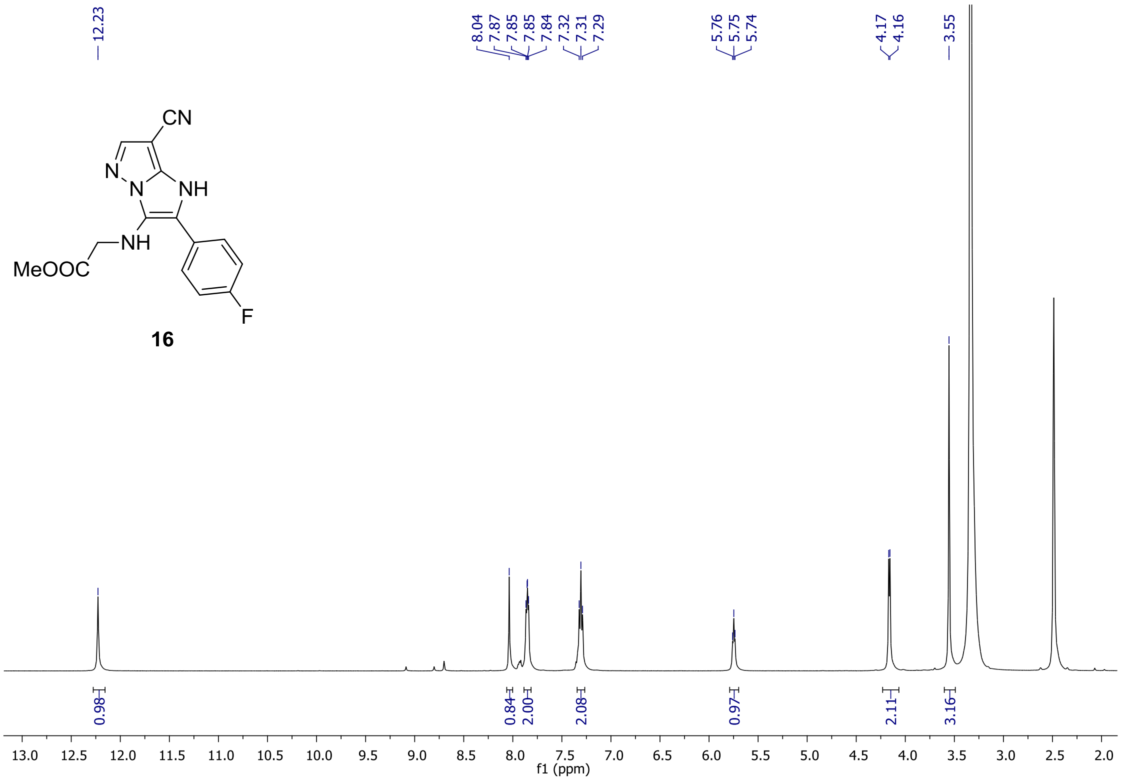

^{13}C NMR (DMSO-d_6, 125 MHz)

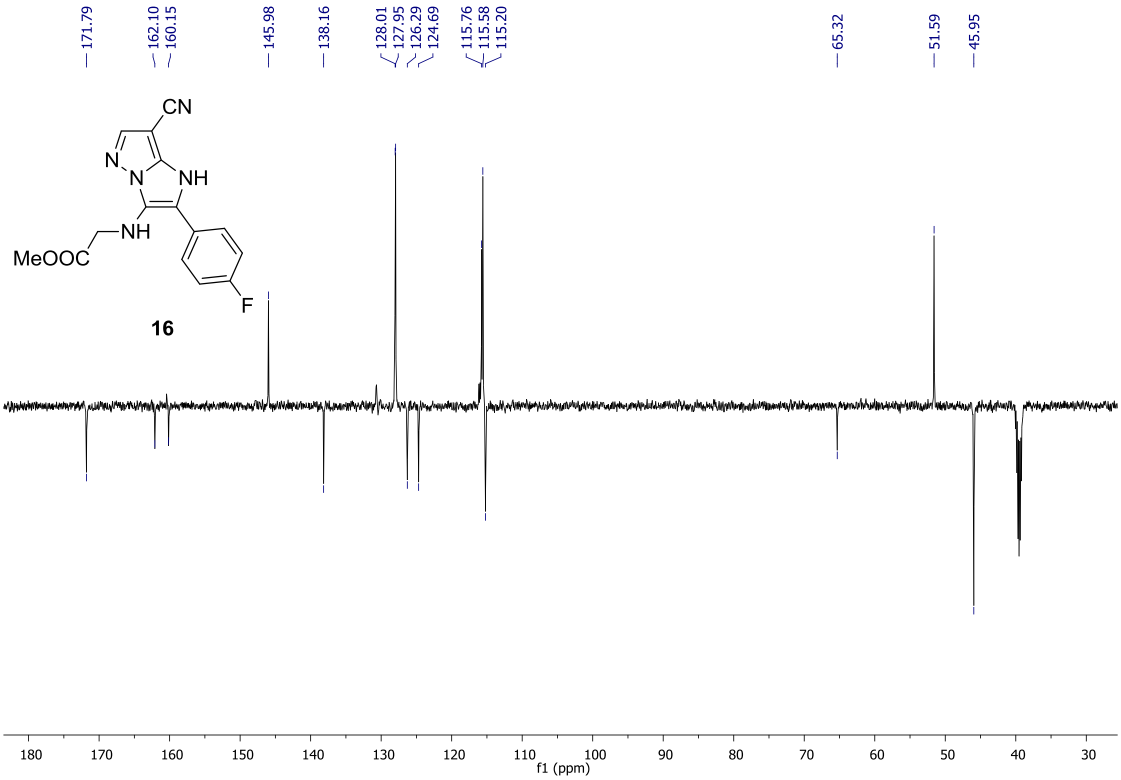

3-(Cyclohexylamino)-2-(4-fluorophenyl)-1*H*-imidazo[1,2-*b*]pyrazole-7-carbonitrile (17)

^{1}H NMR (DMSO-d_6, 500 MHz)

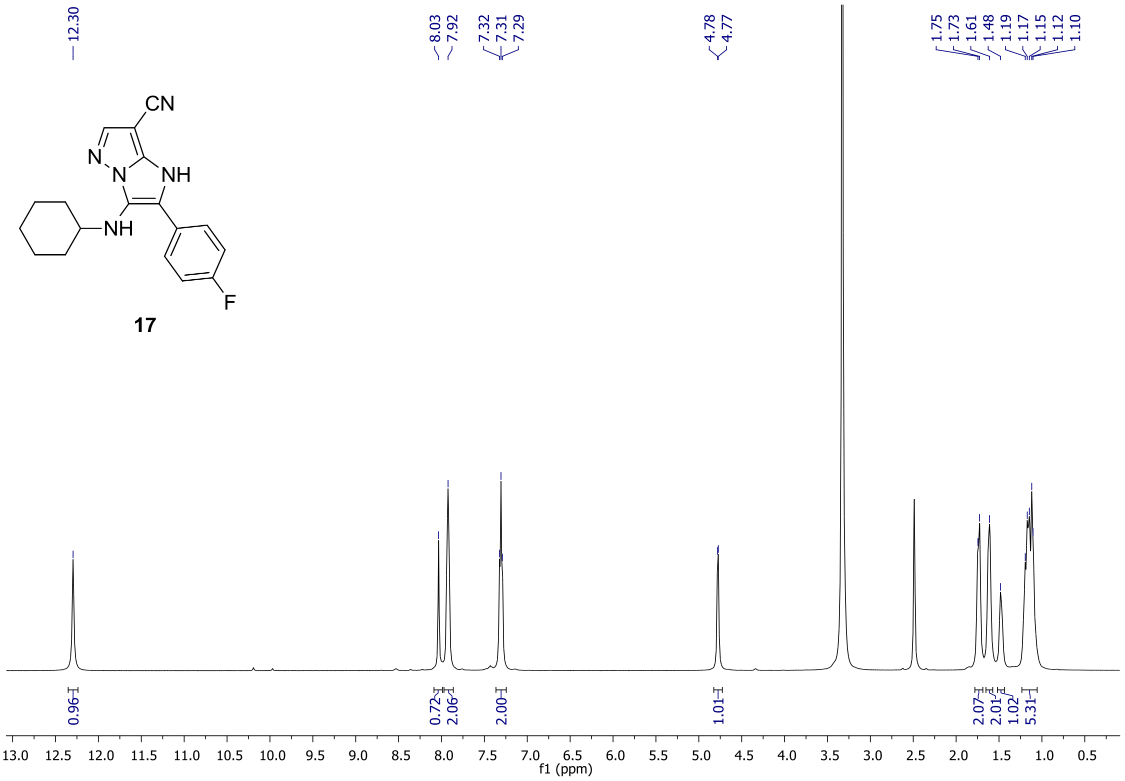

^{13}C NMR (DMSO-d_6, 125 MHz)

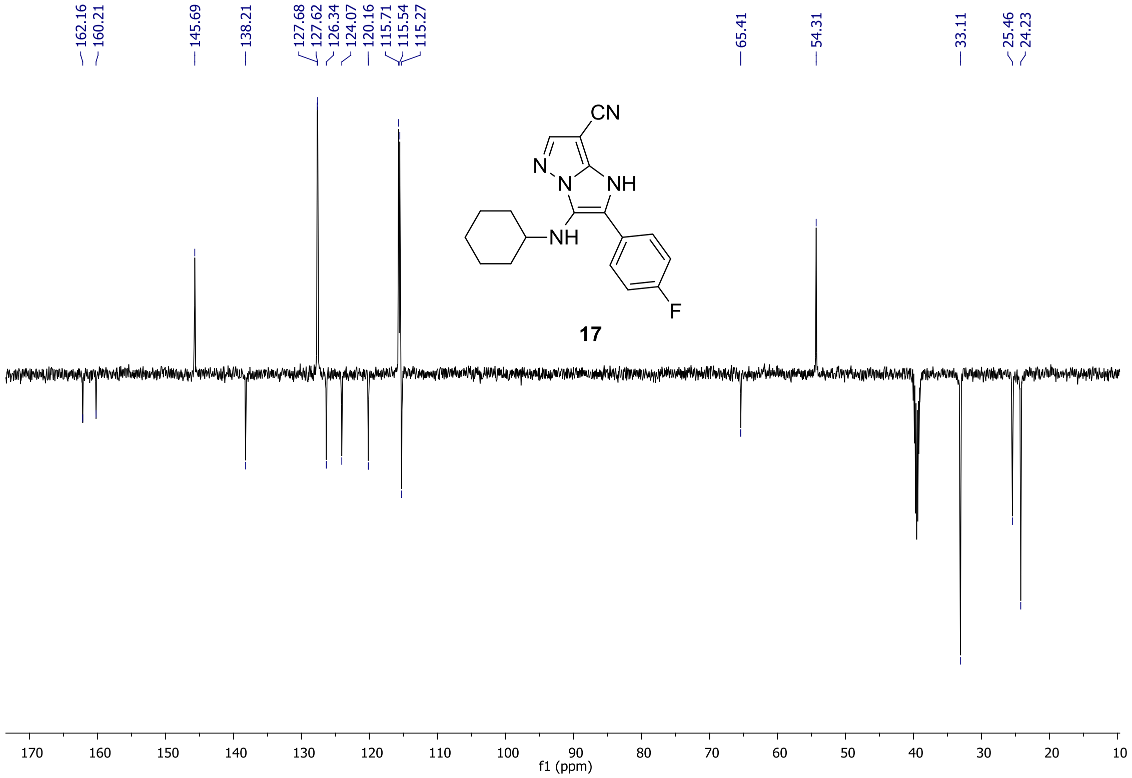

3-(*tert*-Butylamino)-2-(4-(trifluoromethyl)phenyl)-1*H*-imidazo[1,2-*b*]pyrazole-7-carbonitrile (18)

^{1}H NMR (DMSO-d_6, 500 MHz)

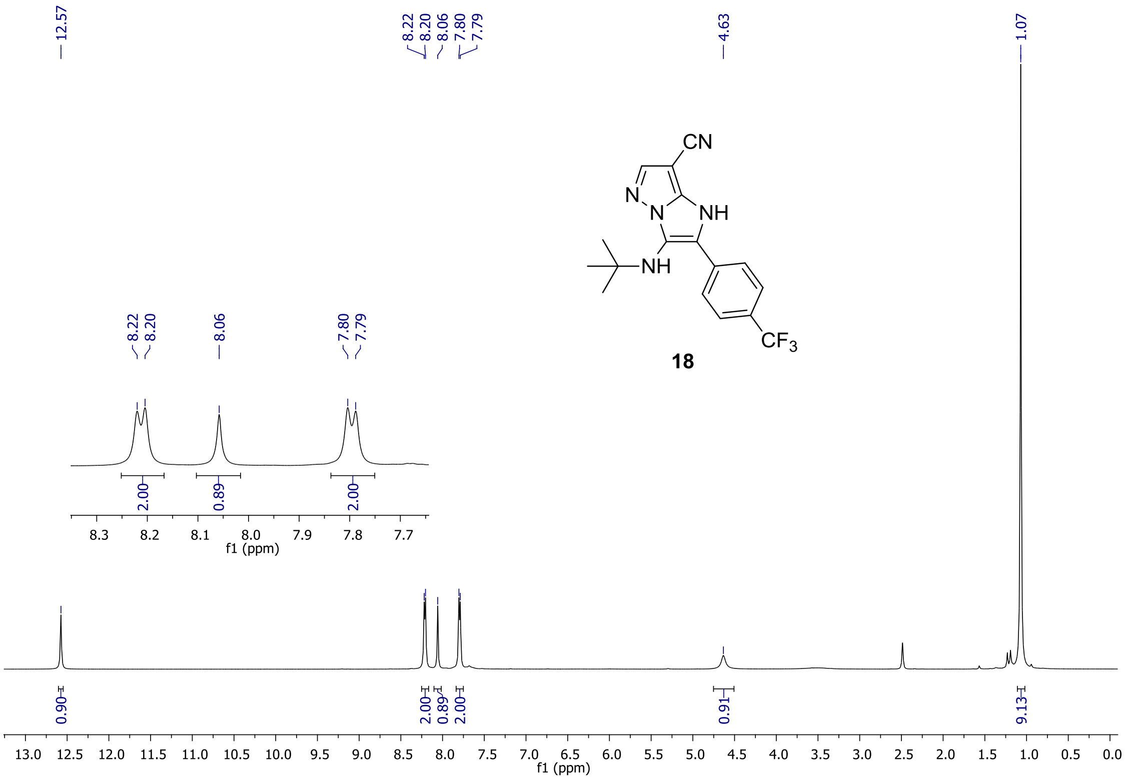

^{13}C NMR (DMSO-d_6, 125 MHz)

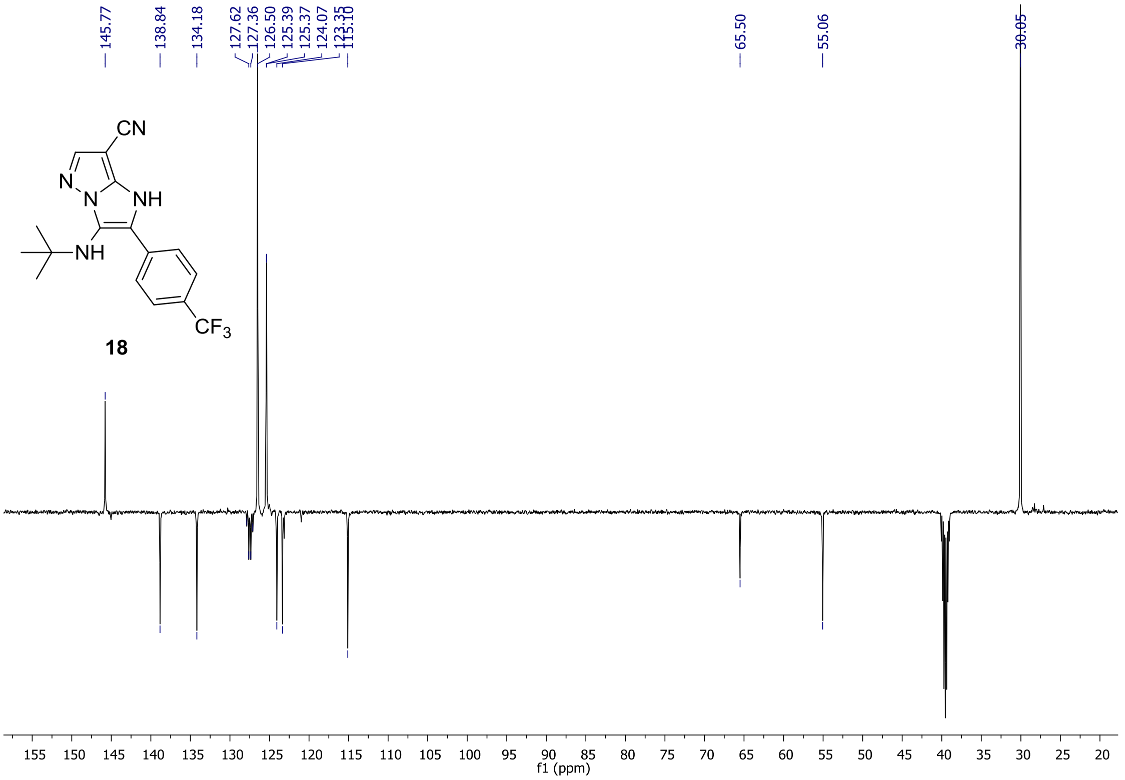

2-(4-(Trifluoromethyl)phenyl)-3-((2,4,4-trimethylpentan-2-yl)amino)-1*H*-imidazo[1,2-*b*]pyrazole-7-carbonitrile (19)

^{1}H NMR (DMSO-d_6, 500 MHz)

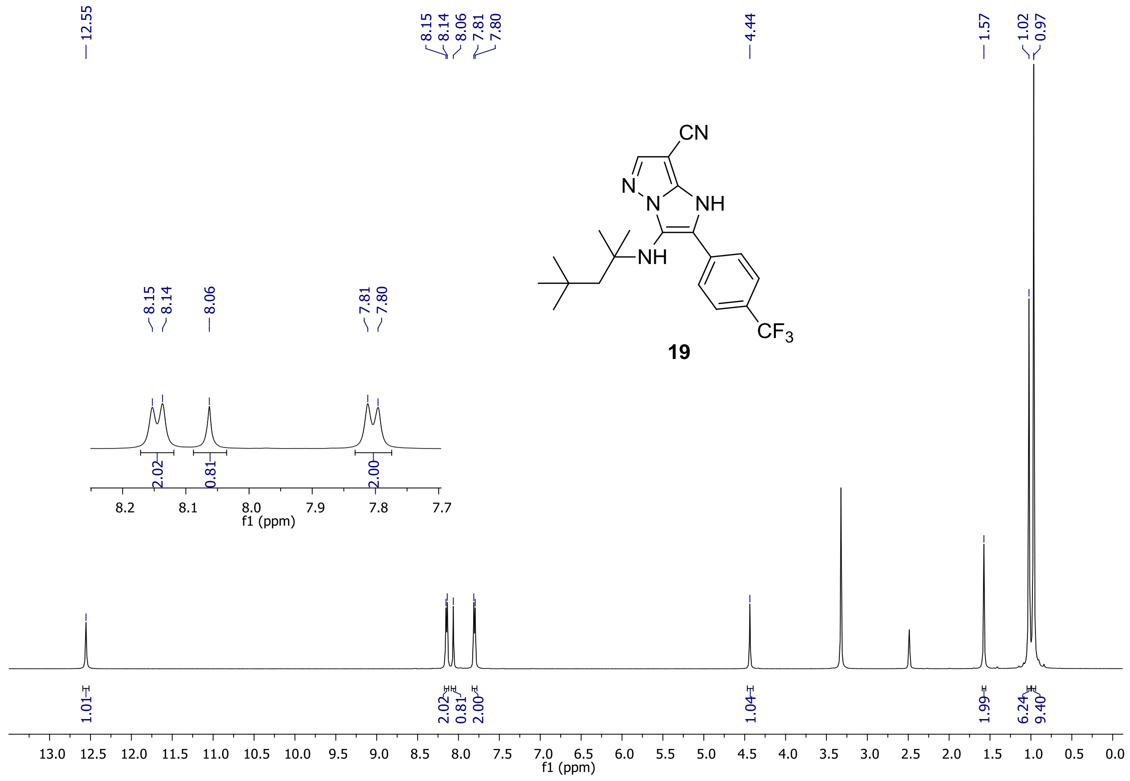

^{13}C NMR (DMSO-d_6, 125 MHz)

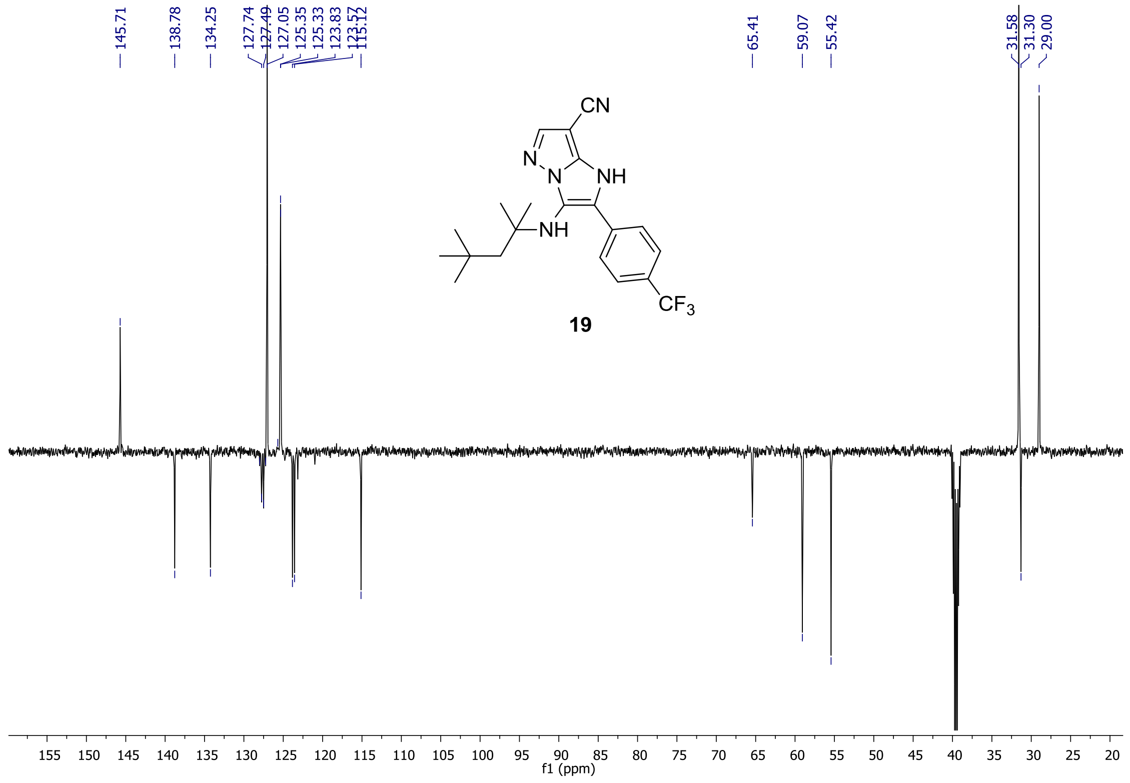

Methyl-2-((7-cyano-2-(4-(trifluoromethyl)phenyl)-1*H*-imidazo[1,2-*b*]pyrazole-3-yl)amino)acetate (20)

^{1}H NMR (DMSO-d_6, 500 MHz)

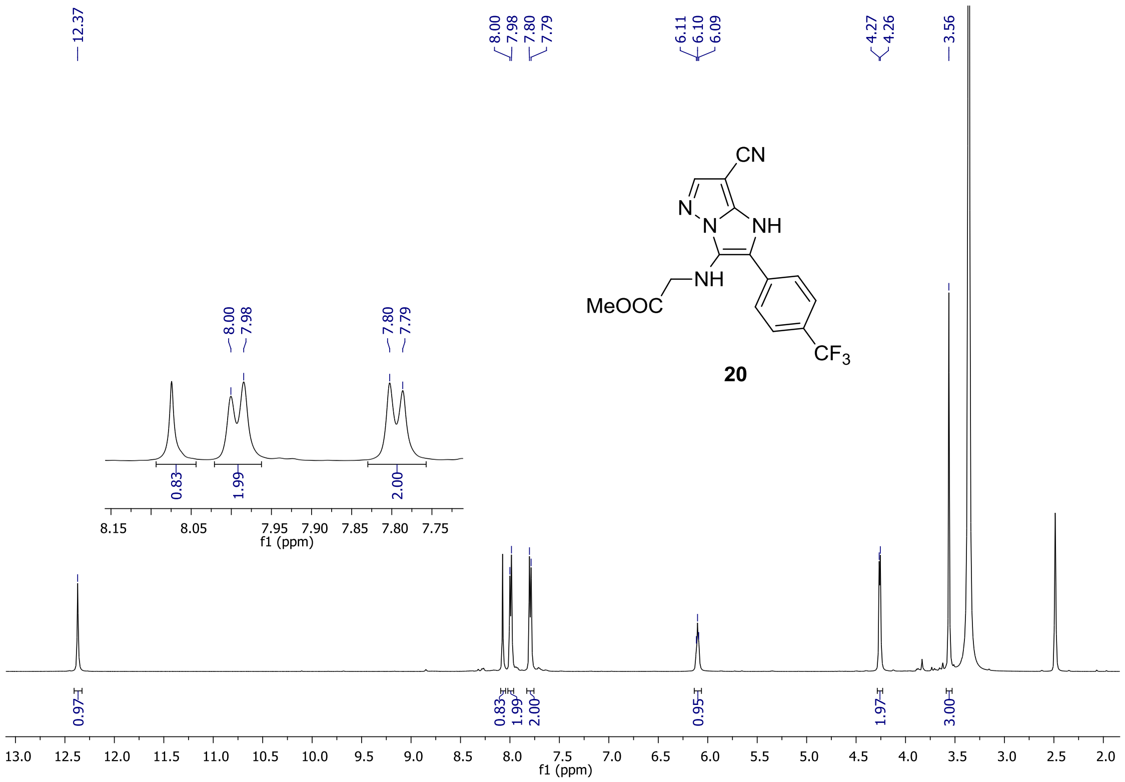

^{13}C NMR (DMSO-d_6, 125 MHz)

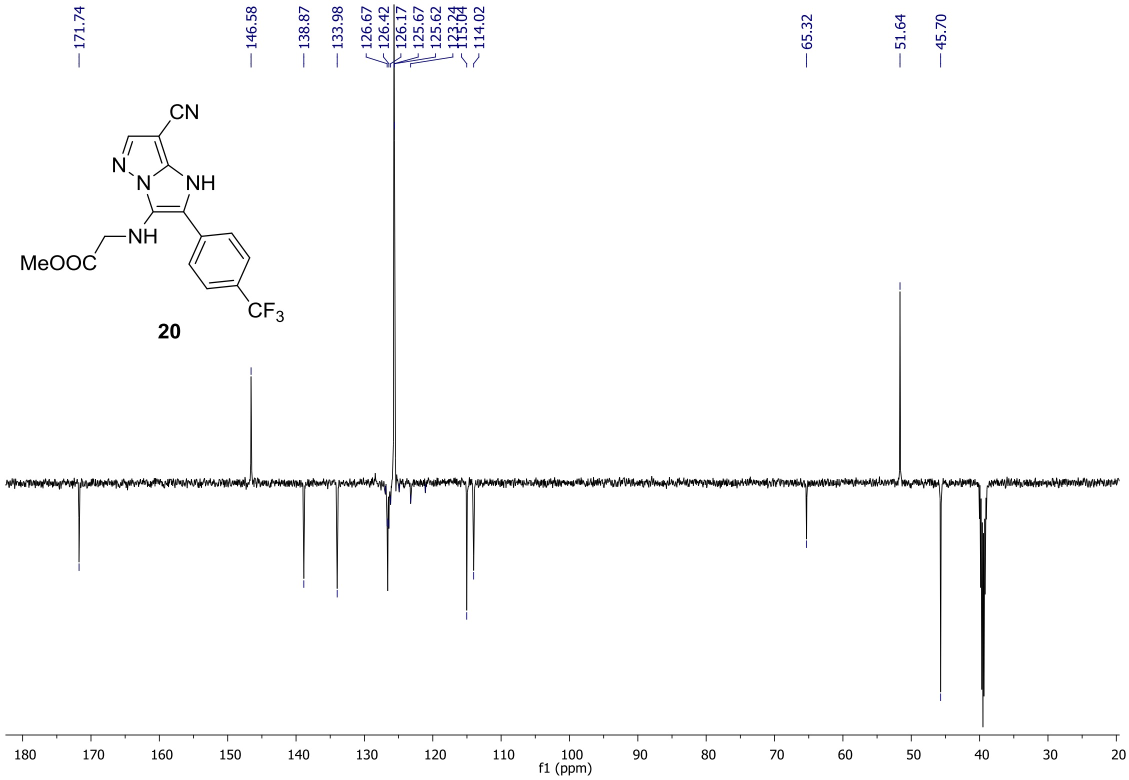

3-(Cyclohexylamino)-2-(4-(trifluoromethyl)phenyl)-1*H*-imidazo[1,2-*b*]pyrazole-7-carbonitrile (21)

^{1}H NMR (DMSO-d_6, 500 MHz)

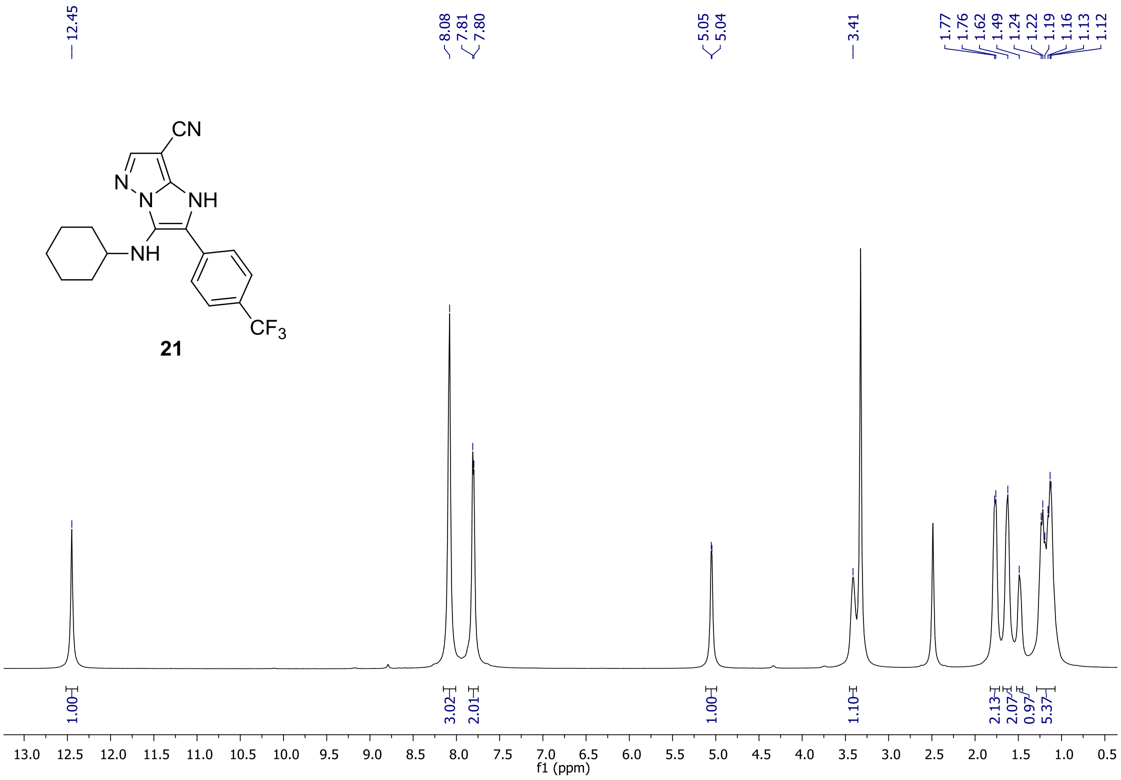

^{13}C NMR (DMSO-d_6, 125 MHz)

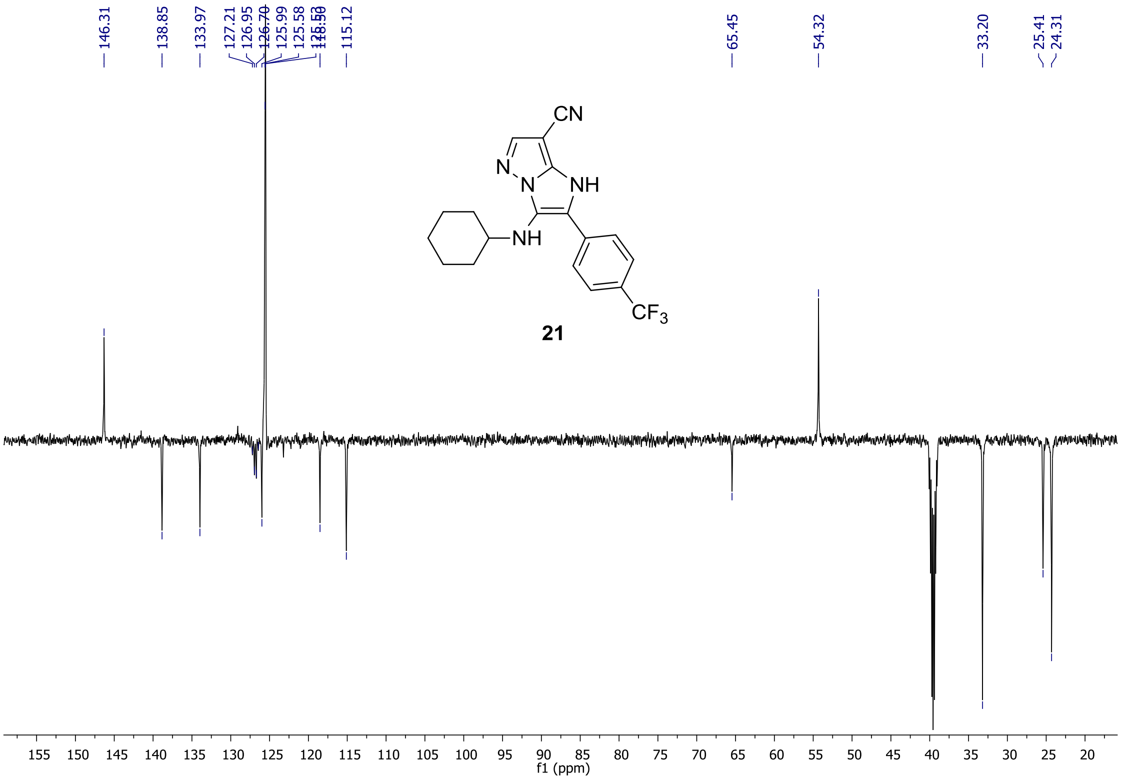

3-(*tert*-Butylamino)-2-(2-fluoro-4-(trifluoromethyl)phenyl)-1*H*-imidazo[1,2-*b*]pyrazole-7-carbonitrile (22)

^{1}H NMR (DMSO-d_6, 500 MHz)

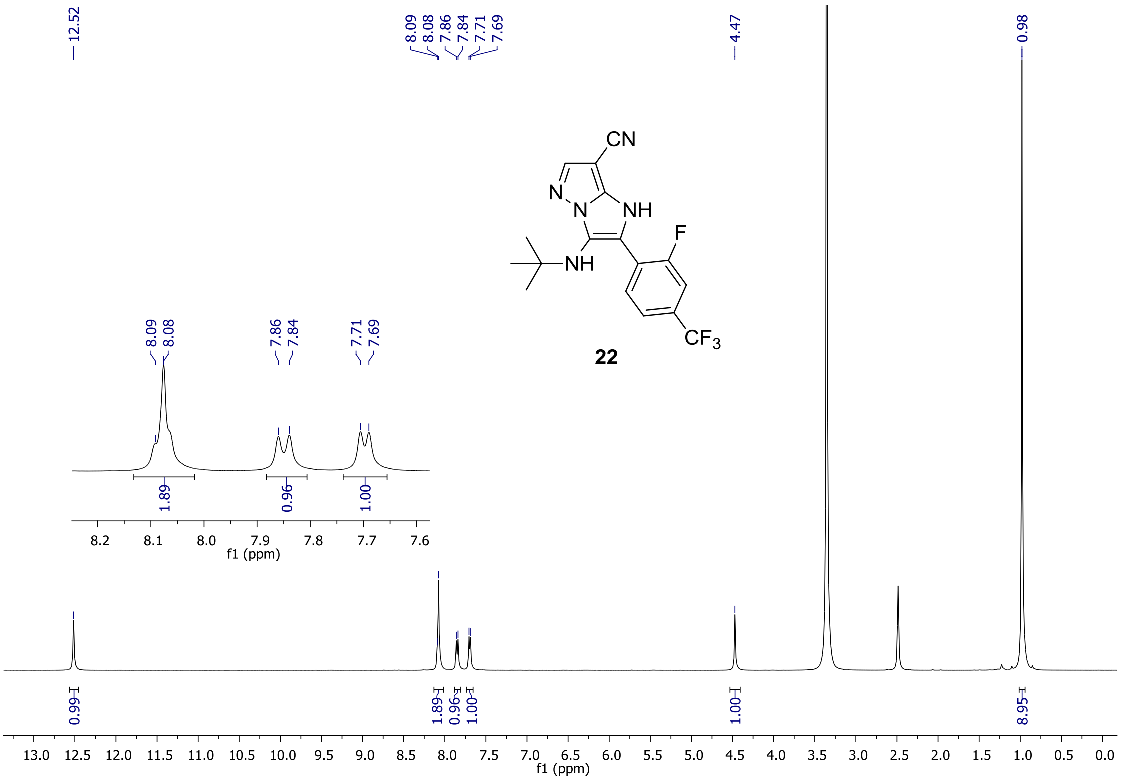

^{13}C NMR (DMSO-d_6, 125 MHz)

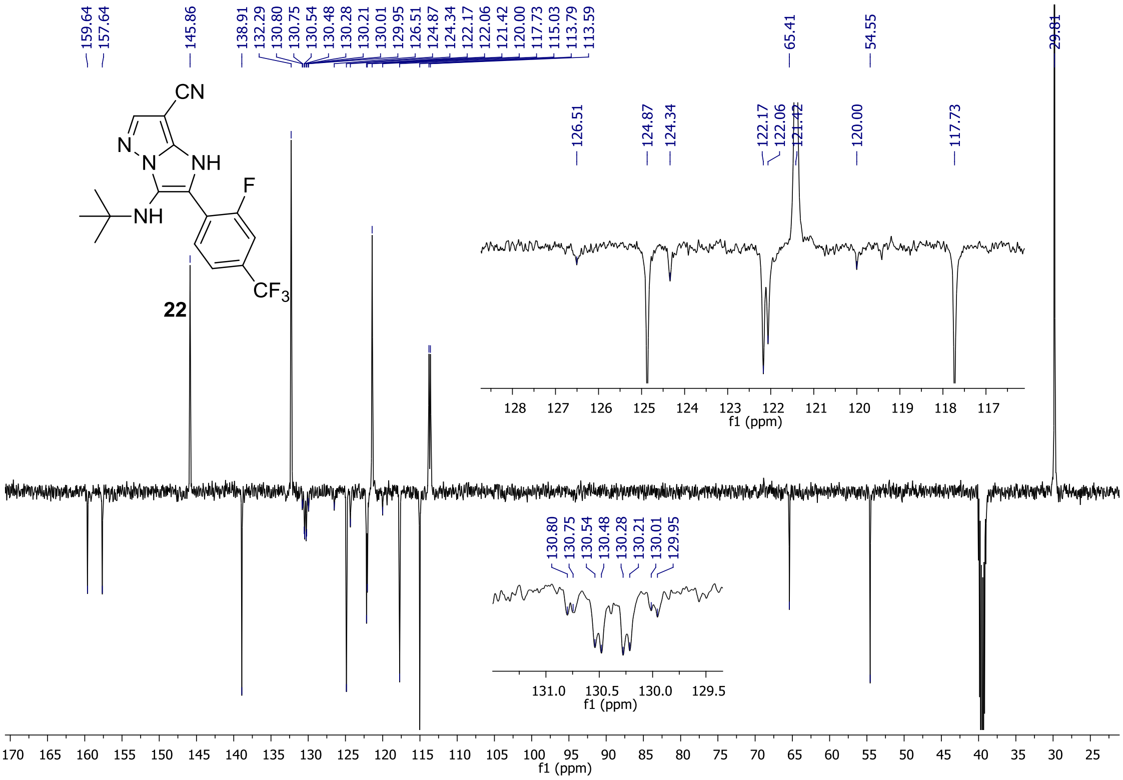

2-(2-Fluoro-4-(trifluoromethyl)phenyl)-3-((2,4,4-trimethylpentan-2-yl)amino)-1*H*-imidazo[1,2-*b*]pyrazole-7-carbonitrile (23)

^{1}H NMR (DMSO-d_6, 500 MHz)

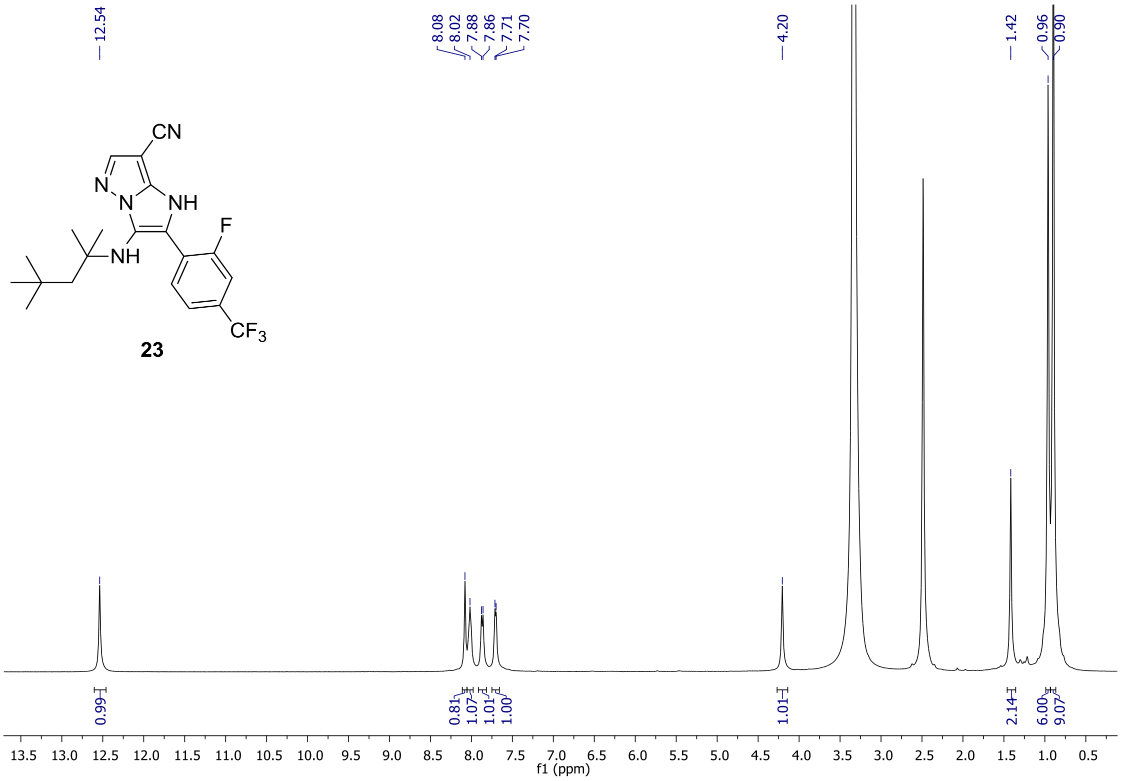

^{13}C NMR (DMSO-d_6, 125 MHz)

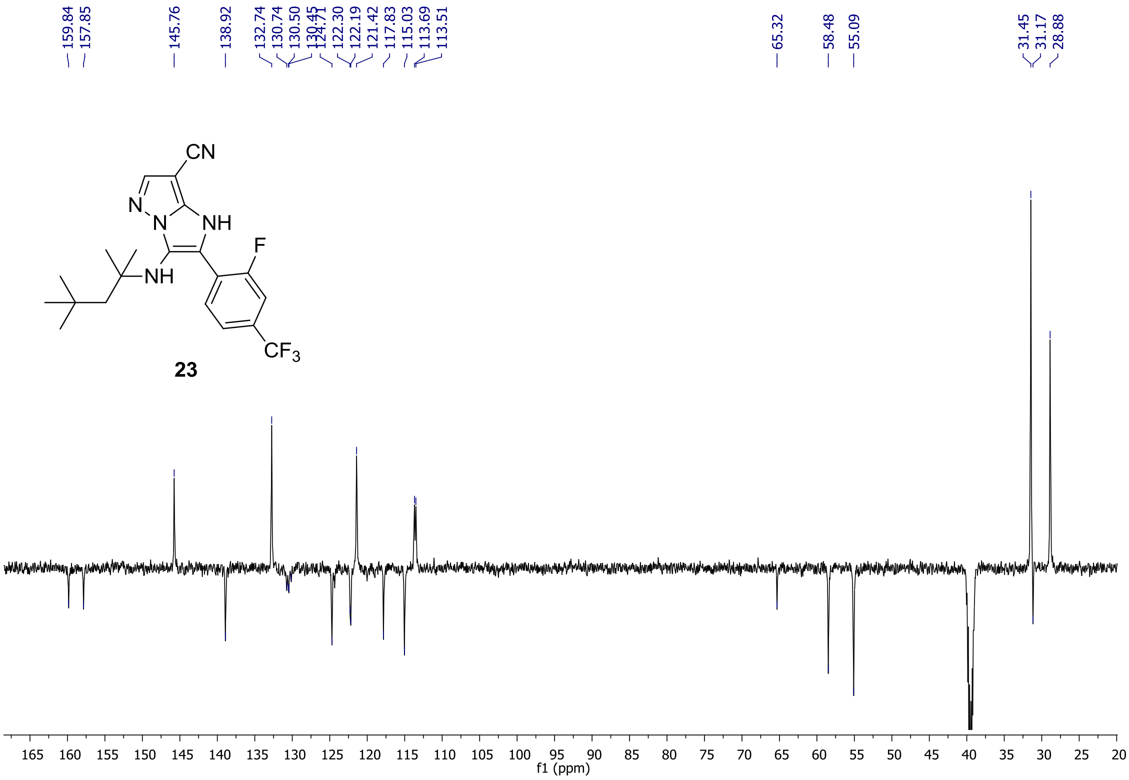

Methyl 2-((7-cyano-2-(2-fluoro-4-(trifluoromethyl)phenyl)-1*H*-imidazo[1,2-*b*]pyrazol-3-yl)amino)acetate (24)

^{1}H NMR (DMSO-d_6, 500 MHz)

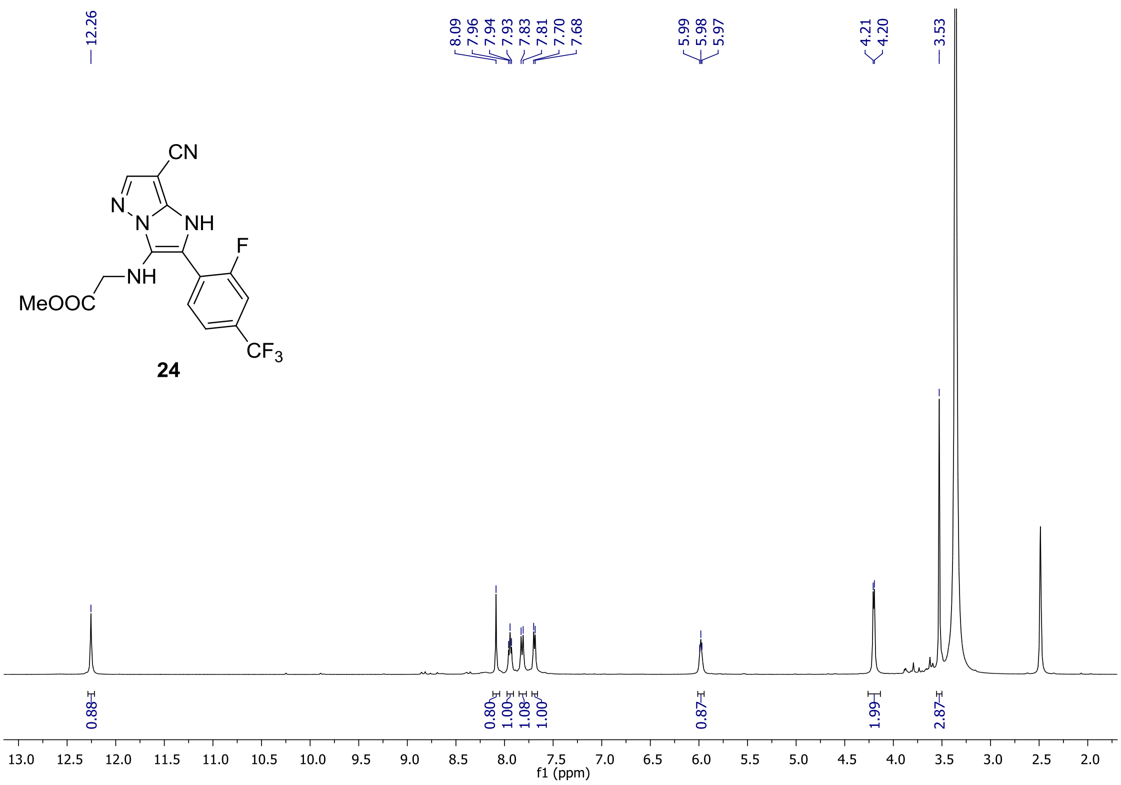

^{13}C NMR (DMSO-d_6, 125 MHz)

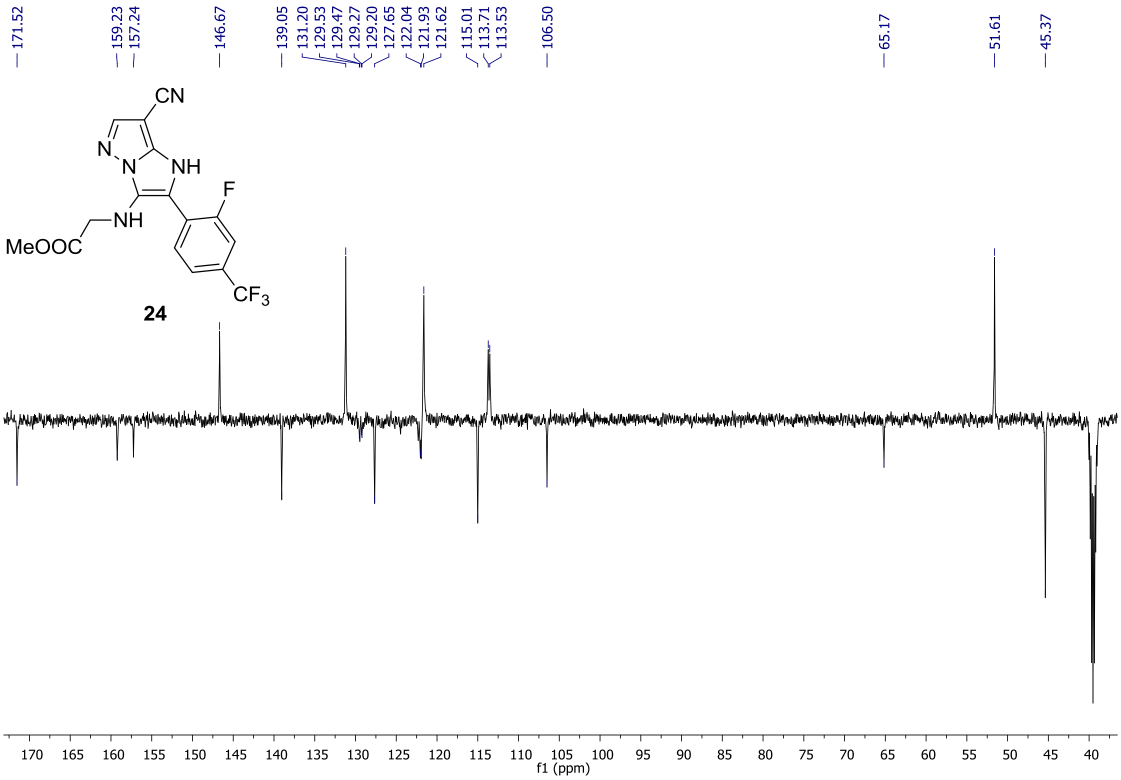

3-(Cyclohexylamino)-2-(2-fluoro-4-(trifluoromethyl)phenyl)-1*H*-imidazo[1,2-*b*]pyrazole-7-carbonitrile (25)

^{1}H NMR (DMSO-d_6, 500 MHz)

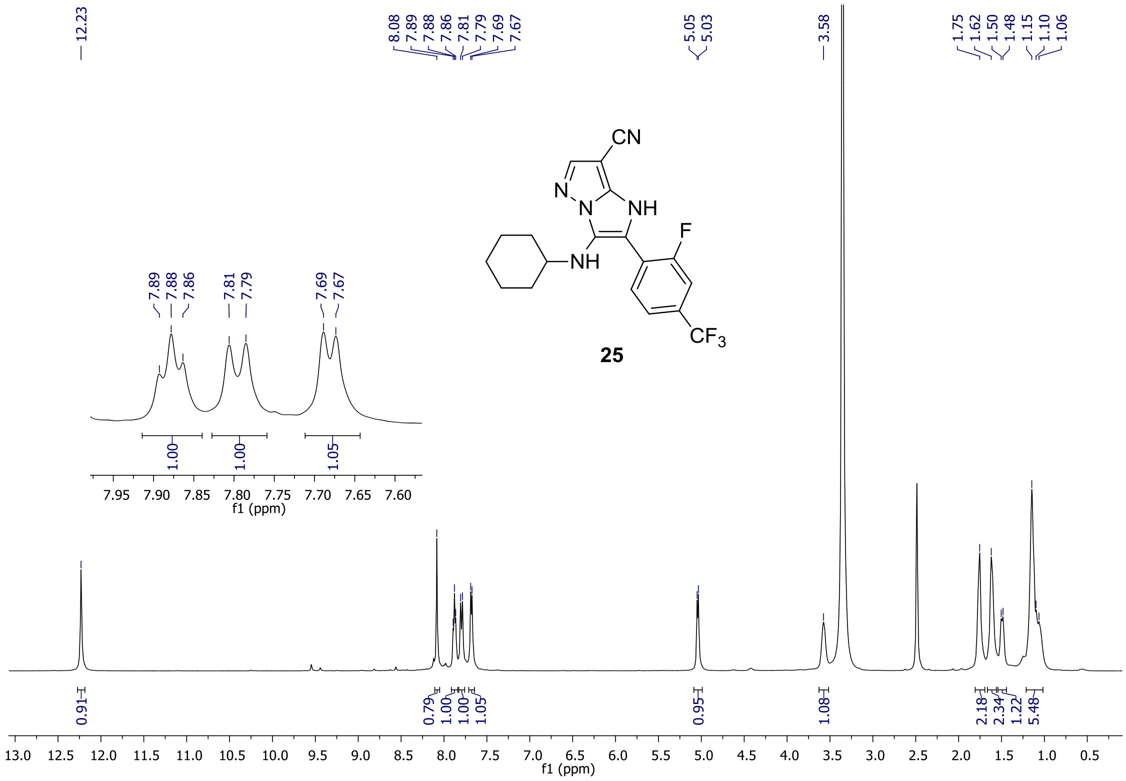

^{13}C NMR (DMSO-d_6, 125 MHz)

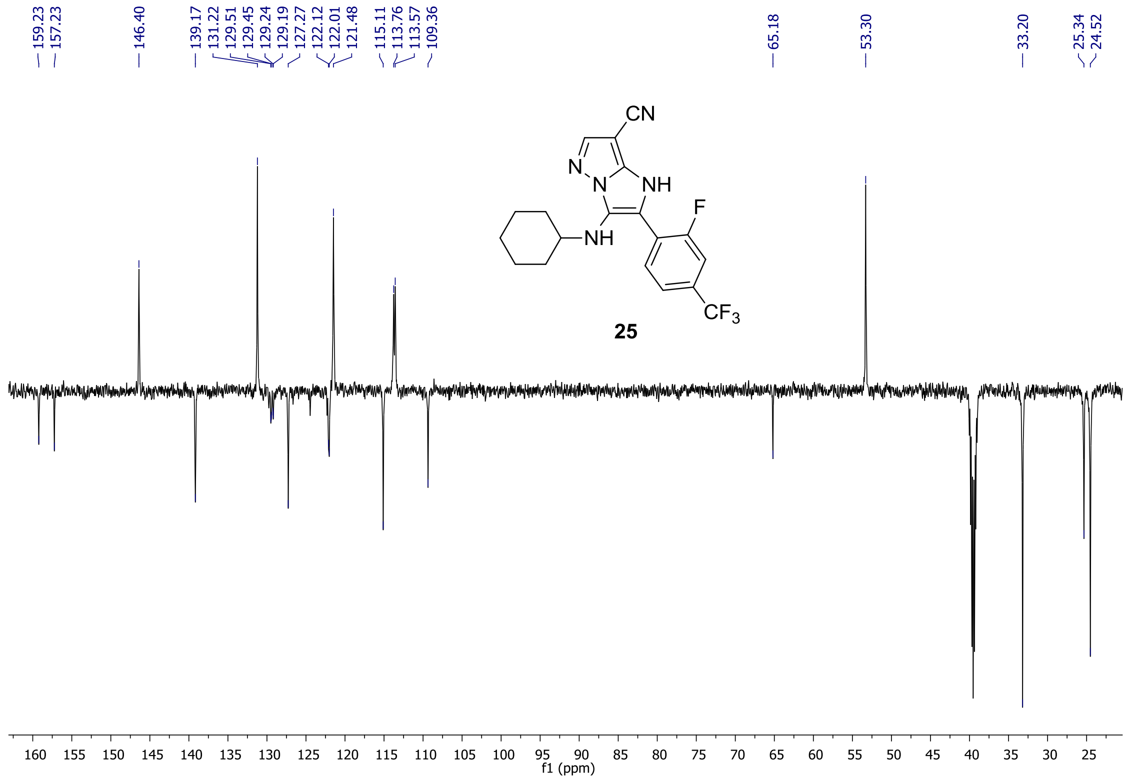

3-(*tert*-Butylamino)-2-(3,4-difluorophenyl)-1*H*-imidazo[1,2-*b*]pyrazole-7-carbonitrile (26)

^{1}H NMR (DMSO-d_6, 500 MHz)

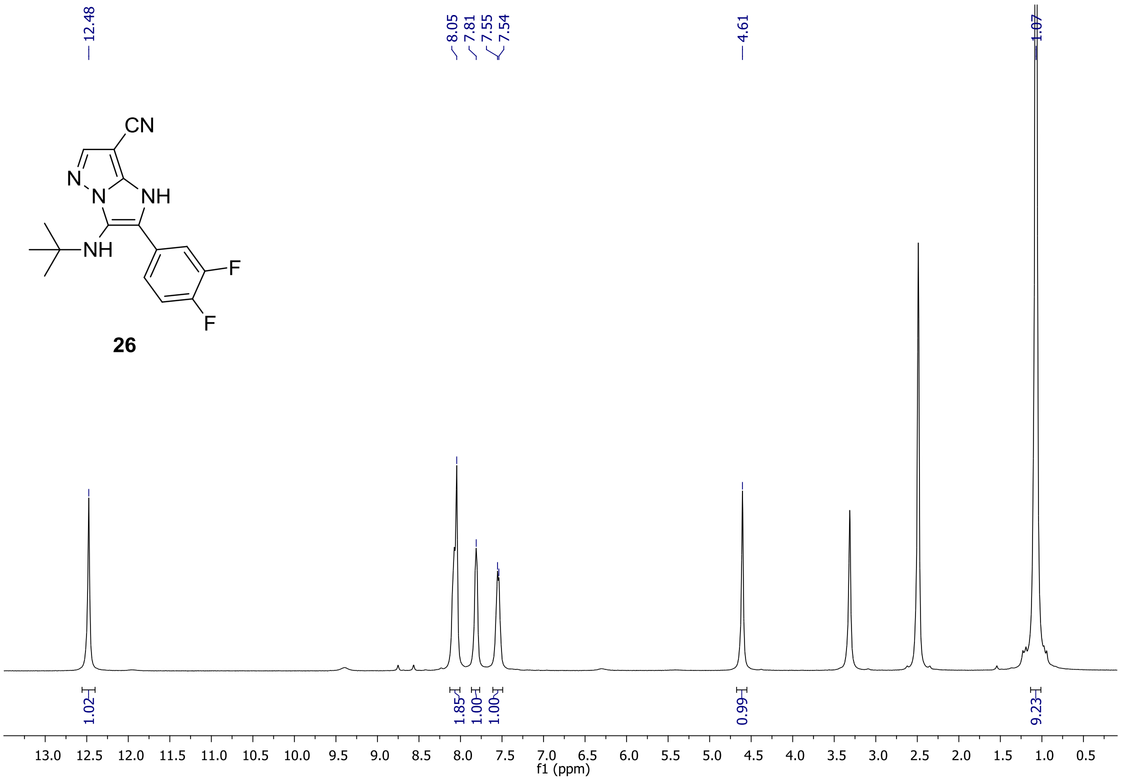

^{13}C NMR (DMSO-d_6, 125 MHz)

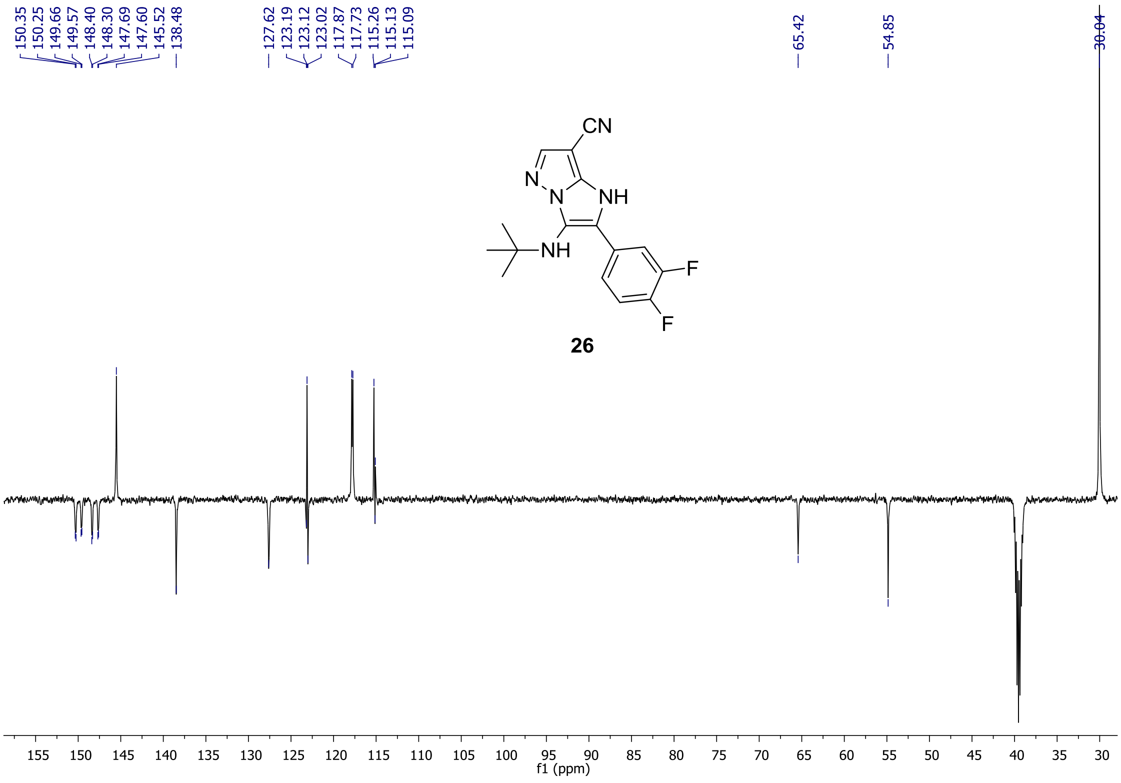

2-(3,4-Difluorophenyl)-3-((2,4,4-trimethylpentan-2-yl)amino)-1*H*-imidazo[1,2-*b*]pyrazole-7-carbonitrile (27)

^{1}H NMR (DMSO-d_6, 500 MHz)

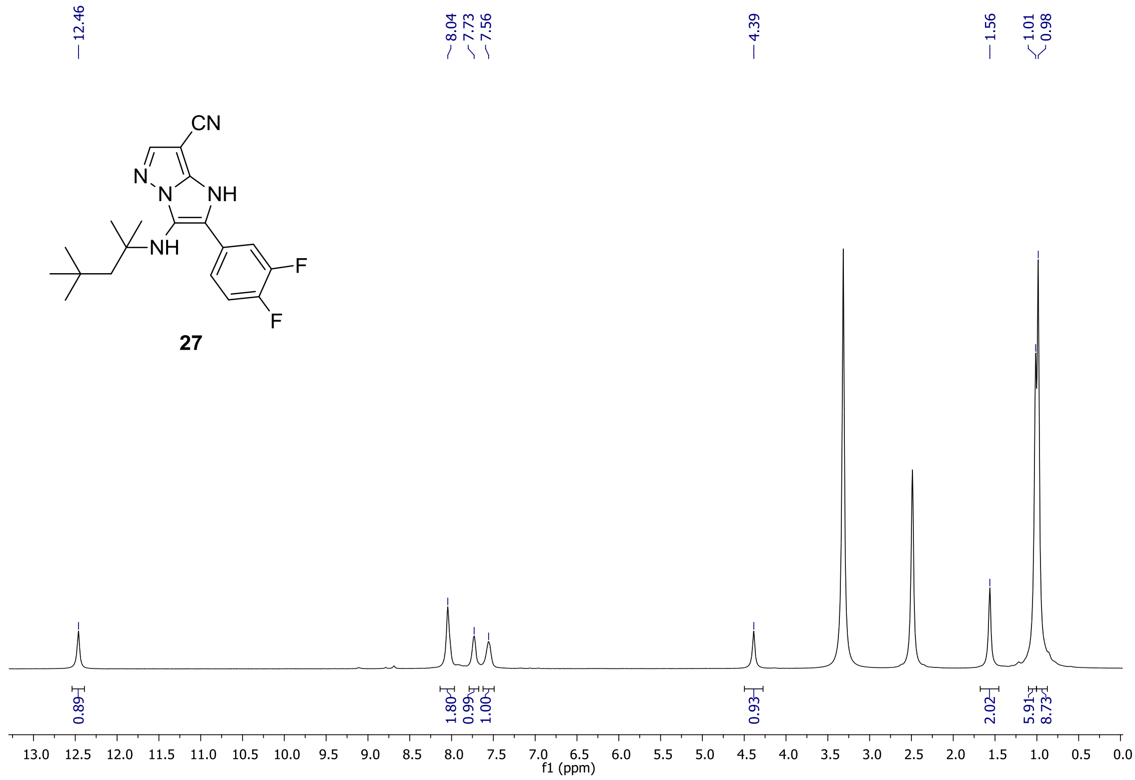

^{13}C NMR (DMSO-d_6, 125 MHz)

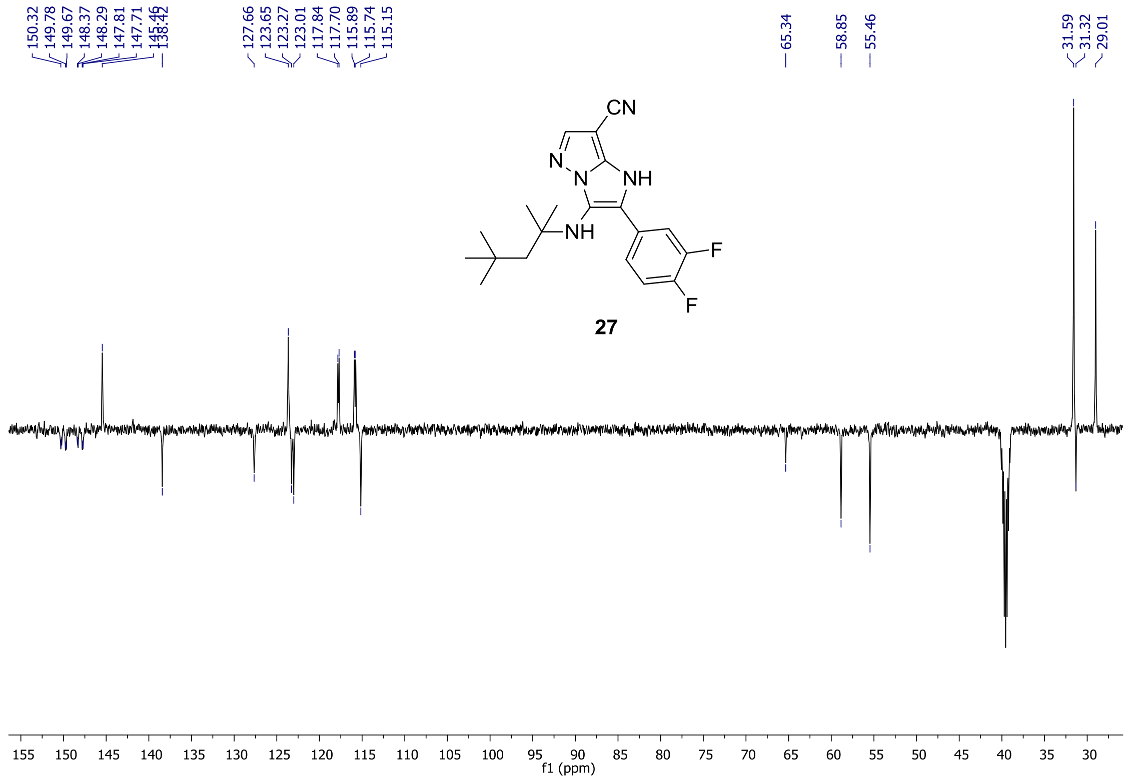

Methyl 2-((7-cyano-2-(3,4-difluorophenyl)-1*H*-imidazo[1,2-*b*]pyrazol-3-yl)amino)acetate (28)

^{1}H NMR (DMSO-d_6, 500 MHz)

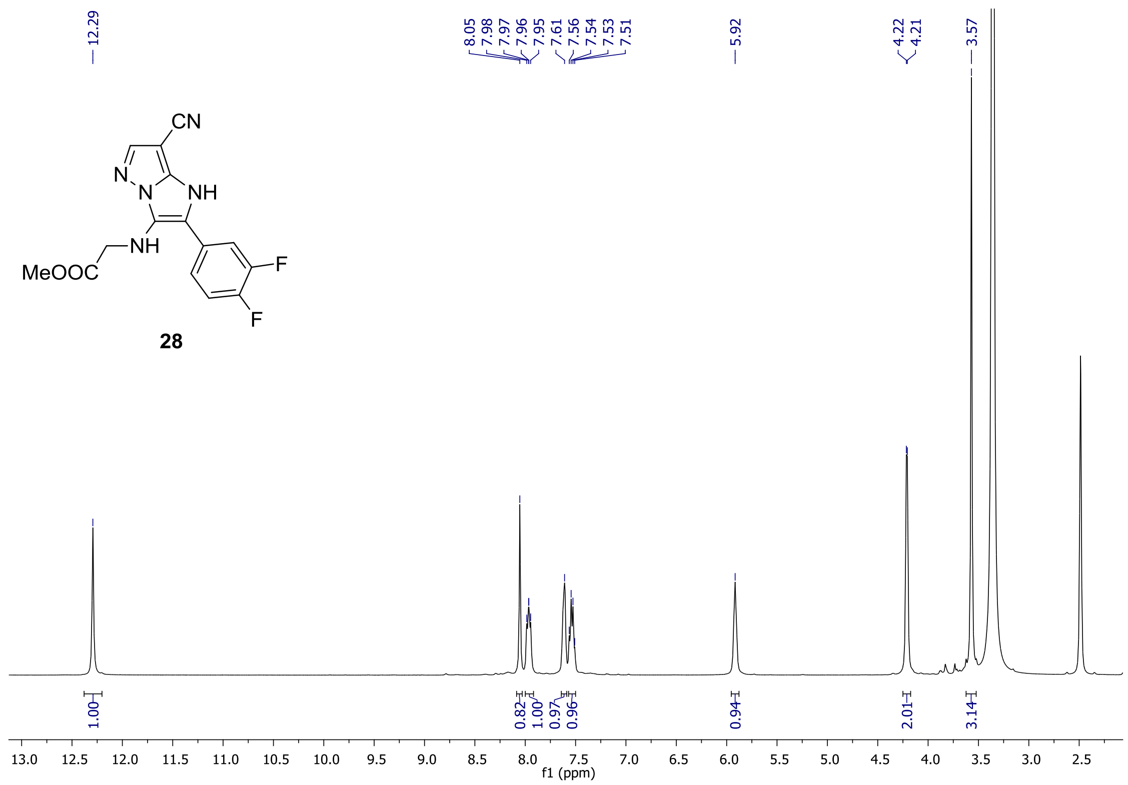

^{13}C NMR (DMSO-d_6, 125 MHz)

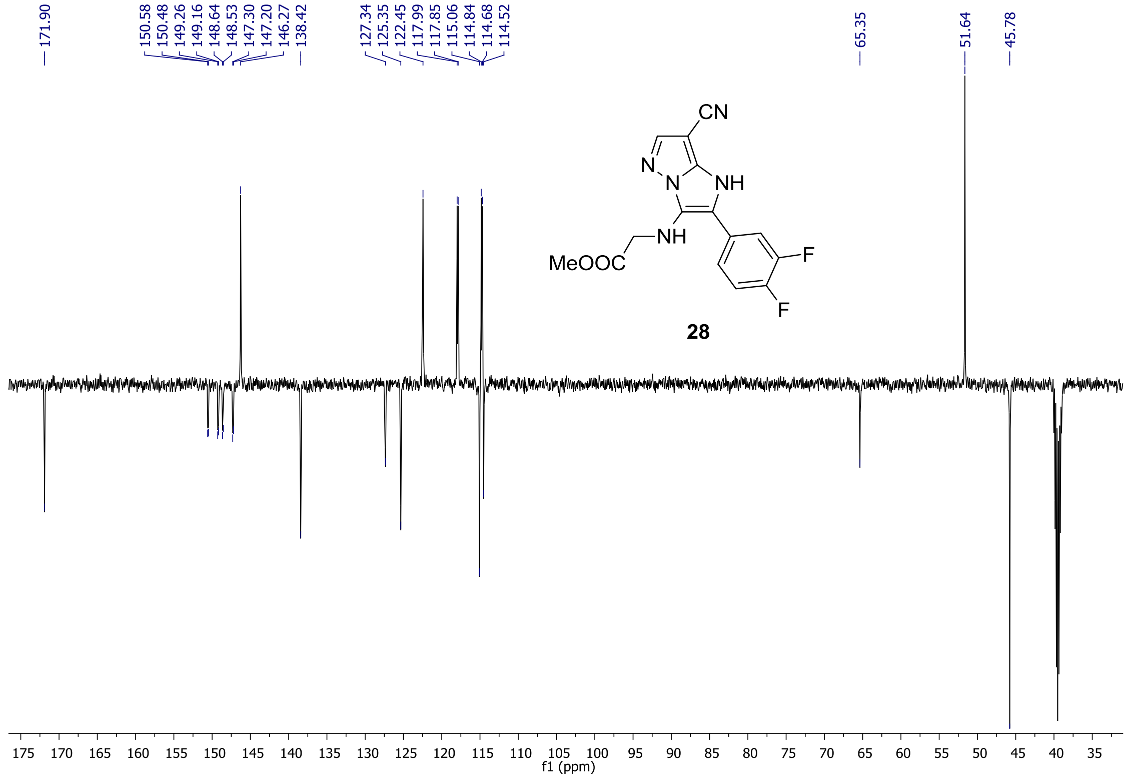

3-(Cyclohexylamino)-2-(3,4-difluorophenyl)-1*H*-imidazo[1,2-*b*]pyrazole-7-carbonitrile (29)

^{1}H NMR (DMSO-d_6, 500 MHz)

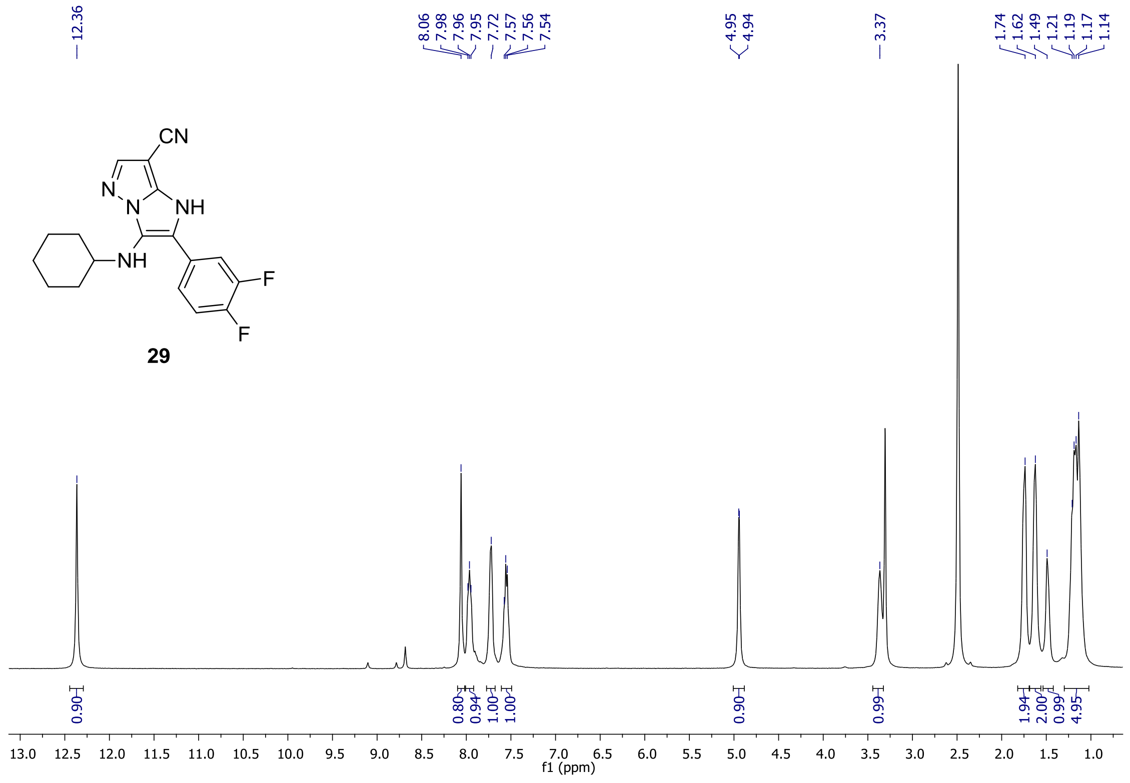

^{13}C NMR (DMSO-d_6, 125 MHz)

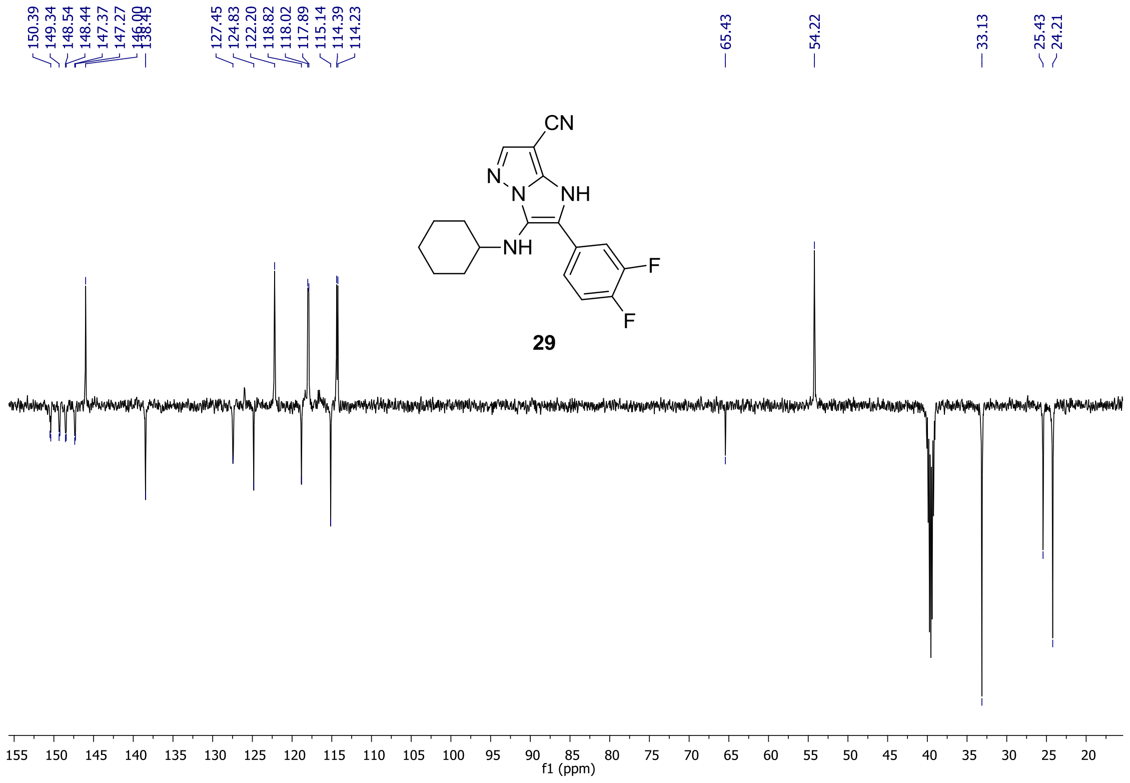

3-(*tert*-Butylamino)-2-(4-fluoro-3-(trifluoromethyl)phenyl)-1*H*-imidazo[1,2-*b*]pyrazole-7-carbonitrile (30)

^{1}H NMR (DMSO-d_6, 500 MHz)

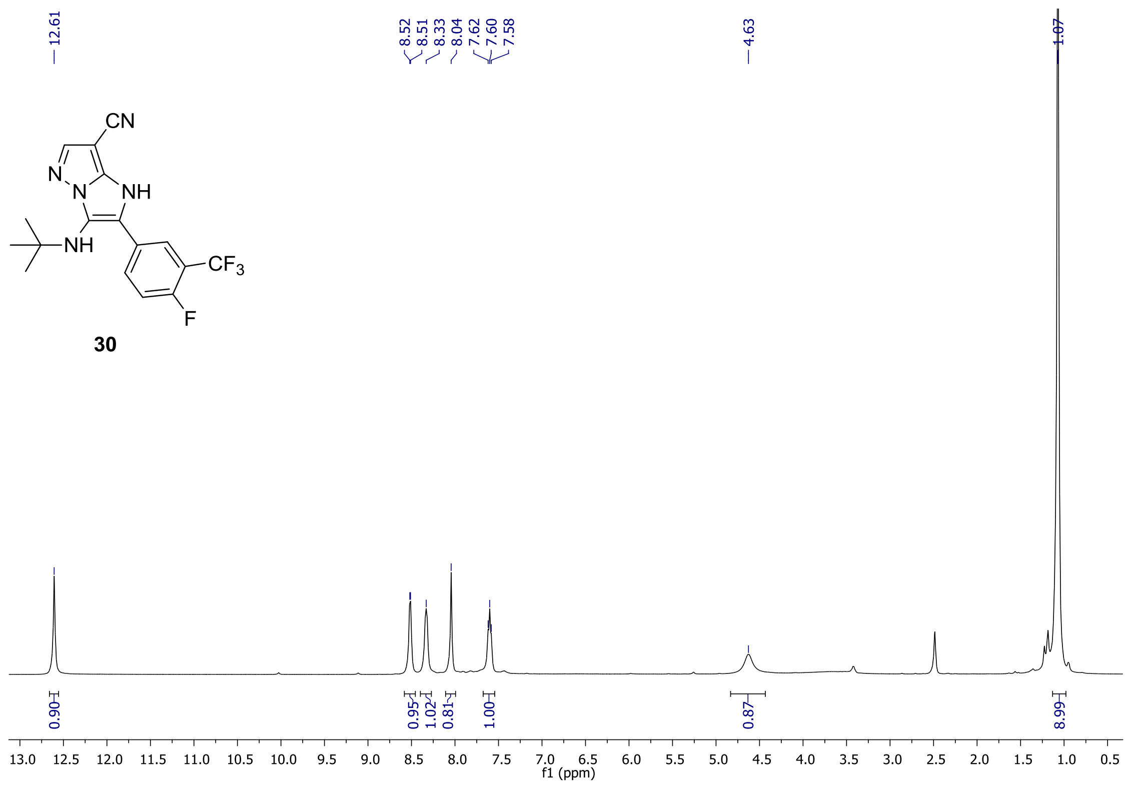

^{13}C NMR (DMSO-d_6, 125 MHz)

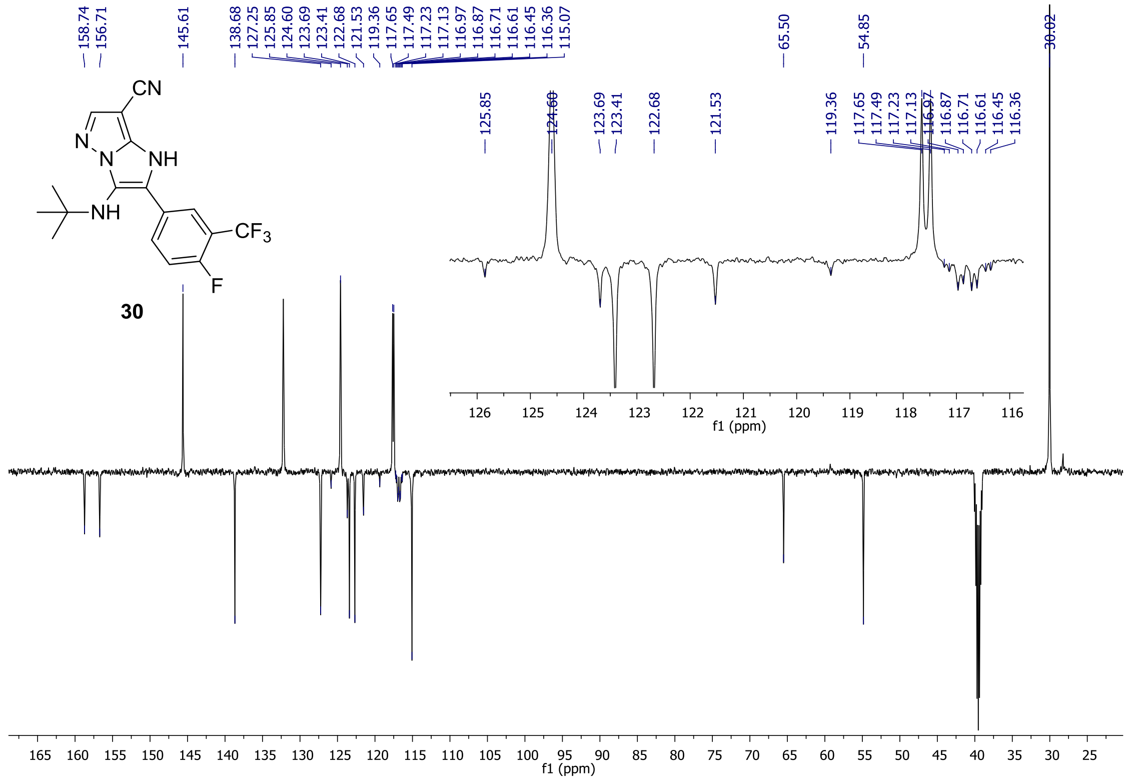

2-(4-Fluoro-3-(trifluoromethyl)phenyl)-3-((2,4,4-trimethylpentan-2-yl)amino)-1*H*-imidazo[1,2-*b*]pyrazole-7-carbonitrile (31)

^{1}H NMR (DMSO-d_6, 500 MHz)

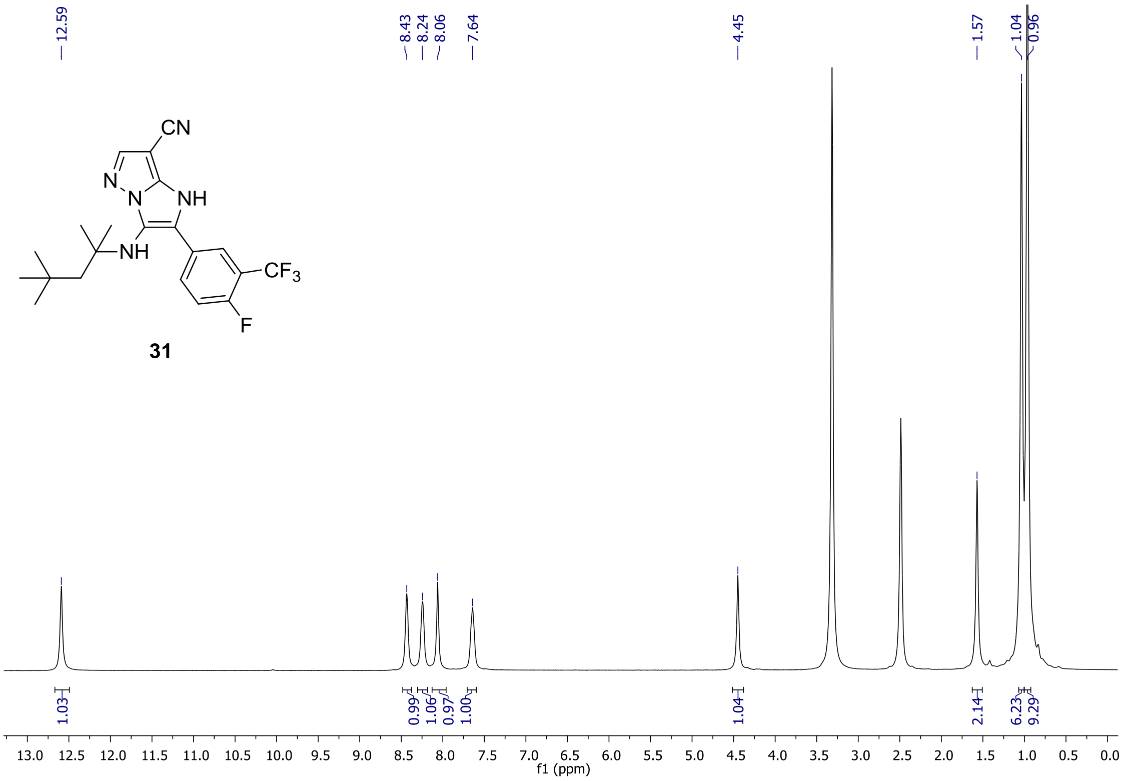

^{13}C NMR (DMSO-d_6, 125 MHz)

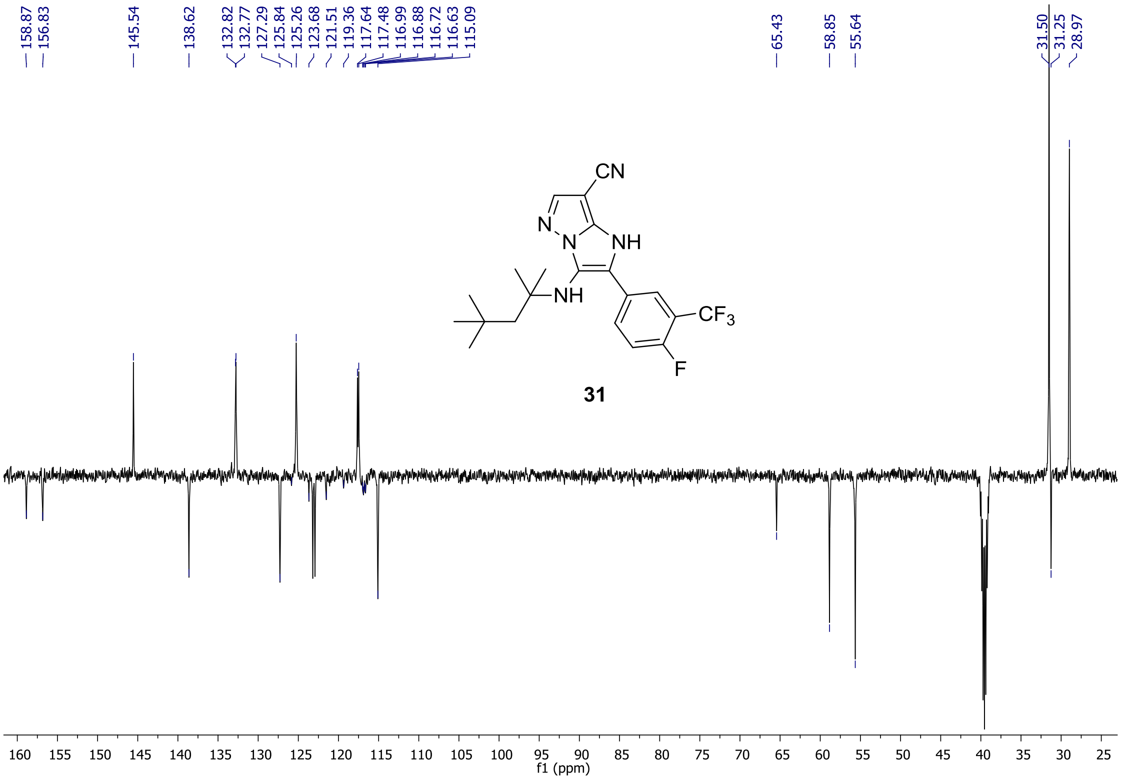

Methyl 2-((7-cyano-2-(4-fluoro-3-(trifluoromethyl)phenyl)-1*H*-imidazo[1,2-*b*]pyrazol-3-yl)amino)acetate (32)

^{1}H NMR (DMSO-d_6, 500 MHz)

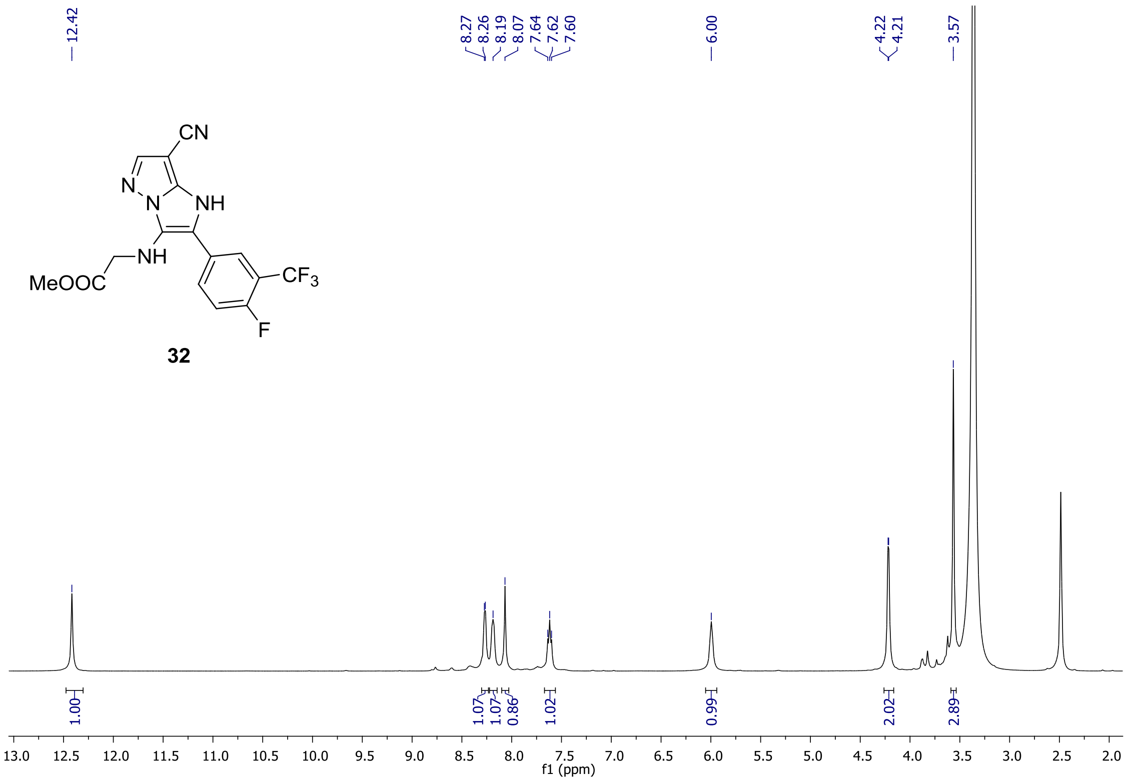

^{13}C NMR (DMSO-d_6, 125 MHz)

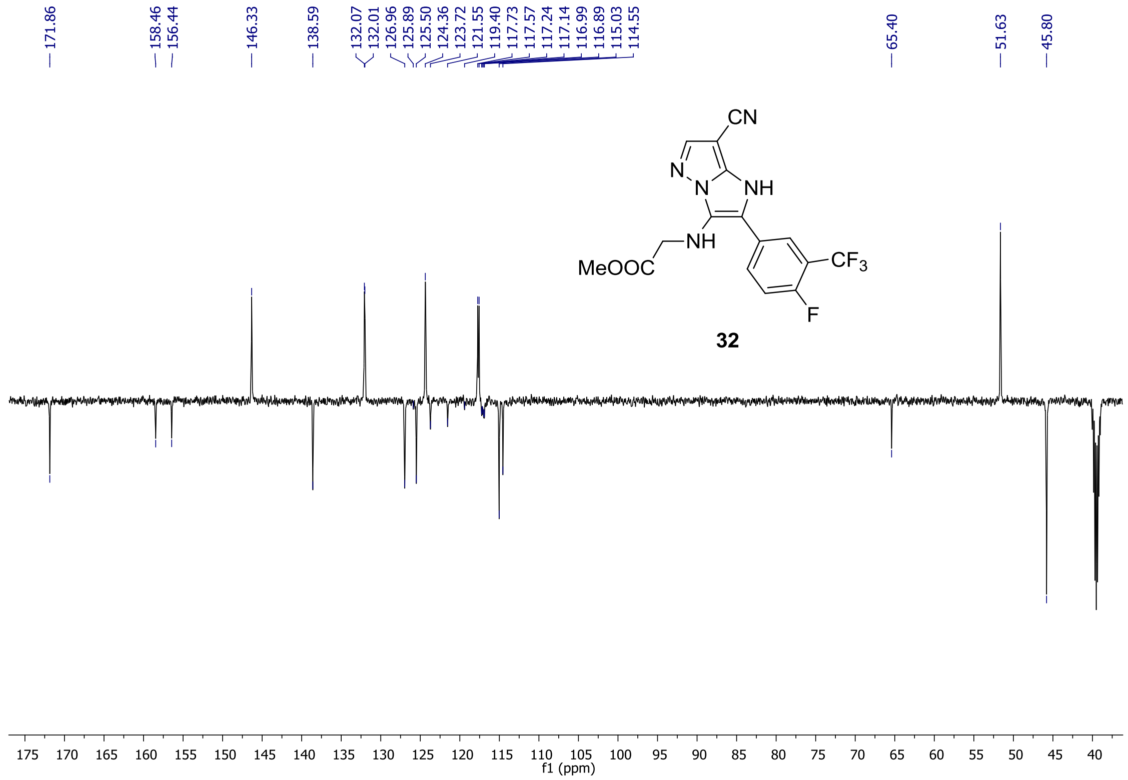

3-(Cyclohexylamino)-2-(4-fluoro-3-(trifluoromethyl)phenyl)-1*H*-imidazo[1,2-*b*]pyrazole-7-carbonitrile (33)

^{1}H NMR (DMSO-d_6, 500 MHz)

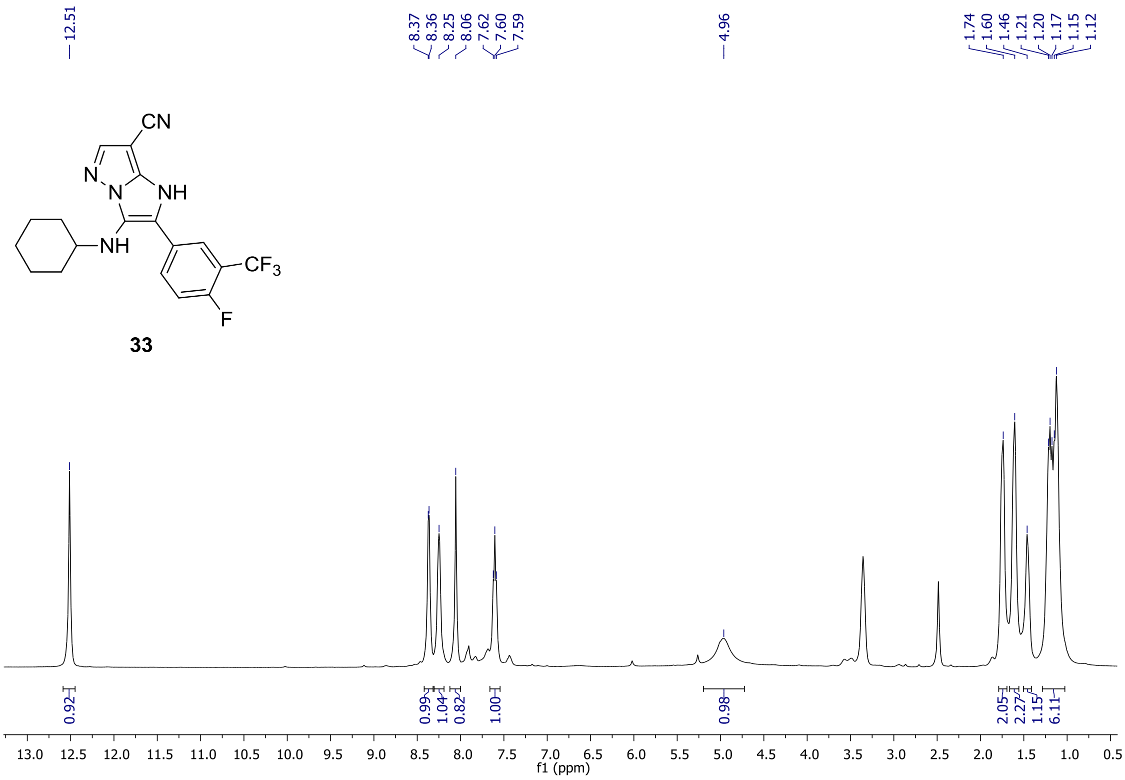

^{13}C NMR (DMSO-d_6, 125 MHz)

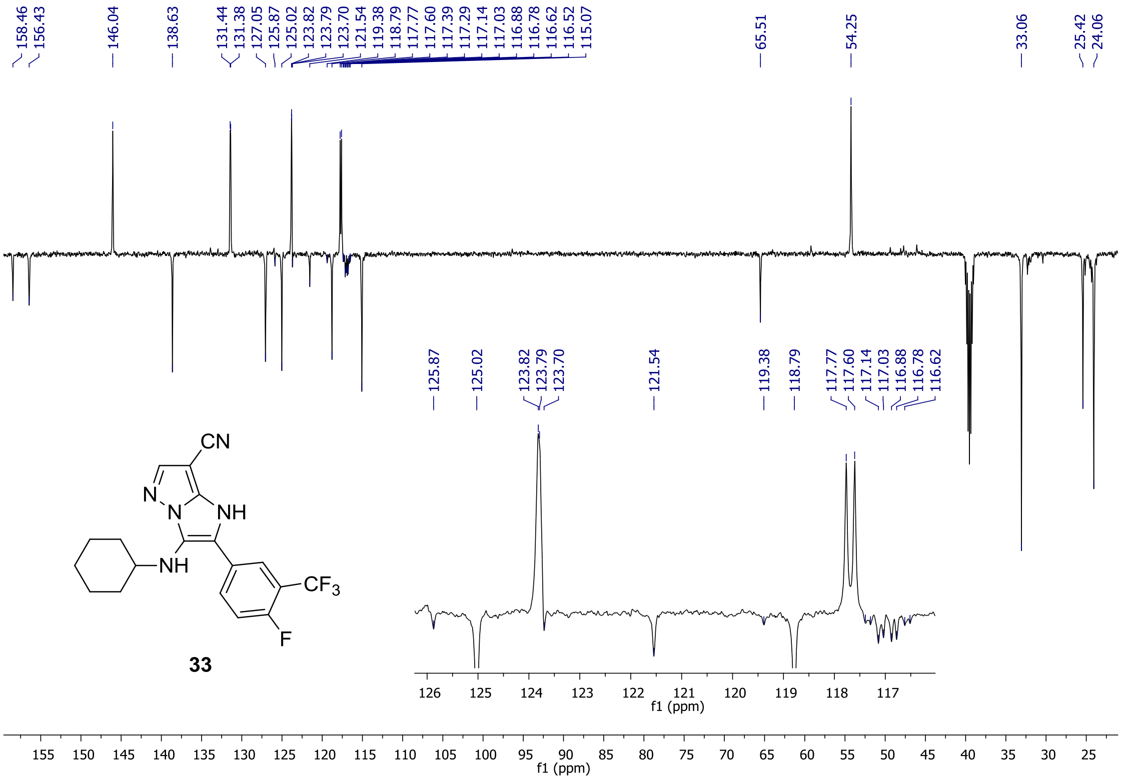

3-(*tert*-Butylamino)-2-(2,4,6-trimethoxyphenyl)-1*H*-imidazo[1,2-*b*]pyrazole-7-carbonitrile (34)

^{1}H NMR (DMSO-d_6, 500 MHz)

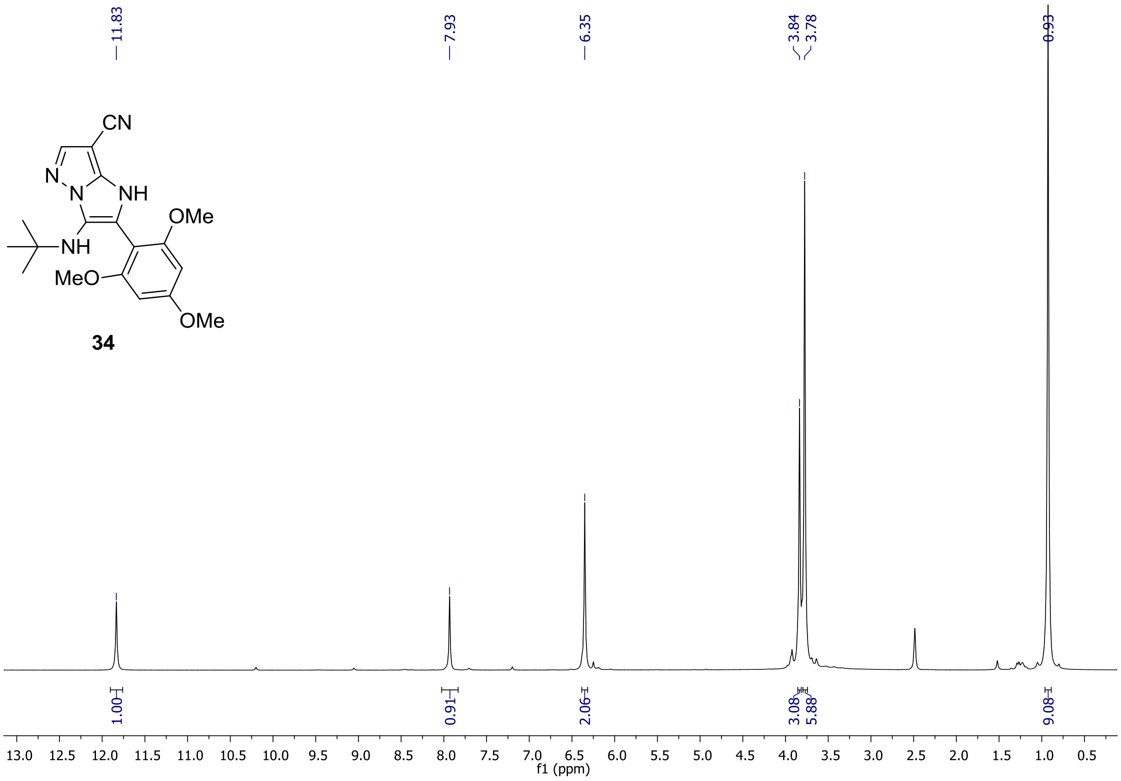

^{13}C NMR (DMSO-d_6, 125 MHz)

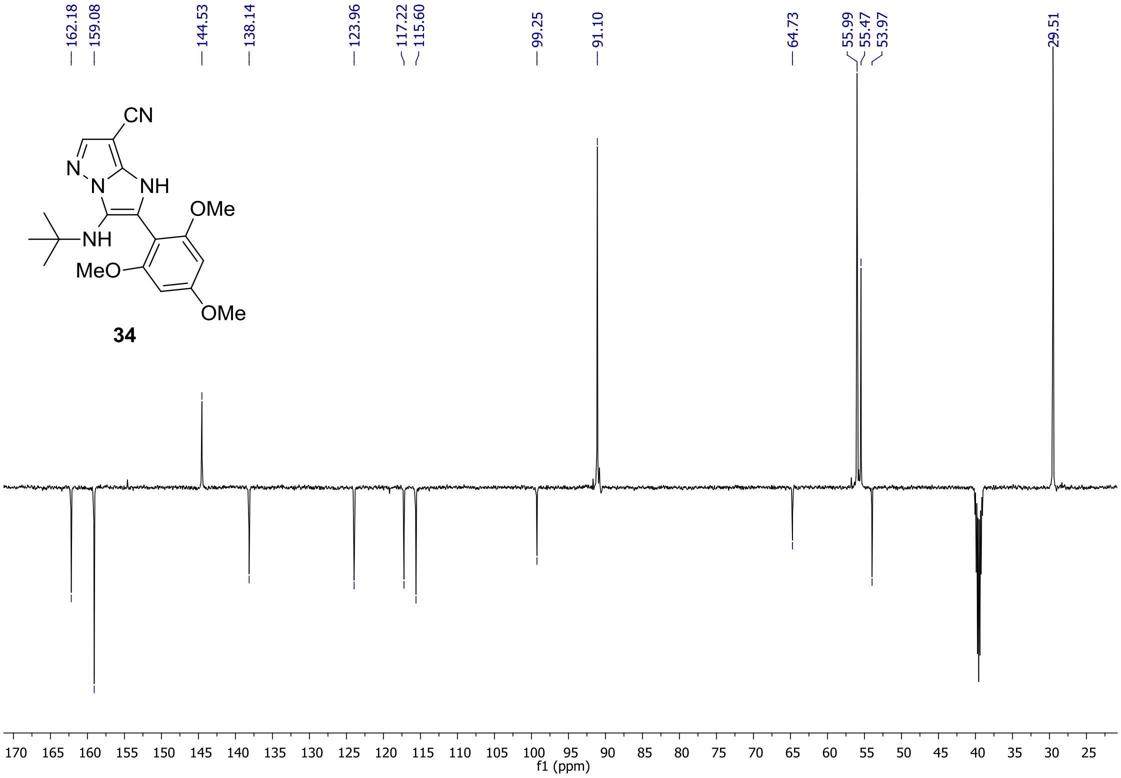

2-(2,4,6-Trimethoxyphenyl)-3-((2,4,4-trimethylpentan-2-yl)amino)-1*H*-imidazo[1,2-*b*]pyrazole-7-carbonitrile (35)

^{1}H NMR (DMSO-d_6, 500 MHz)

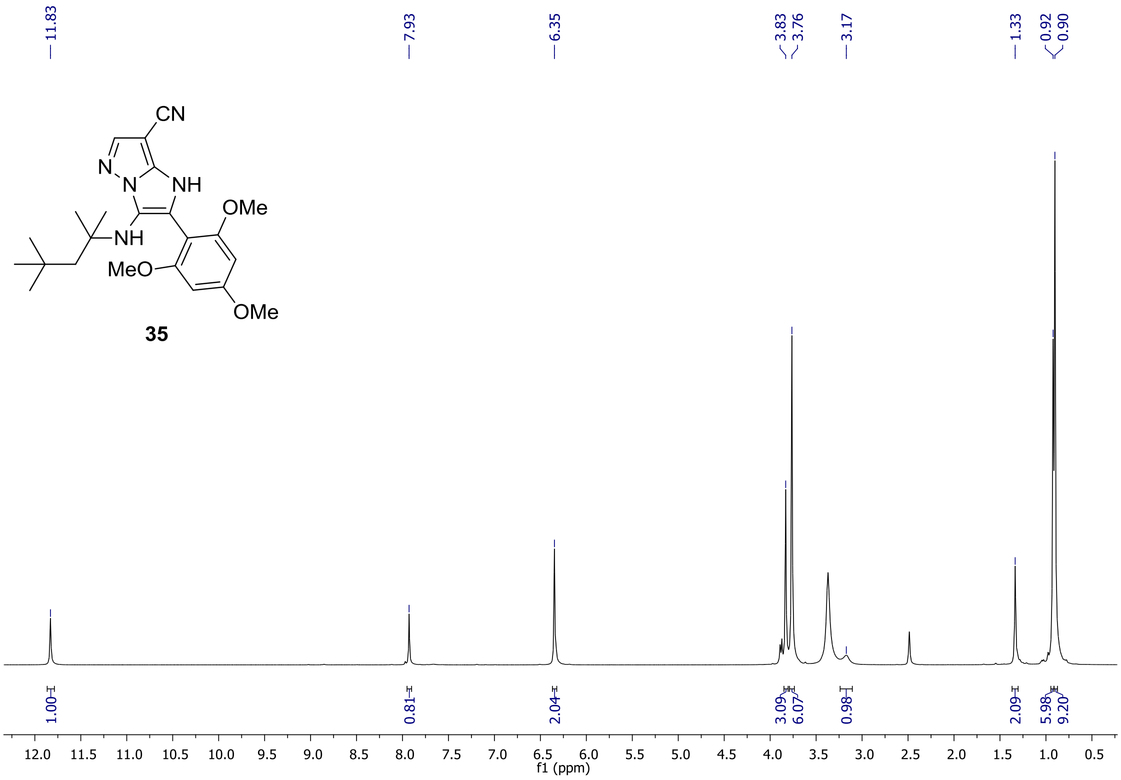

^{13}C NMR (DMSO-d_6, 125 MHz)

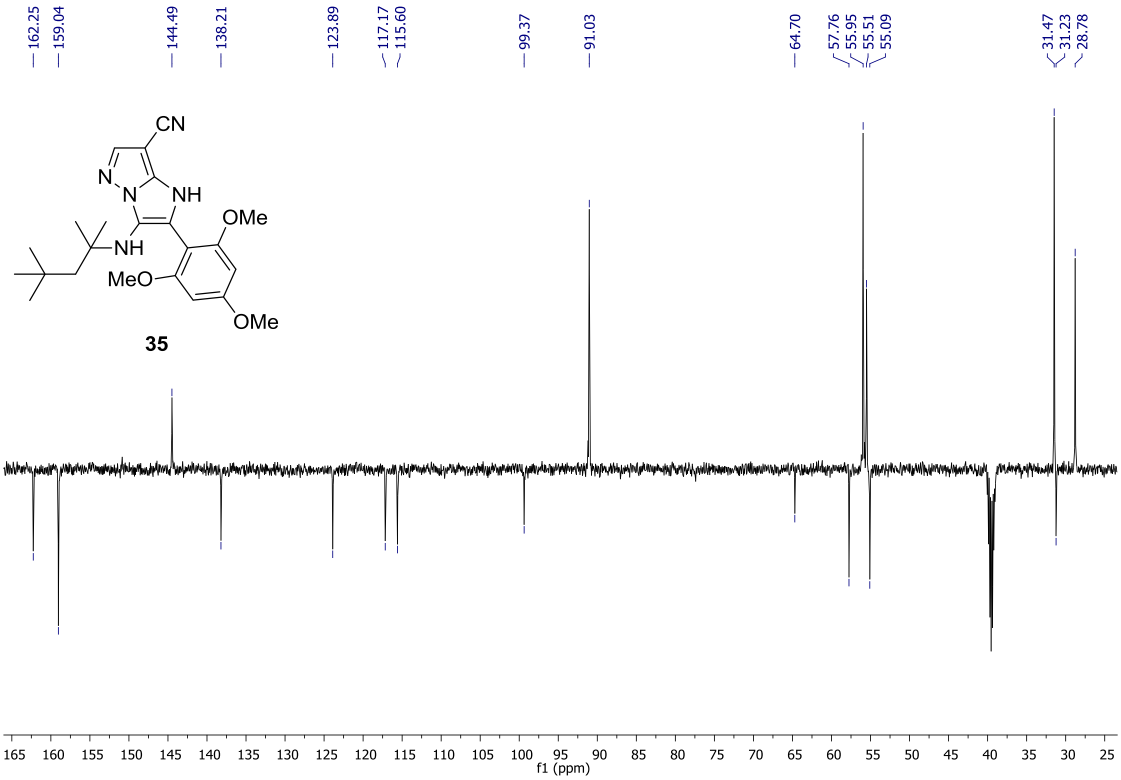

Methyl 2-((7-cyano-2-(2,4,6-trimethoxyphenyl)-1*H*-imidazo[1,2-*b*]pyrazol-3-yl)amino)-acetate (36)

^{1}H NMR (DMSO-d_6, 500 MHz)

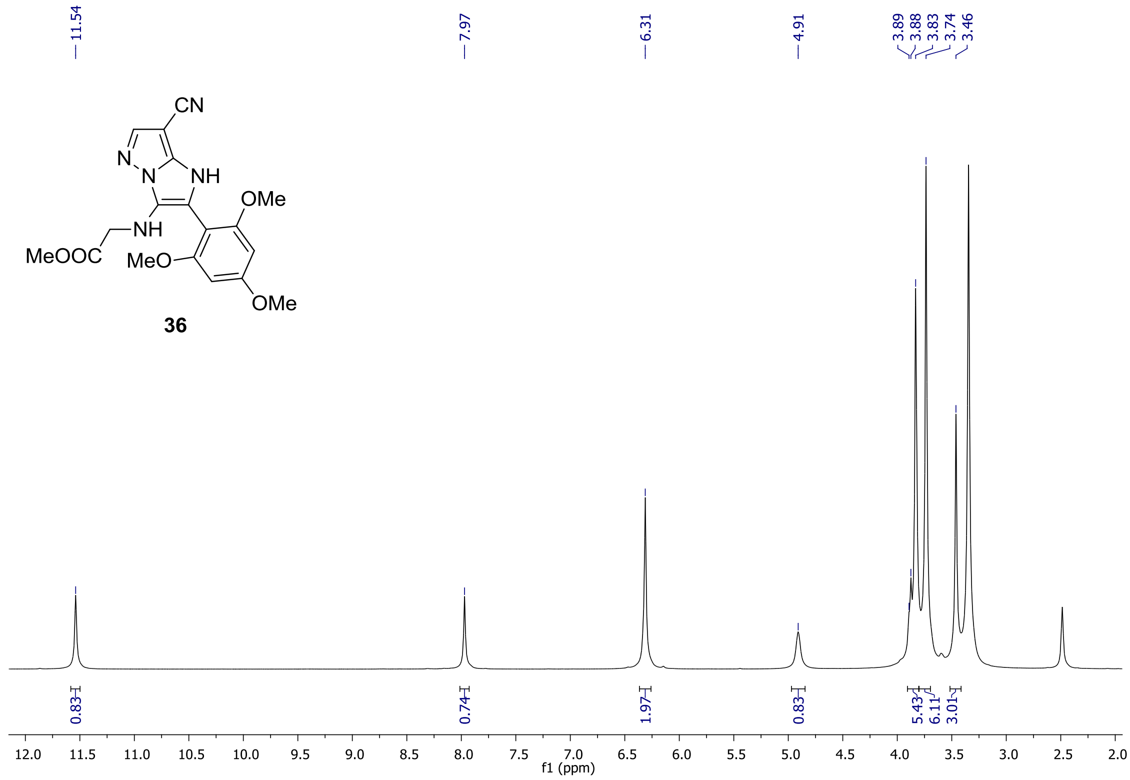

^{13}C NMR (DMSO-d_6, 125 MHz)

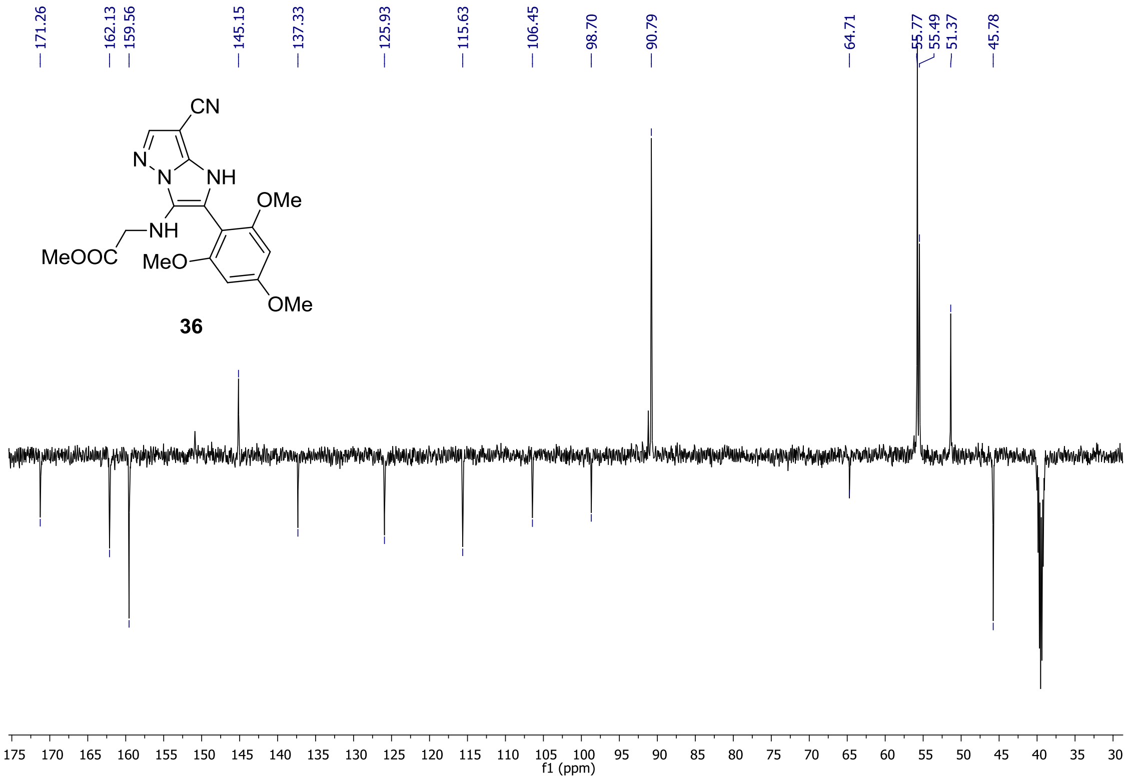

3-(Cyclohexylamino)-2-(2,4,6-trimethoxyphenyl)-1*H*-imidazo[1,2-*b*]pyrazole-7-carbonitrile (37)

^{1}H NMR (DMSO-d_6, 500 MHz)

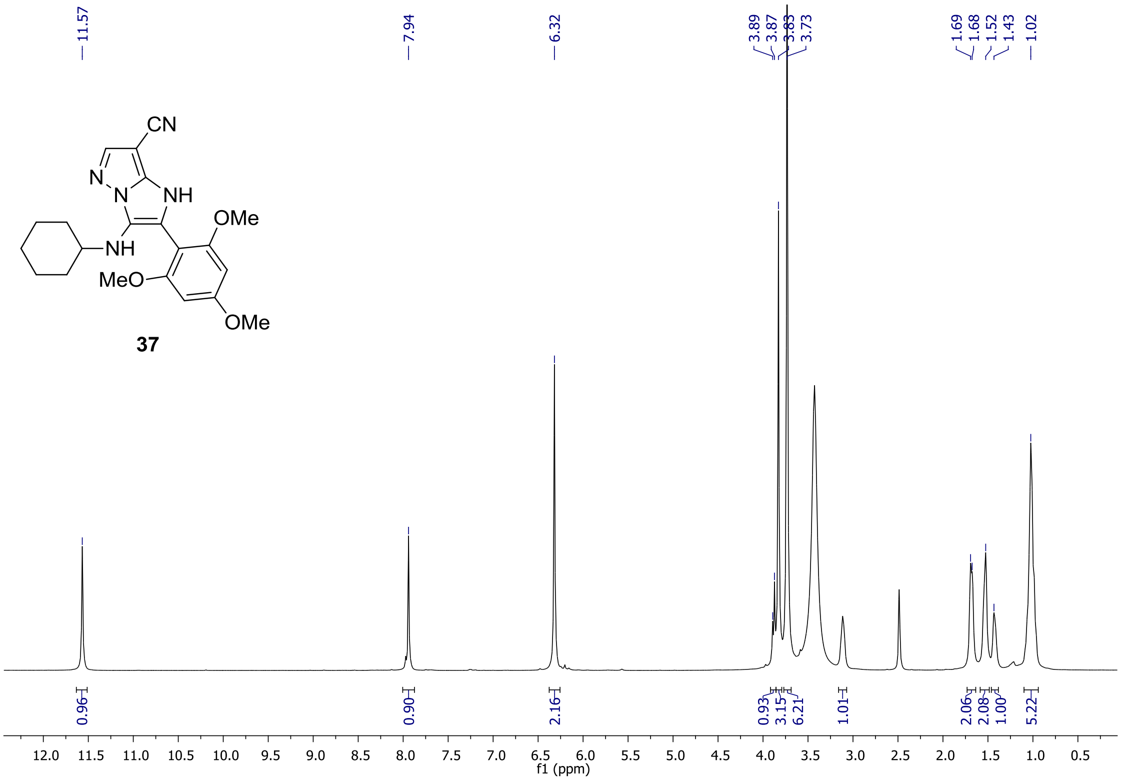

^{13}C NMR (DMSO-d_6, 125 MHz)

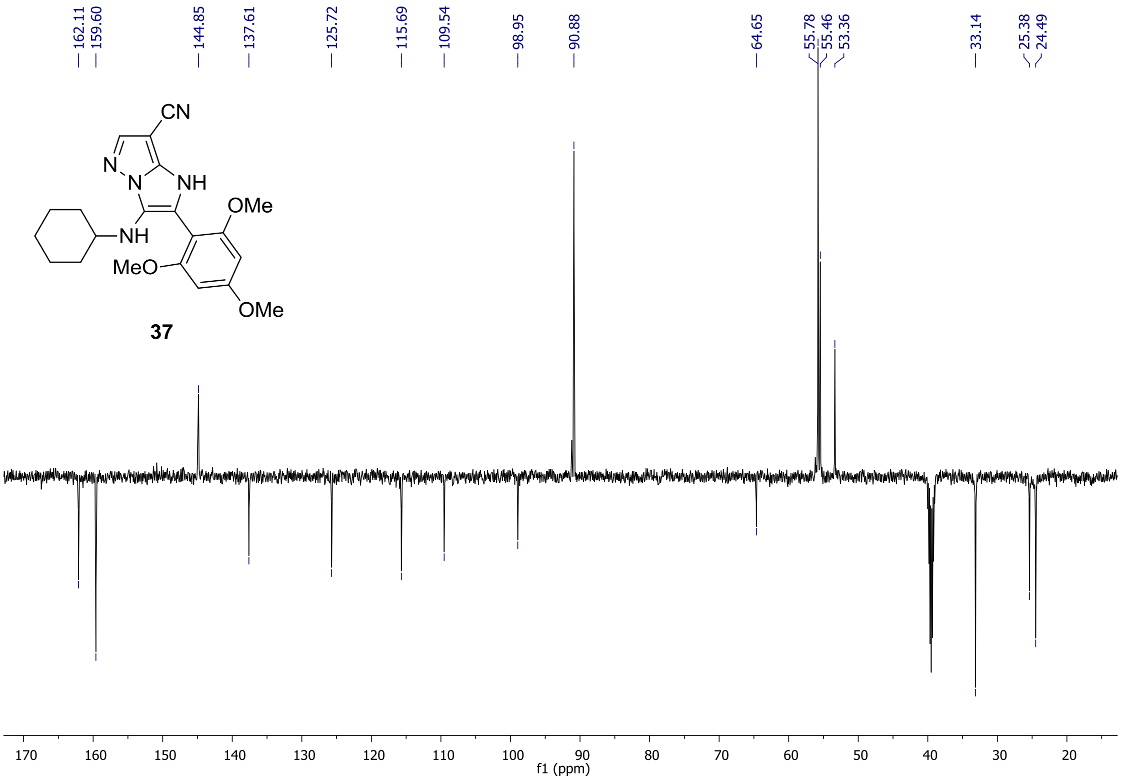

(*E*)-3-(*tert*-Butylamino)-2-(1-phenylprop-1-en-2-yl)-1*H*-imidazo[1,2-*b*]pyrazole-7-carbonitrile (38)

^{1}H NMR (DMSO-d_6, 500 MHz)

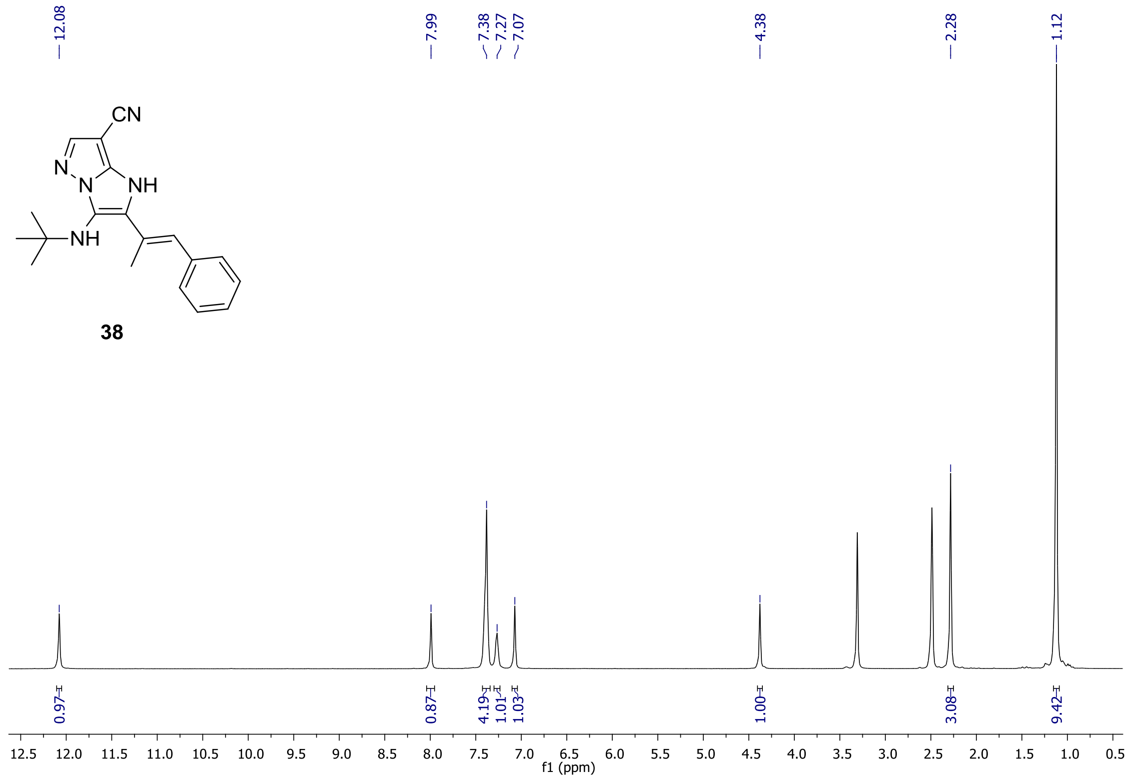

^{13}C NMR (DMSO-d_6, 125 MHz)

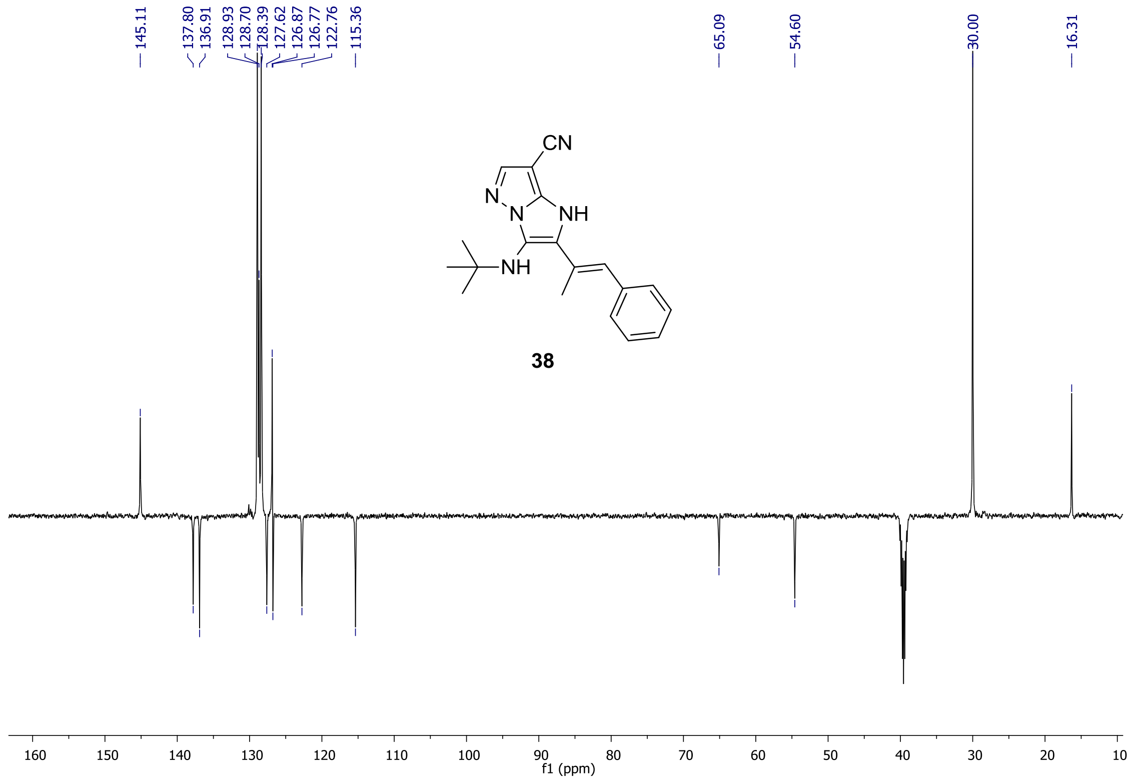

(*E*)-2-(1-Phenylprop-1-en-2-yl)-3-((2,4,4-trimethylpentan-2-yl)amino)-1*H*-imidazo[1,2-*b*]pyrazole-7-carbonitrile (39)

^{1}H NMR (DMSO-d_6, 500 MHz)

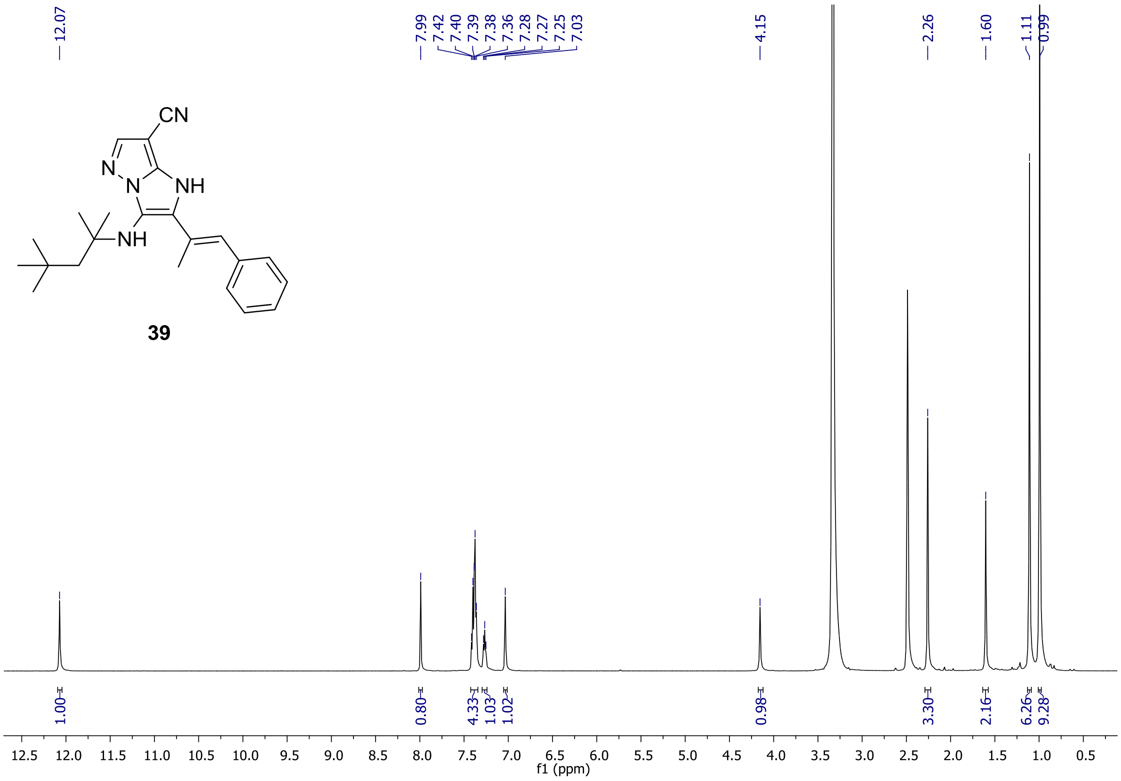

^{13}C NMR (DMSO-d_6, 125 MHz)

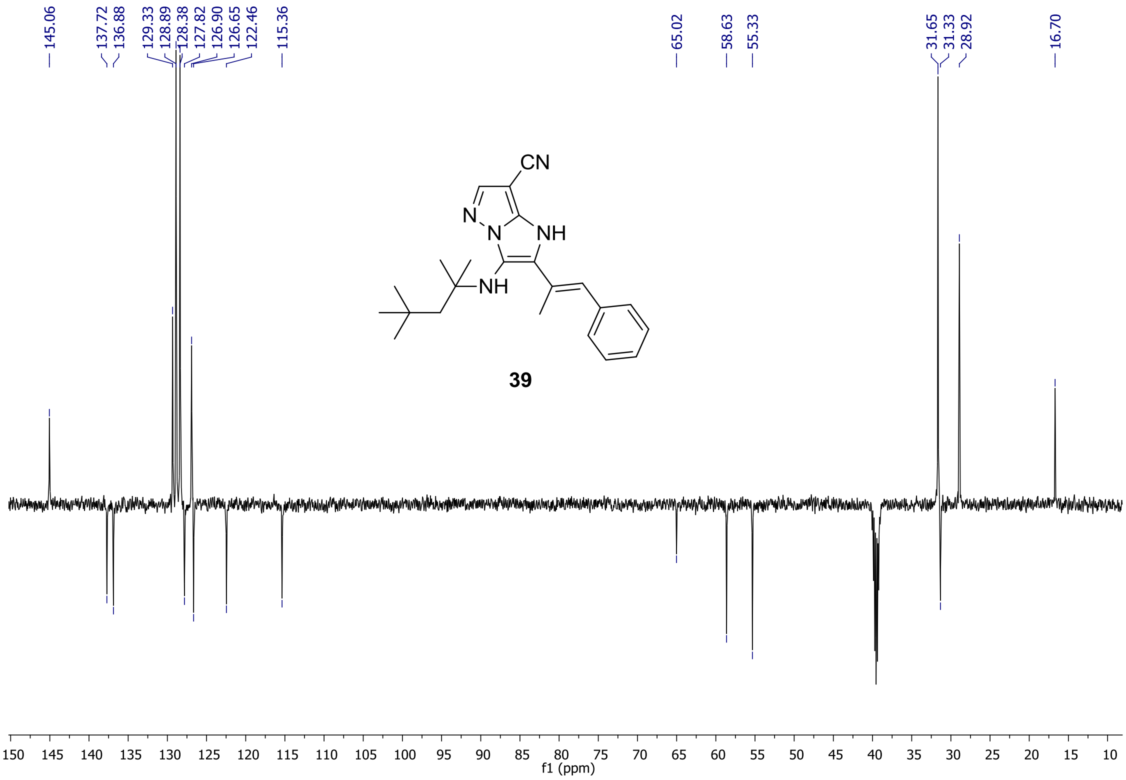

(*E*)-Methyl 2-((7-cyano-2-(1-phenylprop-1-en-2-yl)-1*H*-imidazo[1,2-*b*]pyrazole-3-yl)-amino)acetate (40)

^{1}H NMR (DMSO-d_6, 500 MHz)

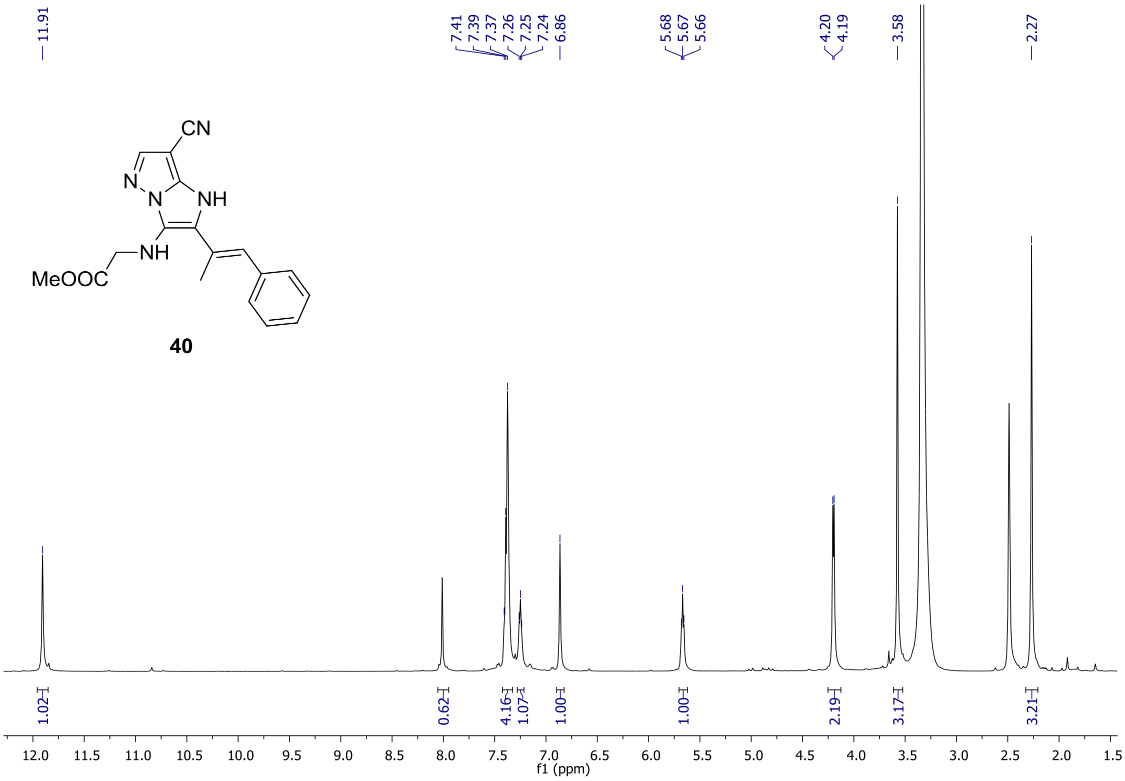

^{13}C NMR (DMSO-d_6, 125 MHz)

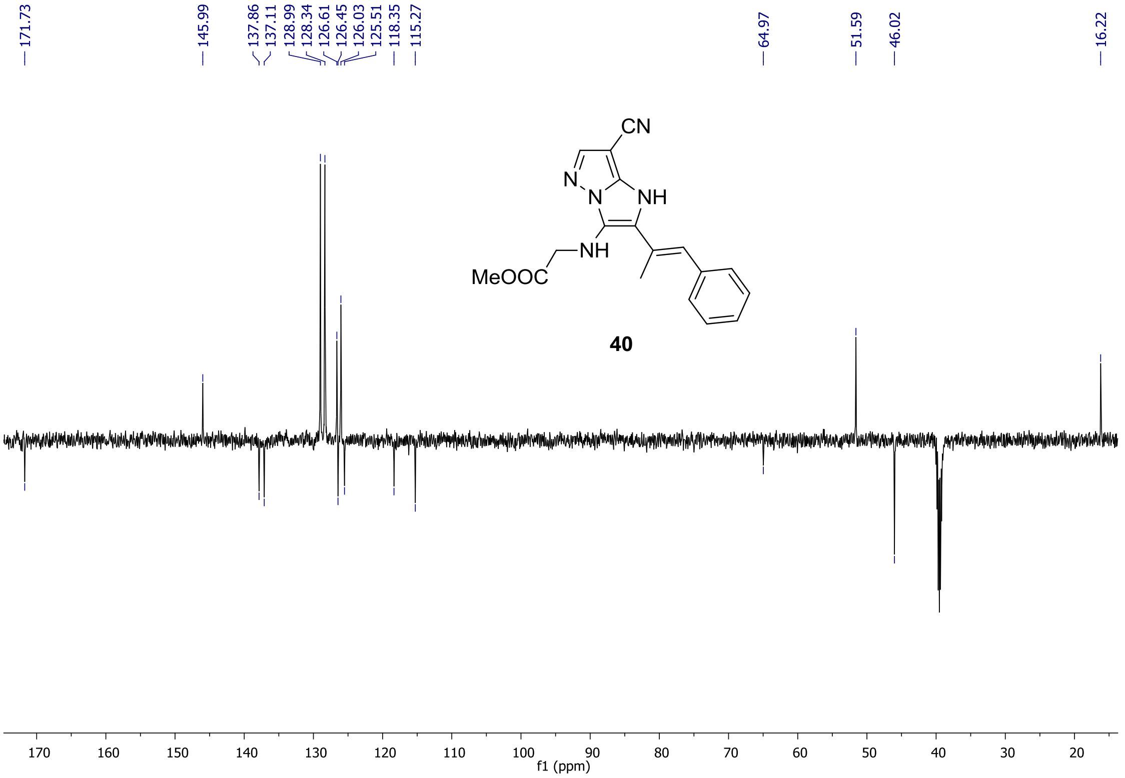

(*E*)-3-(Cyclohexylamino)-2-(1-phenylprop-1-en-2-yl)-1*H*-imidazo[1,2-*b*]pyrazole-7-carbonitrile (41)

^{1}H NMR (DMSO-d_6, 500 MHz)

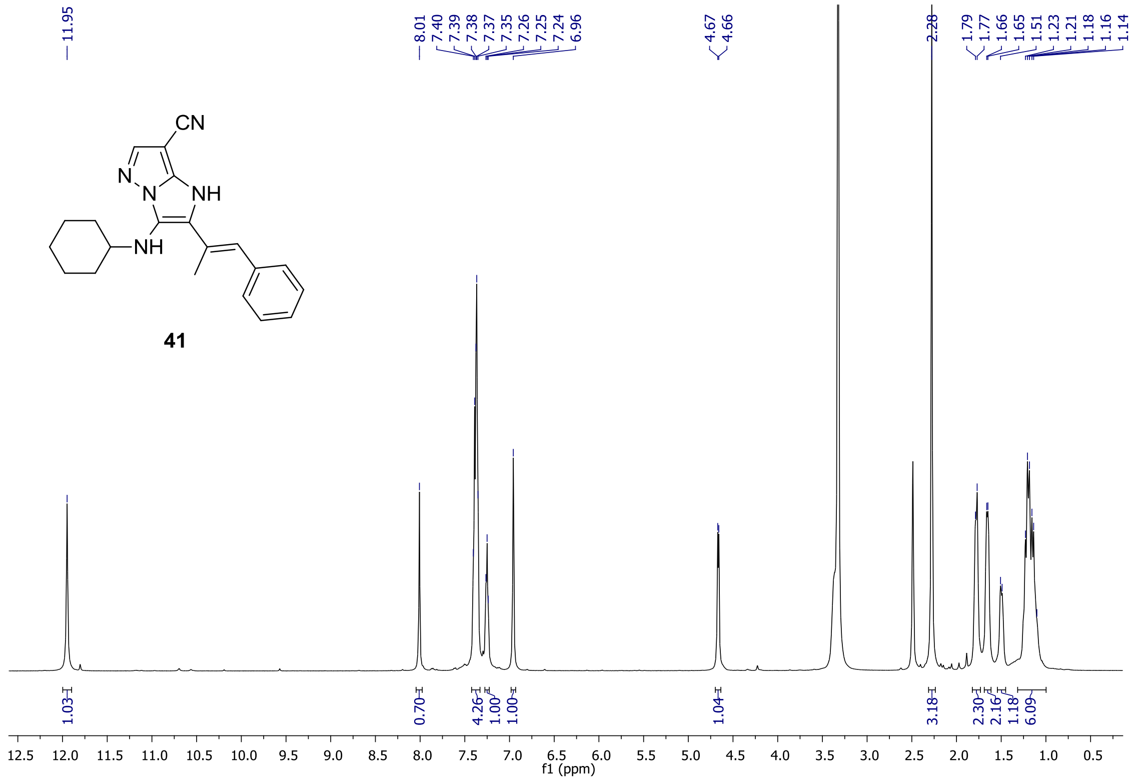

^{13}C NMR (DMSO-d_6, 125 MHz)

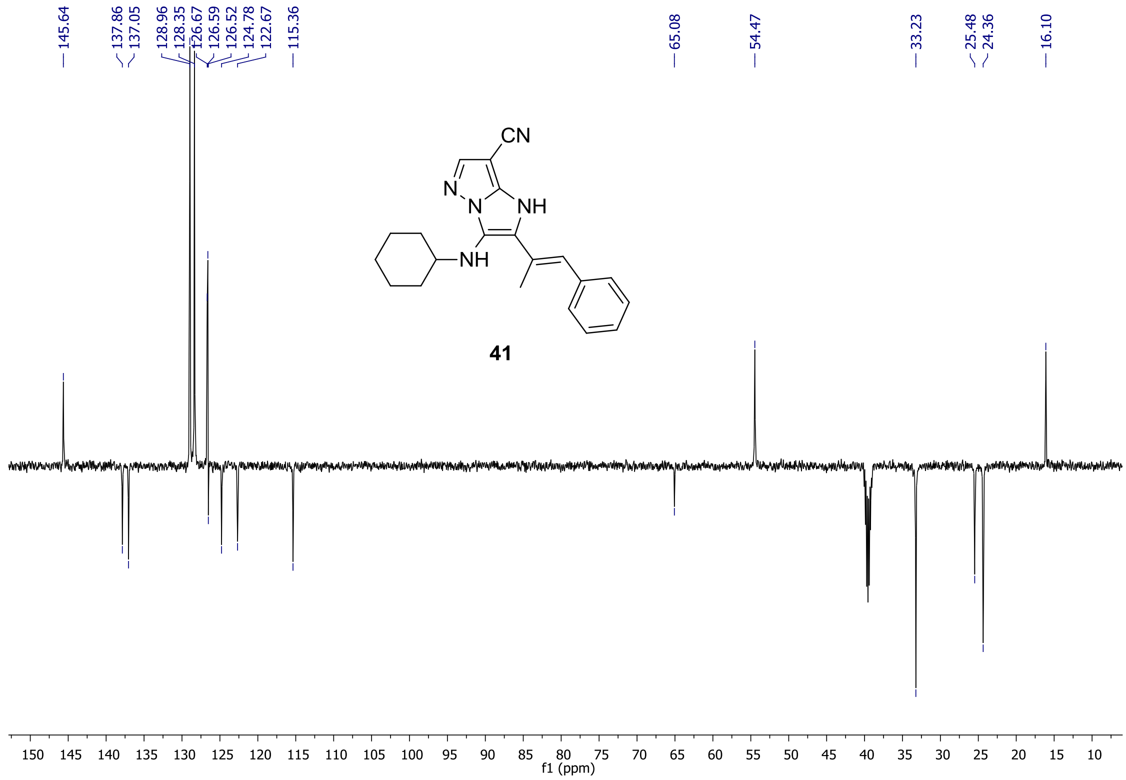

2-(*tert*-Butyl)-3-(*tert*-butylamino)-1*H*-imidazo[1,2-*b*]pyrazole-7-carbonitrile (42)

^{1}H NMR (DMSO-d_6, 500 MHz)

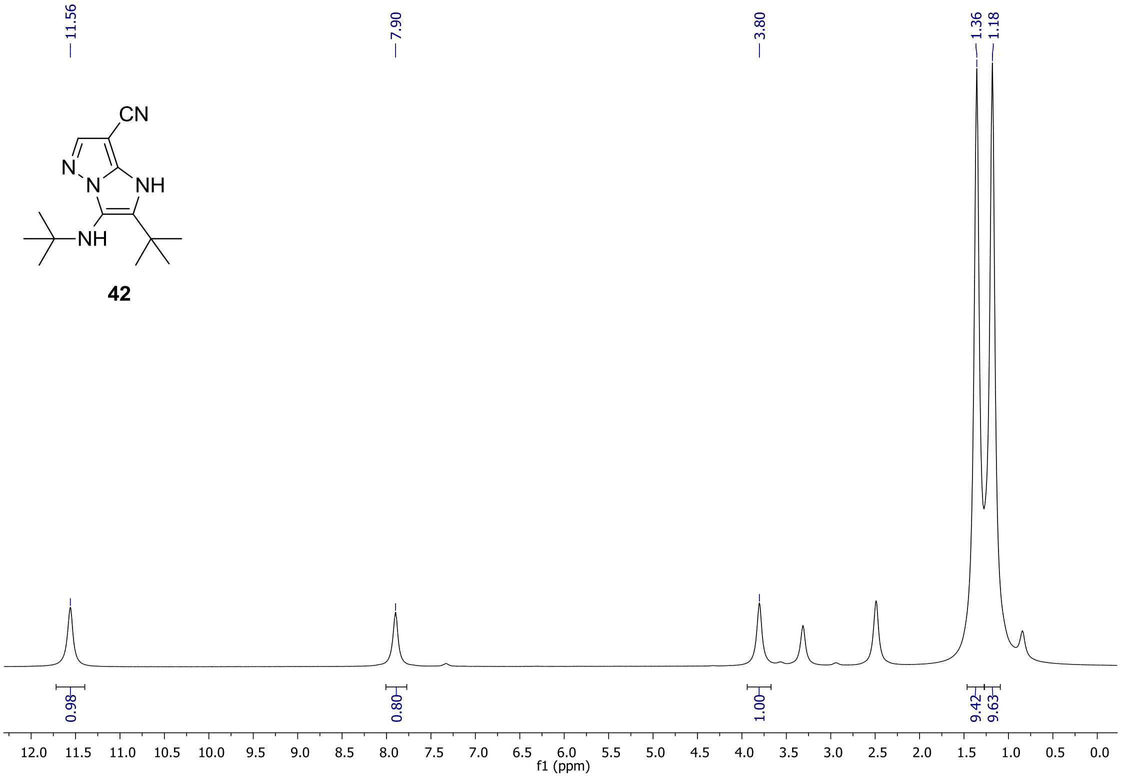

^{13}C NMR (DMSO-d_6, 125 MHz)

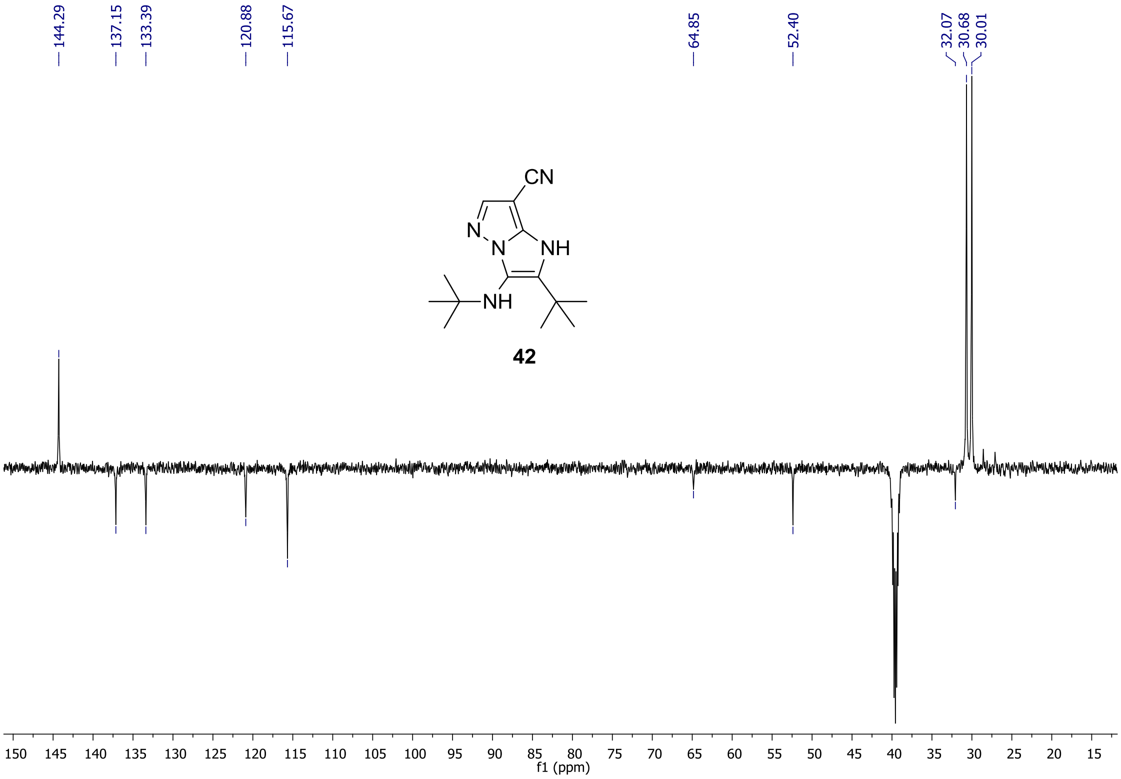

2-(*tert*-Butyl)-3-((2,4,4-trimethylpentan-2-yl)amino)-1*H*-imidazo[1,2-*b*]pyrazole-7-carbonitrile (43)

^{1}H NMR (DMSO-d_6, 500 MHz)

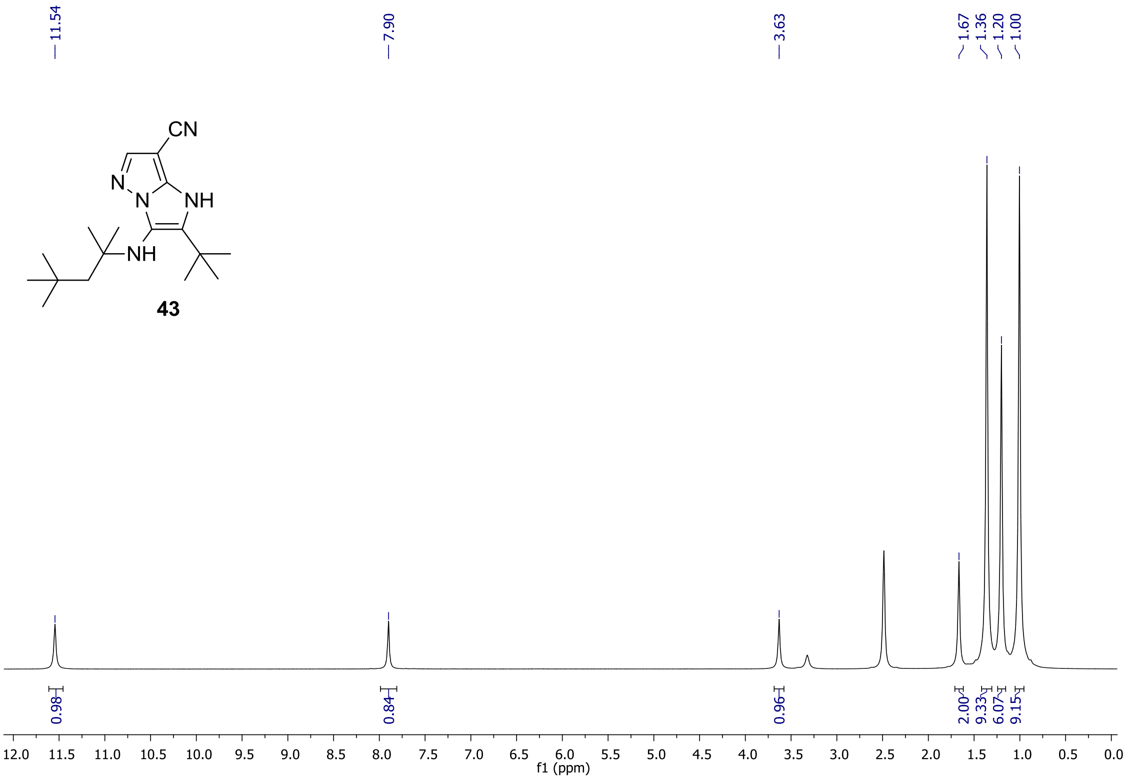

^{13}C NMR (DMSO-d_6, 125 MHz)

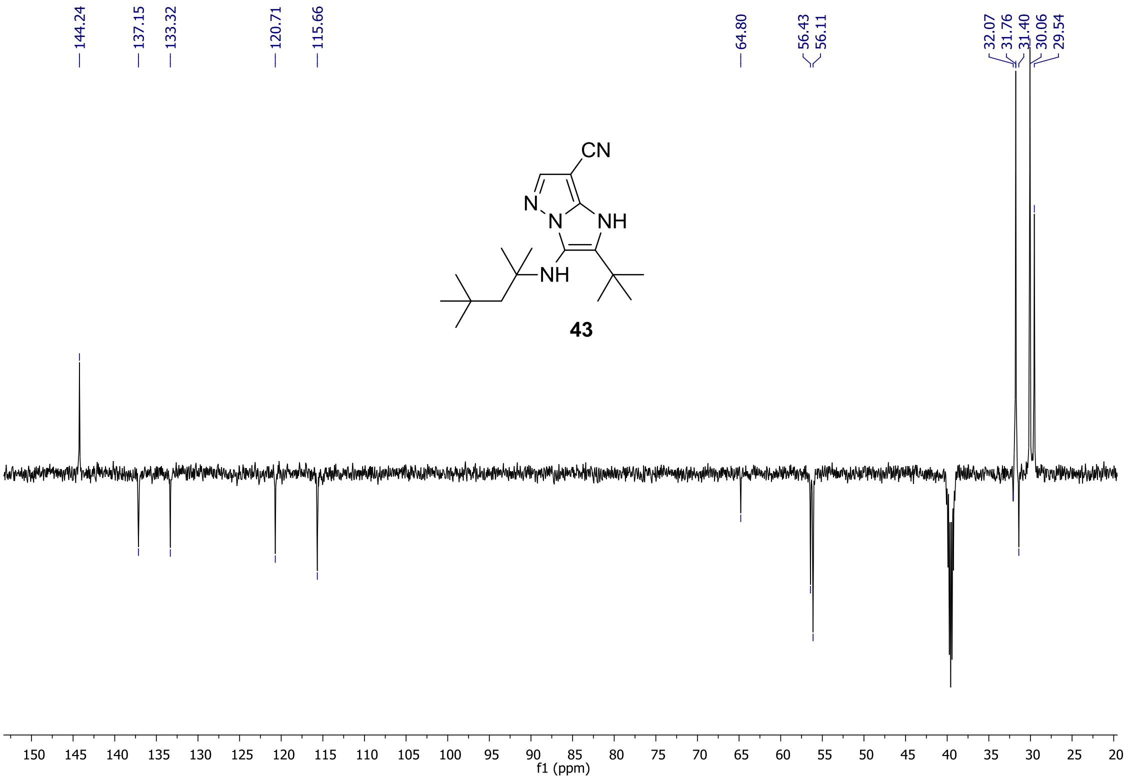

Methyl 2-((2-(*tert*-butyl)-7-cyano-1*H*-imidazo[1,2-*b*]pyrazole-3-yl)amino)acetate (44)

^{1}H NMR (DMSO-d_6, 500 MHz)

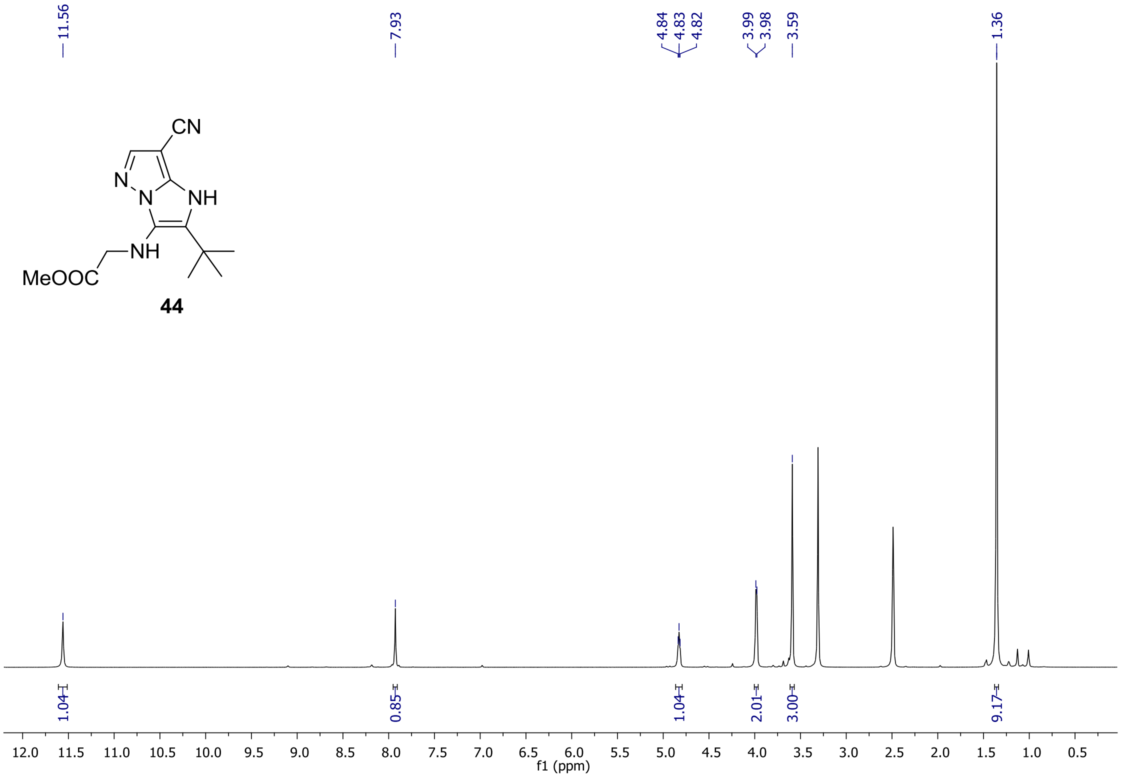

^{13}C NMR (DMSO-d_6, 125 MHz)

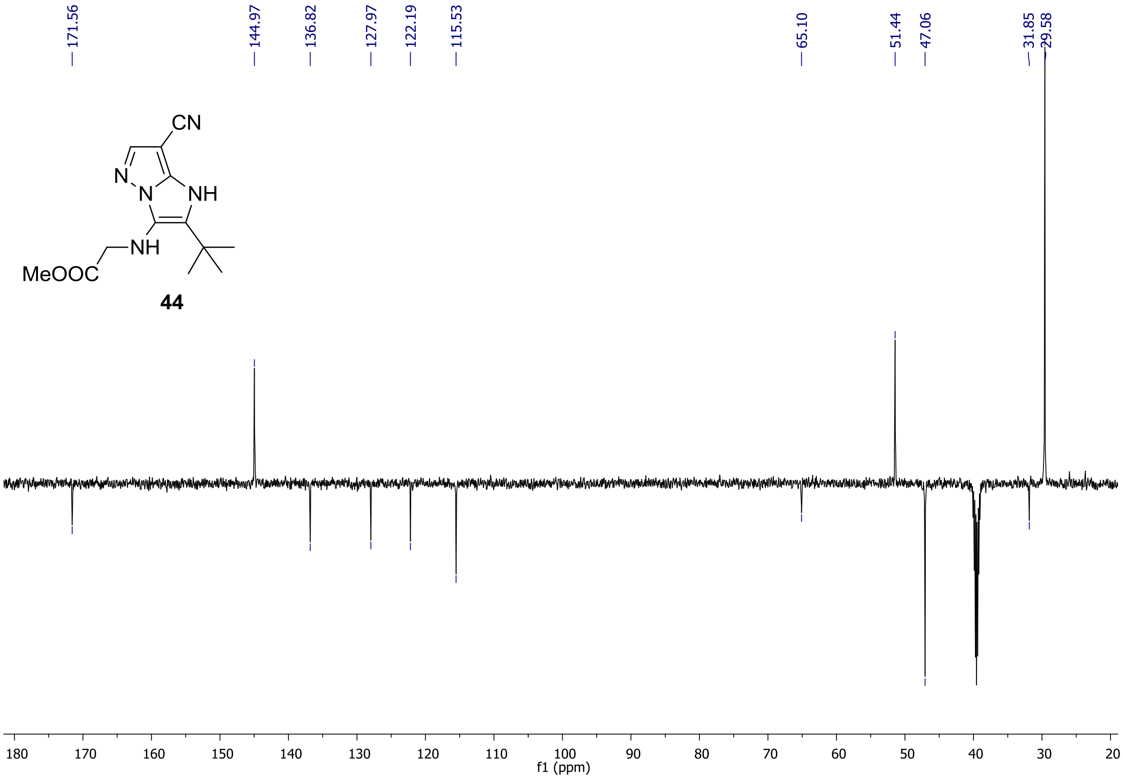

2-(*tert*-Butyl)-3-(cyclohexylamino)-1*H*-imidazo[1,2-*b*]pyrazole-7-carbonitrile (45)

^{1}H NMR (DMSO-d_6, 500 MHz)

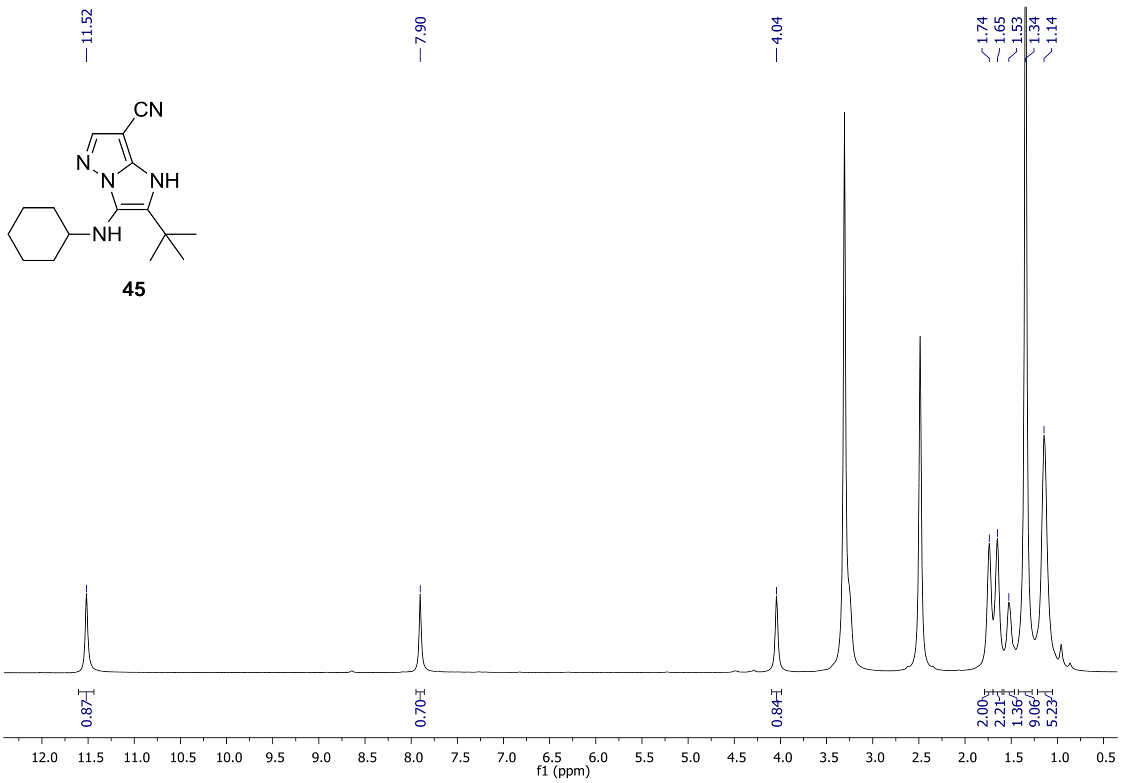

^{13}C NMR (DMSO-d_6, 125 MHz)

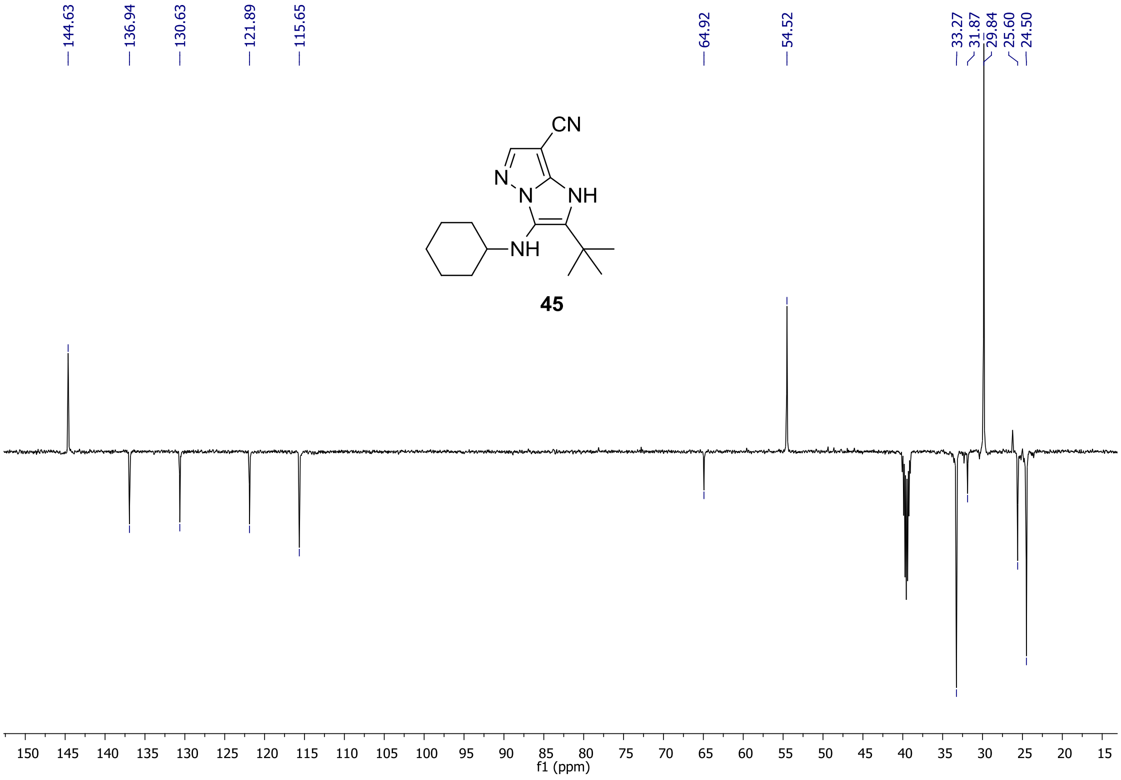

Ethyl 3-(*tert*-butylamino)-2-(*p*-tolyl)-1*H*-imidazo[1,2-*b*]pyrazole-7-carboxylate (46)

^{1}H NMR (DMSO-d_6, 500 MHz)

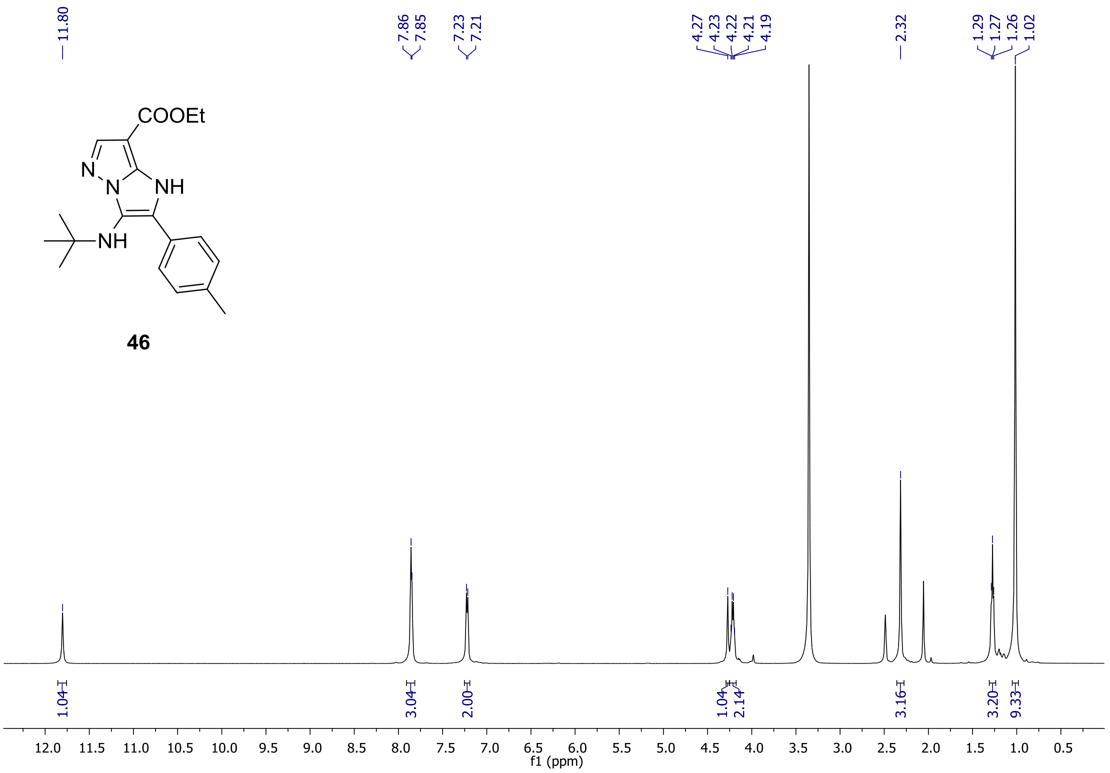

^{13}C NMR (DMSO-d_6, 125 MHz)

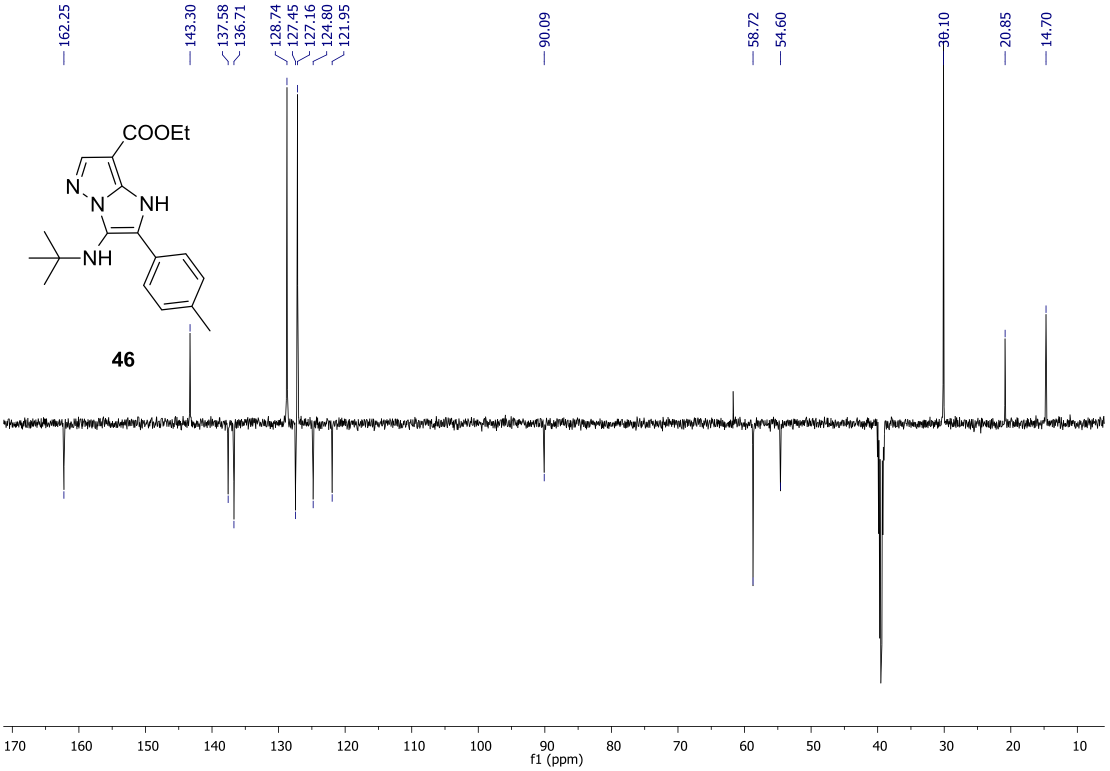

Ethyl 2-phenyl-3-((2,4,4-trimethylpentan-2-yl)amino)-1*H*-imidazo[1,2-*b*]pyrazole-7-carboxylate (47)

^{1}H NMR (DMSO-d_6, 500 MHz)

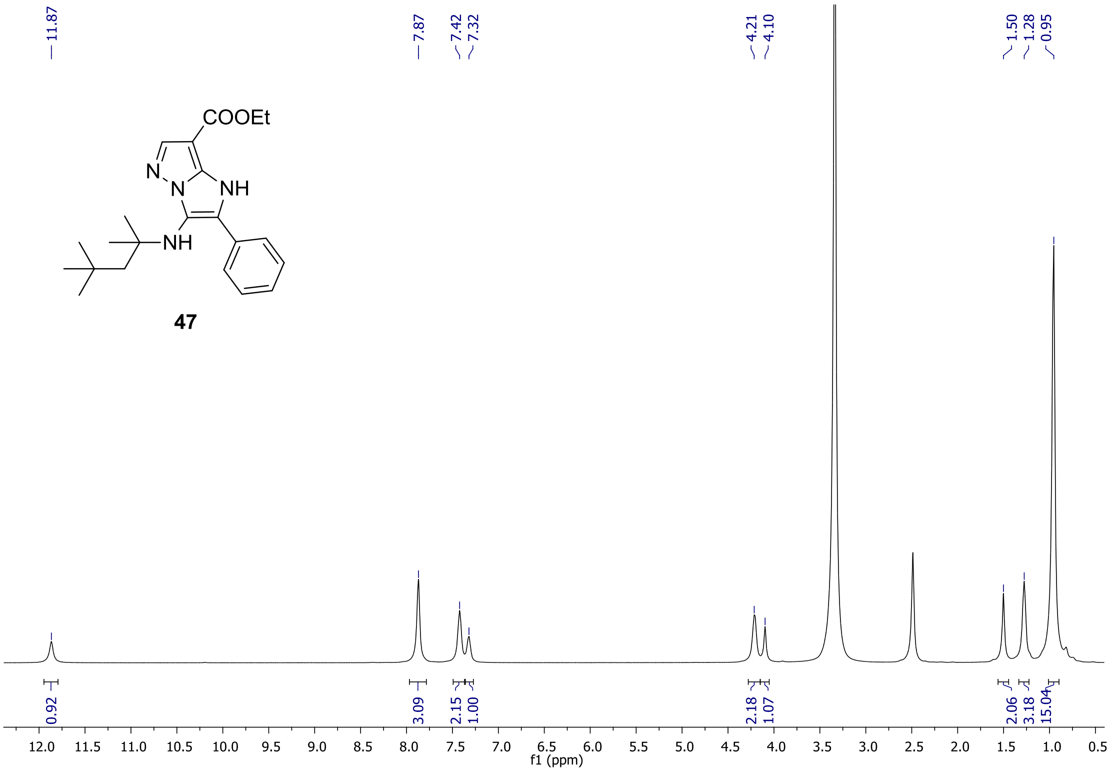

^{13}C NMR (DMSO-d_6, 125 MHz)

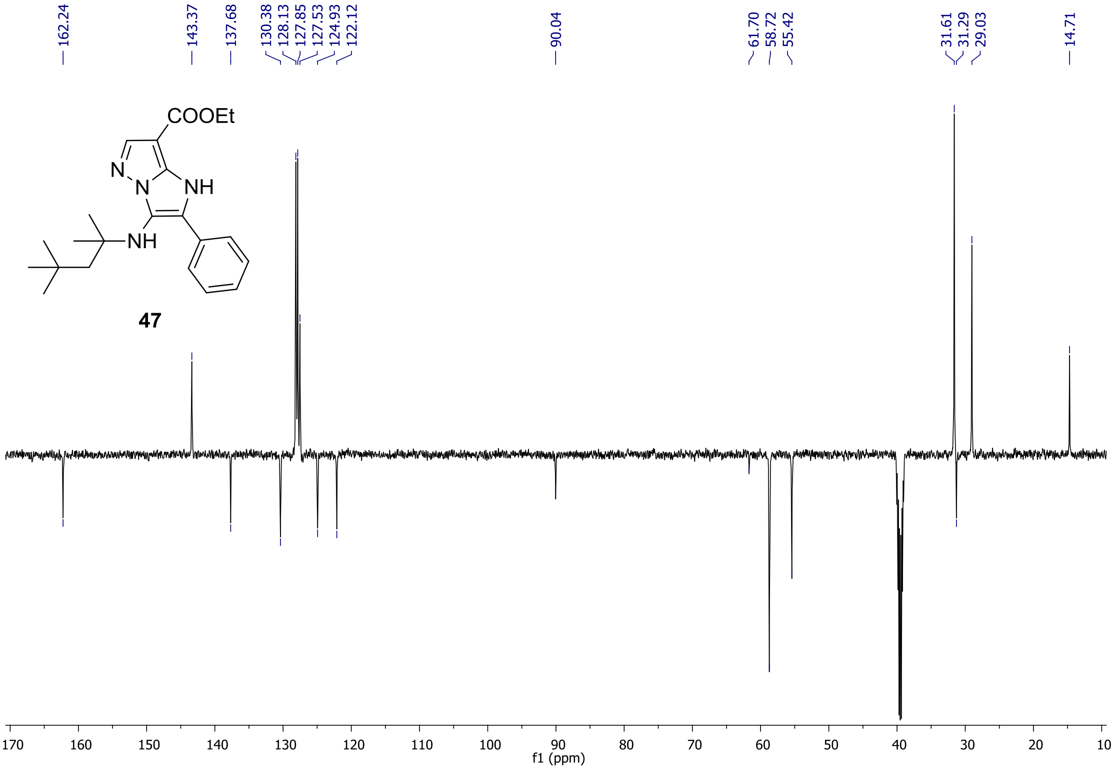

3-(*tert*-Butylamino)-6-methyl-2-(*p*-tolyl)-1*H*-imidazo[1,2-*b*]pyrazole-7-carbonitrile (48)

^{1}H NMR (DMSO-d_6, 500 MHz)

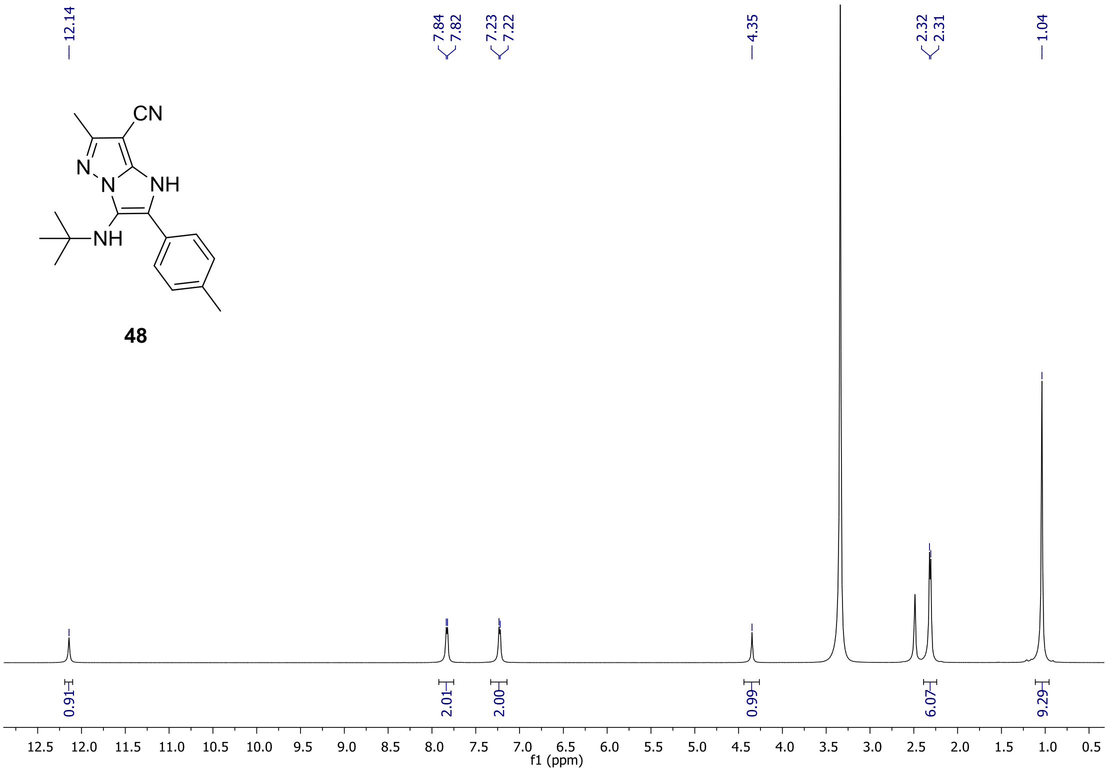

^{13}C NMR (DMSO-d_6, 125 MHz)

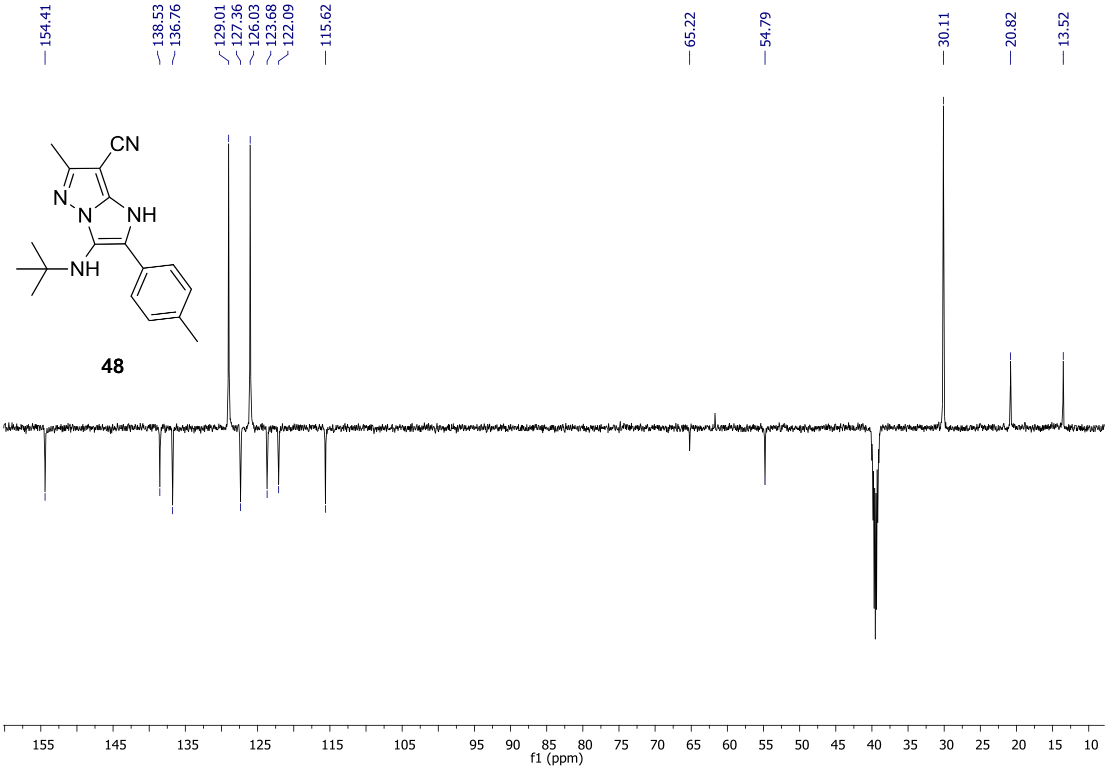

Methyl 2-((7-Cyano-2-(4-fluorophenyl)-6-methyl-1*H*-imidazo[1,2-*b*]pyrazol-3-yl)amino)-acetate (49)

^{1}H NMR (DMSO-d_6, 500 MHz)

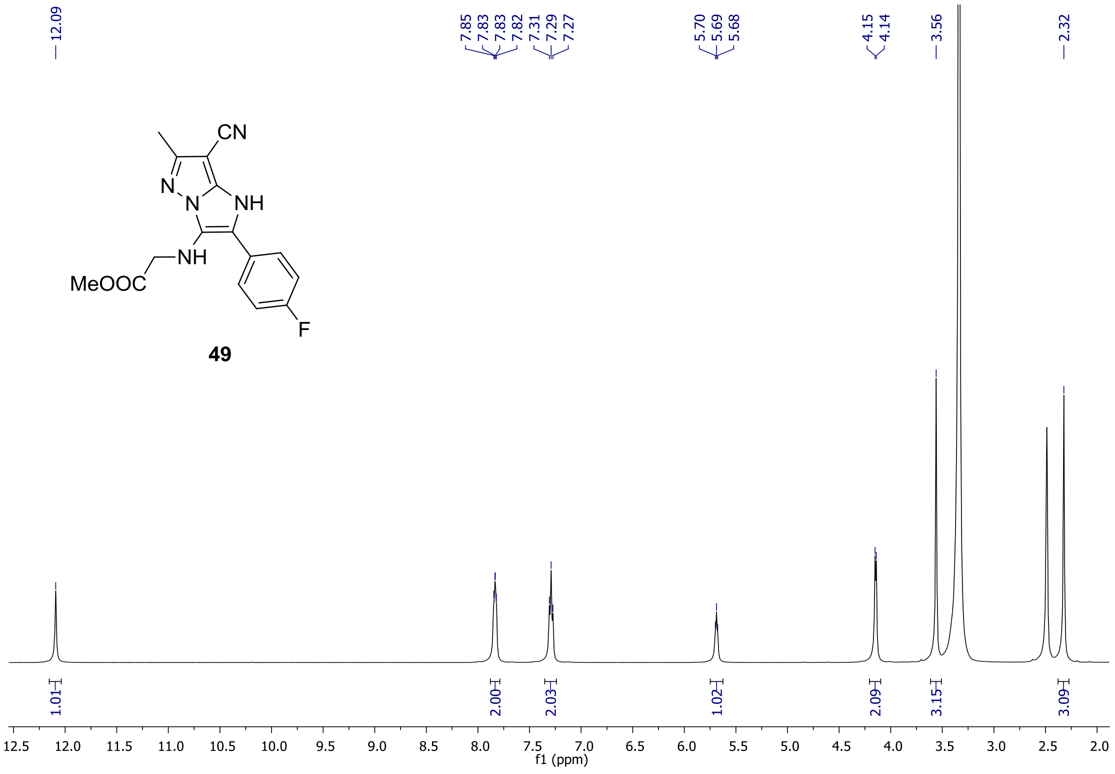

^{13}C NMR (DMSO-d_6, 125 MHz)

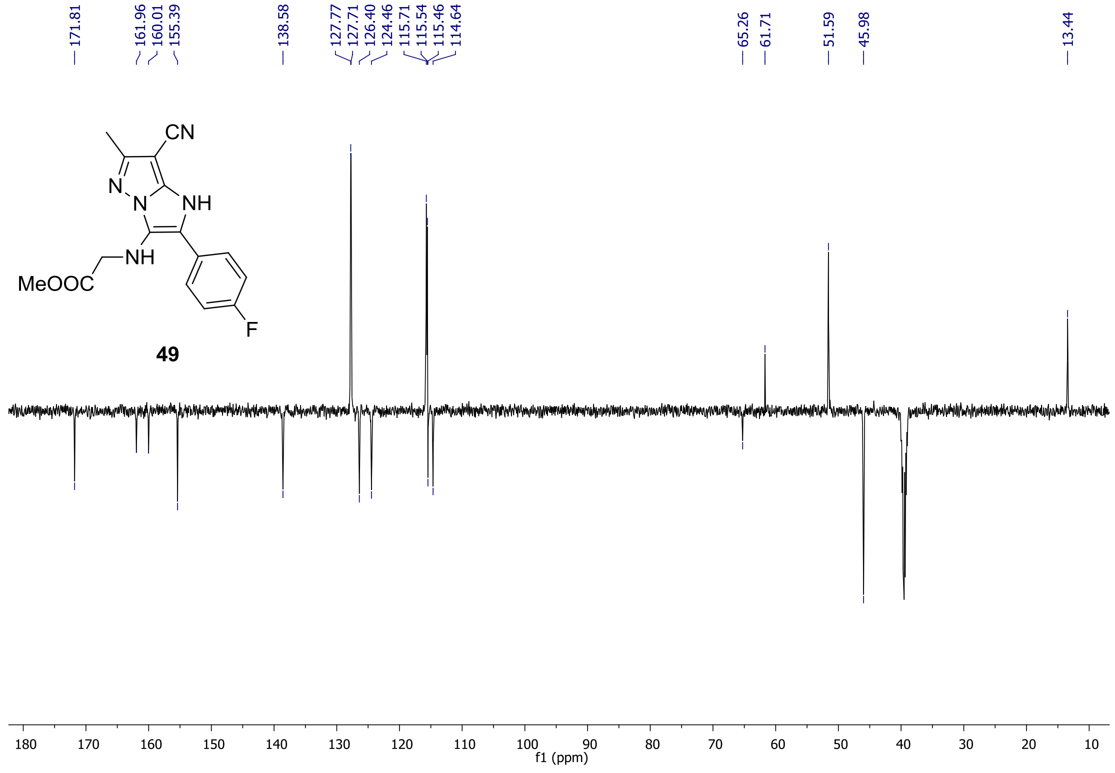

Ethyl 3-(*tert*-butylamino)-6-methyl-2-(*p*-tolyl)-1*H*-imidazo[1,2-*b*]pyrazole-7-carboxylate (50)

^{1}H NMR (DMSO-d_6, 500 MHz)

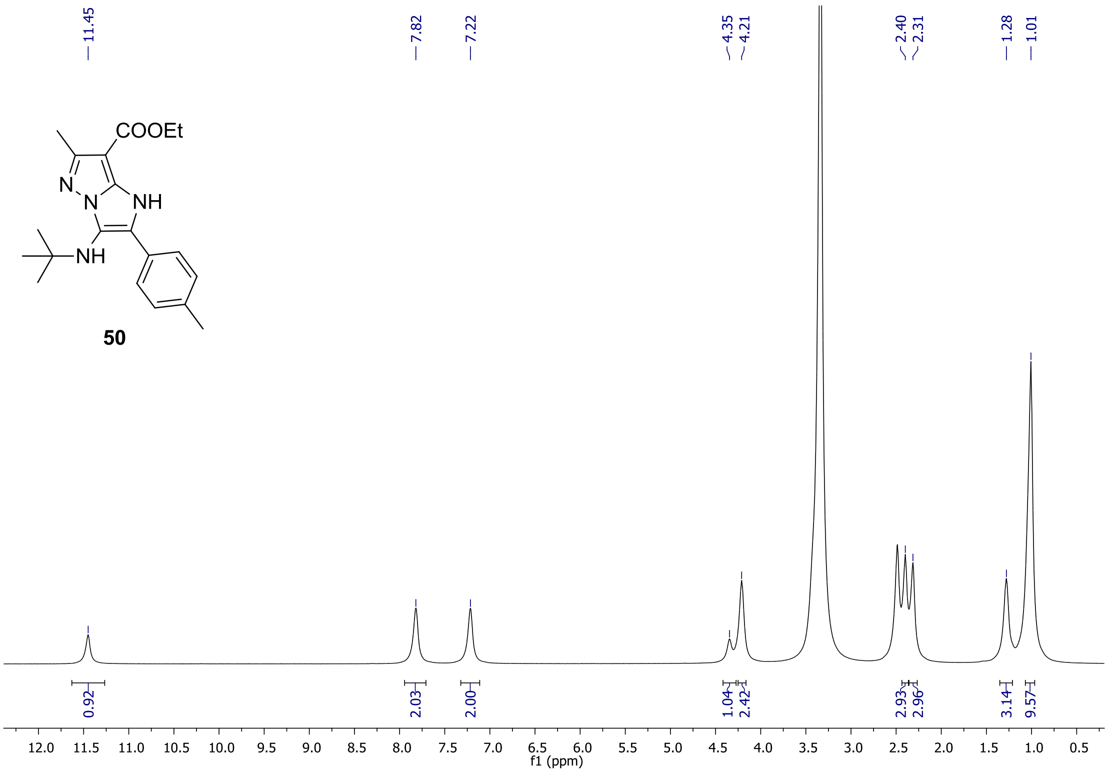

^{13}C NMR (DMSO-d_6, 125 MHz)

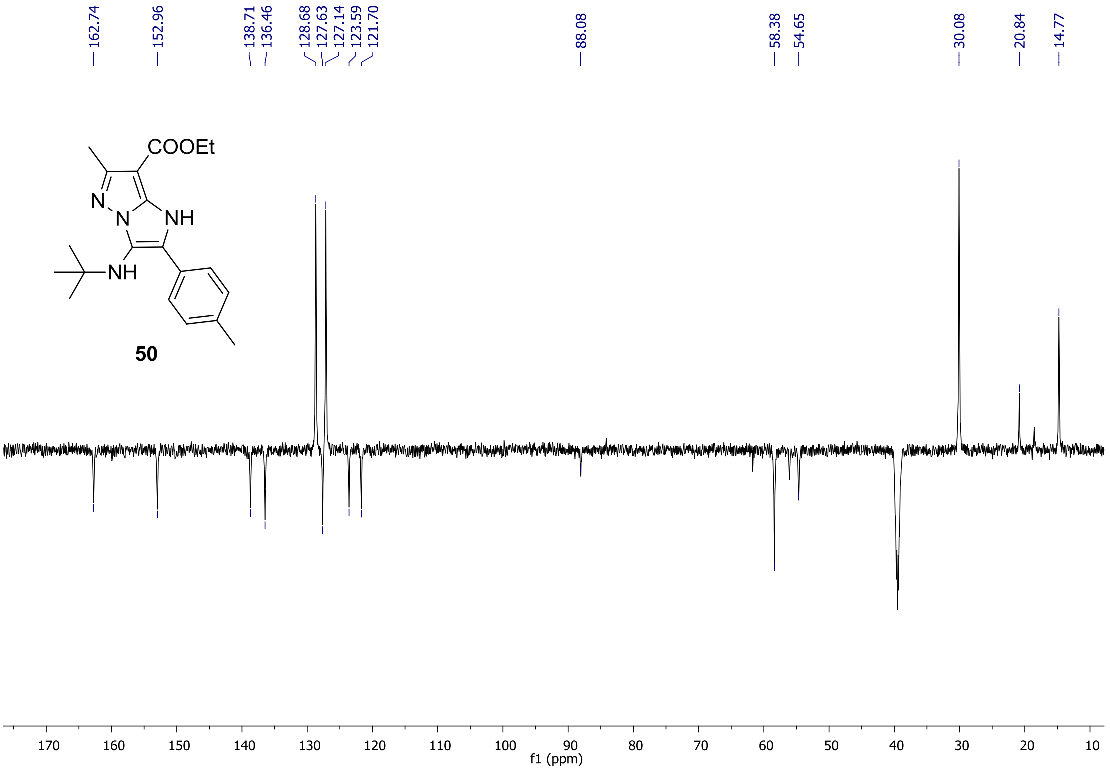

(E)-Ethyl 6-methyl-2-(1-phenylprop-1-en-2-yl)-3-((2,4,4-trimethylpentan-2-yl)amino)-1*H*-imidazo[1,2-*b*]pyrazole-7-carboxylate (51)

^{1}H NMR (DMSO-d_6, 500 MHz)

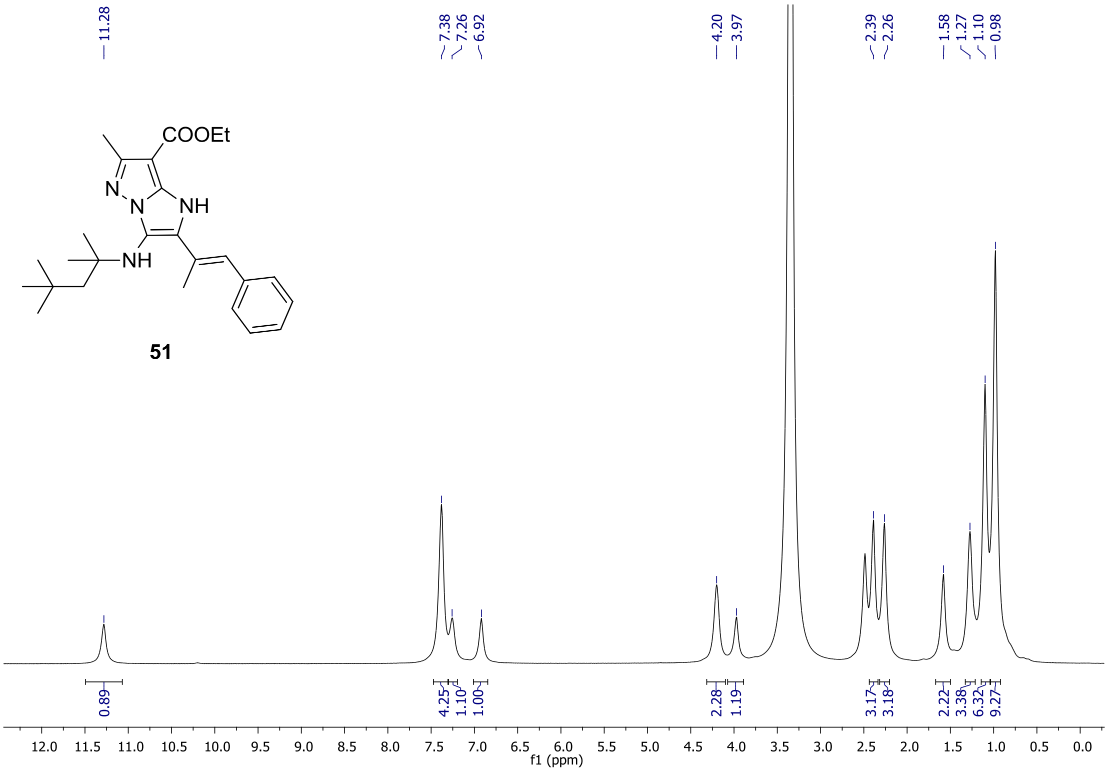

^{13}C NMR (DMSO-d_6, 125 MHz)

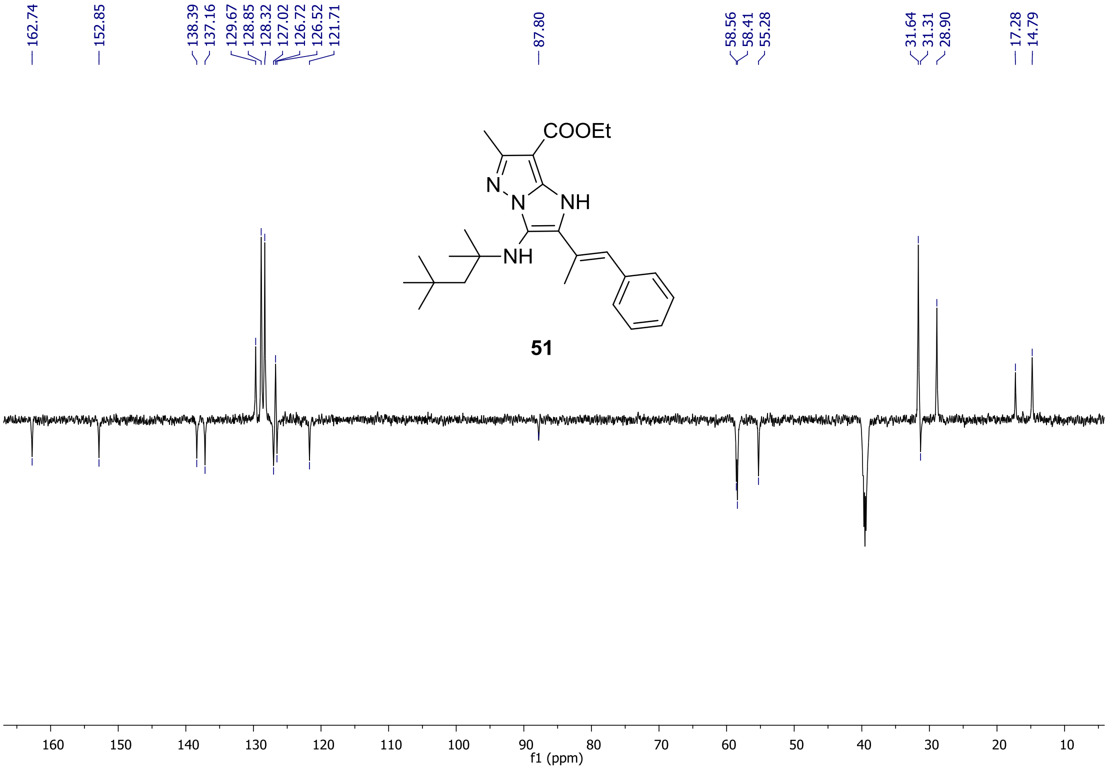

Supplement: File 1 — Experimental and characterisation data. [file Beilstein_J_Org_Chem-10-2338-s001.pdf]
